# Efficacy and Safety of COVID-19 Vaccines: A Systematic Review and Meta-Analysis of Randomized Clinical Trials

**DOI:** 10.3390/vaccines9050467

**Published:** 2021-05-06

**Authors:** Ali Pormohammad, Mohammad Zarei, Saied Ghorbani, Mehdi Mohammadi, Mohammad Hossein Razizadeh, Diana L. Turner, Raymond J. Turner

**Affiliations:** 1 Department of Biological Sciences, University of Calgary, Calgary, AB T2N 1N4, Canada; ali.pormohammad@ucalgary.ca (A.P.); mehdi.mohammadiashan@ucalgary.ca (M.M.); 2 John B. Little Center for Radiation Sciences, Harvard T.H. Chan School of Public Health, Boston, MA 02115, USA; mzarei@hsph.harvard.edu; 3 Department of Virology, Faculty of Medicine, Iran University of Medical Science, Tehran 1449614535, Iran; vet.s.ghorbani@gmail.com (S.G.); razizadeh.mh@iums.ac.ir (M.H.R.); 4 Department of Family Medicine, Cumming School of Medicine, University of Calgary, Calgary, AB T2N 4N1, Canada; dturner23@me.com

**Keywords:** COVID-19, SARS-CoV-2, vaccines, efficacy, side effect, randomized clinical trial, meta-analysis

## Abstract

The current study systematically reviewed, summarized and meta-analyzed the clinical features of the vaccines in clinical trials to provide a better estimate of their efficacy, side effects and immunogenicity. All relevant publications were systematically searched and collected from major databases up to 12 March 2021. A total of 25 RCTs (123 datasets), 58,889 cases that received the COVID-19 vaccine and 46,638 controls who received placebo were included in the meta-analysis. In total, mRNA-based and adenovirus-vectored COVID-19 vaccines had 94.6% (95% CI 0.936–0.954) and 80.2% (95% CI 0.56–0.93) efficacy in phase II/III RCTs, respectively. Efficacy of the adenovirus-vectored vaccine after the first (97.6%; 95% CI 0.939–0.997) and second (98.2%; 95% CI 0.980–0.984) doses was the highest against receptor-binding domain (RBD) antigen after 3 weeks of injections. The mRNA-based vaccines had the highest level of side effects reported except for diarrhea and arthralgia. Aluminum-adjuvanted vaccines had the lowest systemic and local side effects between vaccines’ adjuvant or without adjuvant, except for injection site redness. The adenovirus-vectored and mRNA-based vaccines for COVID-19 showed the highest efficacy after first and second doses, respectively. The mRNA-based vaccines had higher side effects. Remarkably few experienced extreme adverse effects and all stimulated robust immune responses.

## 1. Introduction

Severe acute respiratory syndrome coronavirus 2 (SARS-CoV-2) is a non-segmented positive-sense, single-stranded ribonucleic acid (RNA) beta coronavirus [1] that was first reported in Wuhan, China. The SARS-CoV-2 infection causes the coronavirus disease 2019 (COVID-19) that became a global pandemic and public health crisis. Over 140 million infected and 3 million deaths are reported from COVID-19 by April 2021, with the death rate accelerating; according to WHO, the case fatality ratio (CFR) of SARS-CoV-2 ranges from less than 0.1% to over 25% depending on the country [2]. 

To overcome this pandemic, vaccination is the hope for a safe and effective way to help build protection and reduce disease spread [3]. More than 200 COVID-19 vaccine candidates presented in different stages of development and over 50 candidates have reached clinical trials to date [4], including: Oxford-AstraZeneca’s ChAdOx1/AZD1222, Moderna’s mRNA-1273, Pfizer-BioNTech’s mRNA BNT162b2, Gamaleya’s Sputnik V, Johnson & Johnson’s INJ-7843735/Ad26.COV2.s, CoronaVac, Sinopharm’s BBIBP-CorV, Novavax’s NVX-CoV2373, EpiVacCorona, CanSino’s Convidicea (Ad5-nCoV), SinoVac’s CoronaVac, Anhui Zhifei Longcom’s ZF2001, GlaxoSmithKline and Medicago’s CoVLP, and Bharat Biotech’s BBV152/Covaxin.

Different strategies have been considered for the development of vaccines against SARS-CoV-2 based on the following vaccine platforms: (I) Nucleic acid mRNA-based vaccines are the newest generation of vaccine production approach [5]. The mRNA vaccine technology is a single-stranded RNA molecule that carries a portion of the coding sequence for the peptide or protein from the virus that can be synthesized in the cytoplasm (ribosomes). The resulting antigen triggers an immune response, including the production of antibodies [5]. For instance, the current vaccines developed by the companies Pfizer and Moderna utilize synthetic mRNA encoding the sequence of the coronavirus’s signature spike protein (S-protein) that is then encapsulated within a lipid vesicle nanoparticle. (II) Viral vector vaccines that are developed with new biotechnology [6]. A modified existing virus, able to infect human cells, is introduced carrying the genetic code of the target virus antigen in order to stimulate an immune response. Oxford-AstraZeneca, Gamaleya, CanSio and Johnson & Johnson developed their vaccines based on a DNA sequence encoding the S-protein inserted into the genome of a modified safe adenovirus. (III) Whole-Pathogen Inactivated virus vaccines consisting of killed/inactivated whole viruses or virus fragments. Here the pathogen’s genetic material is destroyed by heat, chemicals, or radiation, so that they cannot replicate but their presence can still stimulate immunogenicity [7]. Sinopharm, SinoVac, and Bharat Biotech’s vaccines were produced by inactivating the SARS-CoV-2 with B-propiolactone, but all the viral protein remains intact. (IV) Subunit vaccines that contain a fragment of the pathogen, either a protein (Pro-subunit), a polysaccharide, or a combination of both, without introducing viable pathogen particles [8]. Lack of genetic material makes them safe and non-infectious/non-viable. Novavax and Anhui Zhifei Longcom applied this technology for the development of their vaccine, using nanoparticles coated with synthetic S-protein and an adjuvant for boosting the immune response. Virus-like particle (VLP) vaccines, also a subunit vaccine, mimic the native virus structure, but contain no viral genetic material [9]. A VLP presents the antigen inserted on a nanoparticle surface. GlaxoSmithKline and Medicago used a plant-derived platform to produce a particle that elicits neutralizing antibody and immune cell (e.g., TH1 T cell) responses against COVID-19.

The structural proteins of SARS-CoV-2 include four major proteins: spike (S), membrane (M), and envelope (E) part of the viral surface envelope, and the nucleocapsid (N) protein in the ribonucleoprotien core. Among viral surface elements, the S-protein is a primary target for vaccines and therapeutic development against COVID-19 due to its role in the receptor recognition for cell entry and cell membrane fusion process. The trimeric S-protein contains two subunits, S1 and S2. The S1 contains a receptor-binding domain (RBD), which is responsible for recognizing and binding to the host receptor angiotensin-converting enzyme 2 (ACE2), while the S2 mediates the membrane fusion process by forming a six-helical bundle (6-HB) via the two-heptad repeat (HR) regions [10]. However, S1 is the immunodominant antigen during CoV infections and induces long-lasting and broad-spectrum neutralizing antibodies (NAbs) and T-cell immune responses against the RBD. Thus, the S-protein and the RBD serve as the promising targets of SARS-CoV-2 vaccines and the predominant antigenic target for developing a vaccine [11]. Other antigenic targets, such as non-structural proteins (nsp) 3, nsp8-10 [12], papain-like proteases (PLpro), and cysteine-like protease (3CLpro) [13] can be considered an alternative for vaccine development, but these are expected to elicit less of an immune response.

Efficacy and safety, thus side effect profiles, are core vaccine competencies required by medical care systems and public health. To the best of our knowledge, there is still no comprehensive comparative study around the efficacy and safety of COVID-19-related vaccines. In this regard, we provide here a meta-analysis on available randomized clinical trial (RCT) publications providing information on the efficacy and side effects of COVID-19 vaccines.

## 2. Methods

### 2.1. Search Strategy

The Preferred Reporting Items for Systematic Reviews and Meta-Analyses Statement (PRISMA) recommendations were followed in this analysis [14]. We searched all clinical trial publications related to SARS-CoV-2 vaccines from the following databases: Scopus, EMBASE, Medline (via PubMed), and Web of Science. All studies published up to 12 March 2021 were searched without language restriction by three independent reviewers. Search medical subject headings (MeSH) terms used were “Covid-19 Vaccine”, “SARS-CoV-2 Vaccine”, “clinical trial” or “phase trial”, and “randomized”, as well as all synonyms. We used the Center for Disease Control (CDC), World Health Organization (WHO), and Google Scholar databases/academic search engines to look for unpublished and grey literature. References and citation lists of selected articles and reviews were also reviewed for any other relevant literature (forward and backward citation, recommended by Cochrane). Additional search strategy details are provided in Table S1.

### 2.2. Study Selection

The records were first reviewed by three independent authors based on the title and abstract (MHR, AP, and SG), all unrelated publications were removed and the full texts of the remaining articles were reviewed. Then, two independent reviewers (AP and SG) judged potentially eligible articles and disagreements were resolved by discussion and for each article a consensus was reached.

### 2.3. Eligibility and Inclusion Criteria

The following predetermined conditions had to be met for studies to be considered for inclusion in this meta-analysis. For initial screening, all clinical studies were included in the systematic review, while RCT studies in phase I/II/III of COVID-19 vaccines were included in the meta-analysis. 

### 2.4. Exclusion Criteria

Non-randomized studies, studies without a placebo group, preclinical studies, studies on animal phase, meta-analyses, letters to the editor, review articles, studies with no extractable data, and news reports were excluded for the meta-analysis. However, non-randomized studies were included only in the systematic review. Additionally, 11 vaccine studies (43 datasets) with no report in the type of adjuvants were excluded from the adjuvant side effect sub-group meta-analysis.

### 2.5. Data Extraction

Four independent reviewers extracted data from the studies that were chosen. The following data were obtained from each article: first authors, trial initiation date, published year, vaccine name, company, study type, vaccine type, adjuvant, store temperature, trial phase, doses, injection interval (days), concentration, volume, trial country, all side effects, and efficacy-related data. Three of the authors (S.G, M.H.R, and A.P) extracted data independently, and another author (M.Z) reviewed extracted data at random; discrepancies were resolved by consensus. 

### 2.6. Quality Assessment

The JADAD scale (Oxford quality scoring system) for reporting quality of RCTs was used to evaluate the included articles’ quality. The JADAD scale included the three quality parameters of randomization, blinding, and account of all patients. Two questions are asked for the first two parameters, and one question is asked for the third parameter. Each query is given a score of one or zero. The highest acceptable score on the prepared checklist was five, with the lowest acceptable score being three. Data were derived from papers with a ranking of at least three (Table S2).

### 2.7. Analysis

Initially, cleaning data and preparing them for analysis was done in Microsoft Office 365 and analysis was performed by Comprehensive Meta-Analysis Software Version 2.0 software. The point estimates of the effect size, odds ratios (ORs), and 95% confidence interval (95% CI) were calculated for estimating vaccine efficacy and side effects. Random effects models were used to estimate pooled effects. Additionally, to search for heterogeneity between studies, the I2 statistic was used [15] and high heterogeneity was characterized as an I2 > 50%, with sources of heterogeneity established through meta-regression and subgroup analyses. Subgroup analysis based on the vaccine phases significantly decreased the heterogeneity in the high heterogeneity cases. The presence and effect of publication bias were examined using funnel plots, Begg’s test, and Egger weighted regression methods [16,17]. For all analyses, two-tailed statistics and a significance level of less than 0.05 were considered.

## 3. Result

### 3.1. Characteristics of Included Studies 

**Figure 1:**
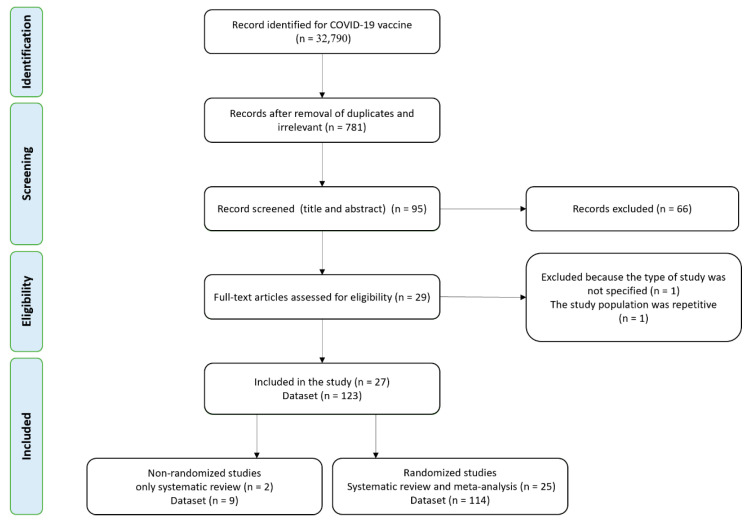
Flow diagram of literature search and study selection for meta-analysis (PRISMA flow chart).

A total of 32,790 publications were screened for the COVID-19 vaccines’ side effects and efficacies. Out of these studies, 27 met the systematic review’s inclusion criteria (non-randomized and randomized), while 25 randomized studies were included in the meta-analysis (Figure 1). Characteristics of the selected articles are summarized in Table 1. A total of 25 studies (123 datasets) were included in the meta-analysis. Studies with different vaccine phase reports, number of doses, injection concentration, different case, and control group numbers are considered a separate dataset for the meta-analysis. All included studies were written in English. Out of 25 randomized studies, 12 were double-blind, 2 participant-blind, 6 observer-blind, 3 single-blind, and 2 partially blind. The number of studies by vaccine platforms were 7 mRNA-based, 4 pro-subunit, 8 adenovirus-vector, 5 inactivated, and 1 VLPs.

### 3.2. Characteristics of Participants

A total of 58,889 cases that received the COVID-19 vaccine and 46,638 controls who received placebo were included in this study. Out of 58,889 vaccine cases, 31,070 were male and 27,819 female. Out of 46,638 individuals in the placebo group, 33,354 were male and 13,284 female. All vaccines and placebos were intramuscularly (IM) injected. Detailed information of age ranges of either vaccine or placebo groups is shown in Table 1.

### 3.3. Efficacy of Different COVID-19 Vaccines

#### 3.3.1. Efficacy of mRNA-Based COVID-19 Vaccines 

**Figure 2:**
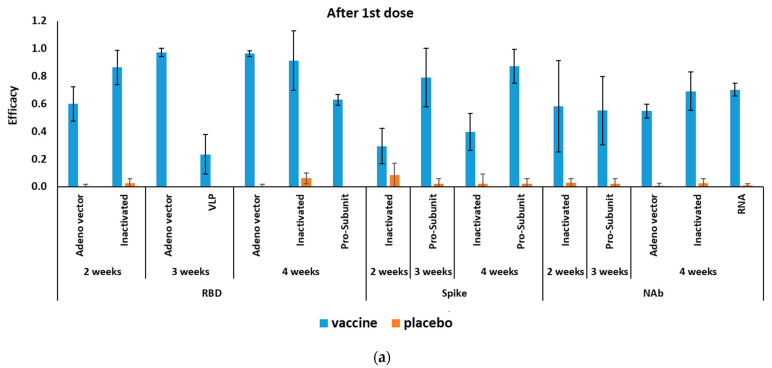

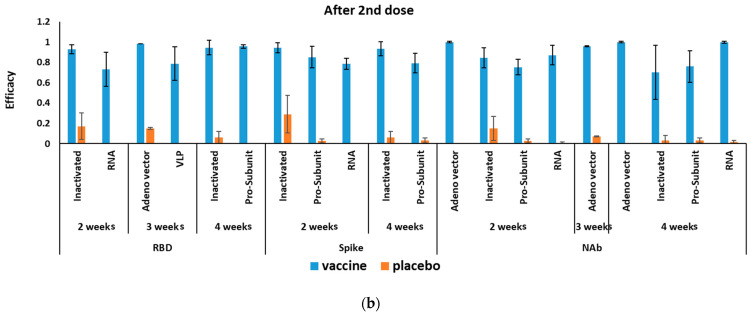
Efficacy of different COVID-19 vaccines (**a**) after the first and (**b**) second doses.

The mRNA-based COVID-19 vaccines had 94.6% (95% CI 0.936–0.954) efficacy in a total of 34,041 cases in phase II/III RCTs (Table 2). Figure 2 shows the efficacy of COVID-19 vaccines after the first and second doses. Efficacy four weeks after first dose was reported for only one antibody (NAb 70.2% (95% CI 0.655–0.746)). Efficacy after a second dose of mRNA-based COVID-19 vaccines was reported for RBD, S-protein, and NAbs, with the highest efficacy for NAbs at 99.5% (95% CI 0.980–0.999) (Table 3).

#### 3.3.2. Efficacy of Adenovirus-Vectored COVID-19 Vaccines

The pooling of four RCTs (in phases II/III) results (a total of 20,771 cases included) showed adenovirus-vectored COVID-19 vaccines had 80.2% (95% CI 0.56–0.93) efficacy (Table 2). After the first dose, the efficacy of the adenovirus-vectored COVID-19 vaccine was the highest at 97.6% (95% CI 0.939–0.997) against RBD three weeks after injection. Whereas, adenovirus-vectored COVID-19 vaccine had the highest efficacy by producing NAbs 99.9% (95% CI 0.985–1.000) after 4 and 2 weeks of the second injection (Table 3).

#### 3.3.3. Efficacy of Inactivated COVID-19 Vaccines

After the first vaccine dose, the inactivated COVID-19 vaccine’s efficacy was the highest against RBD at 91.3% (95% CI 00.564–0.96) four weeks after injection. Whereas, the highest efficacy against S-protein was 94% (95% CI 0.941 0.877–0.973) two weeks after the second injection (Table 3).

#### 3.3.4. Efficacy of Pro-Subunit COVID-19 Vaccines

Pro-subunit vaccine efficacy was the highest against RBD at 87.3% (95% CI 0.671–0.892) four weeks after the first dose. Similarly, it had the highest efficacy against RBD protein at 95.6% (95% CI 0.937–0.970) four weeks after the second dose (Table 3).

#### 3.3.5. Efficacy of VLP COVID-19 Vaccines

Efficacy of VLP vaccines was reported only against RBD three weeks after the first dose at 23.8% (95% CI 0.091–0.375) and three weeks after the second dose at 78.7% (95% CI 0.581–0.908) (Table 3). 

### 3.4. Side Effects of Different COVID-19 Vaccines

**Figure 3:**
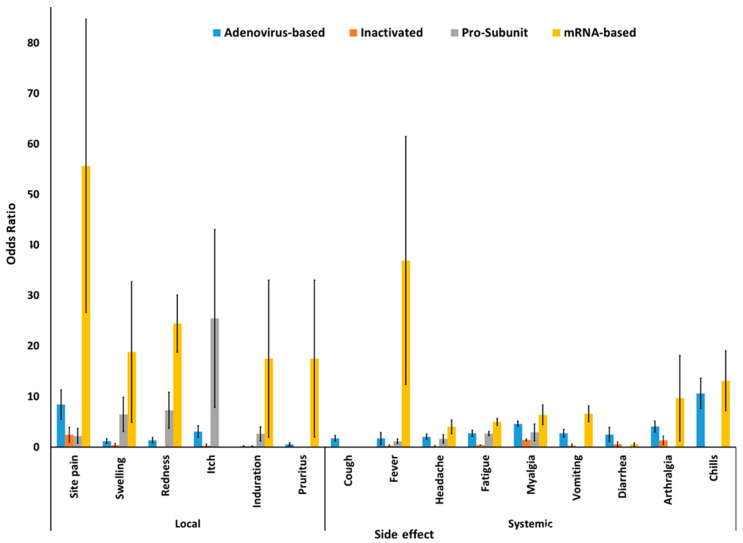
Local and systemic side effects of different COVID-19 vaccines.

Adjusted pooled odds ratio (OR) between vaccine and placebo groups were assessed for estimating the association of side effects by the administration of different COVID-19 vaccines. mRNA-based vaccines had the highest number of associated side effects, except for diarrhea and arthralgia, for which the adenovirus-vectored vaccine had the highest OR (Figure 3).

The administration of mRNA-based vaccine was associated with a greater number of side effects, such as injection site pain, fever, redness, swelling, induration, pruritus, chills, myalgia, arthralgia, vomiting, fatigue, and headache, by yielding a summary OR of 83.06 (95% CI 37.05–186.1) (in phase II/III RCTs), 36.90 (95% CI 12.34–105.21) (in phase I/II/III RCTs), 24.40 (95% CI 18.73–31.77) (in phase I/II/III RCTs), 18.79 (95% CI 4.87–72.40) (in phase I/II/III RCTs), 17.5 (95% CI 1.96–155.58) (in phase I/II RCTs), 17.50 (95% CI 1.98–155.58) (in phase II/III RCTs), 13.11 (95% CI 7.19–23.89) (in phase II/III RCTs), 10.71 (95% CI 6.51–17.60) (in phase I/II RCTs), 9.67 (95% CI 1.27–76.90) (in phase III/II RCTs), 8.71 (95% CI 4.38–17.34) (in phase I/II RCTs), 6.16 (95% CI 5.86–6.48) (in phase III RCTs), and 5.13 (95% CI 2.32–11.31) (in phase I/II RCTs), respectively, compared to other types of vaccines. Nevertheless, the adenovirus-vectored vaccine was associated with higher rates of diarrhea with OR of 4.59 (95% CI 3.58–5.89), and arthralgia OR of 10.61 (95% CI 7.60–14.83) compared to others (Table 4). It should be considered that heterogeneity (I-squared test) of the pooled meta-analysis for most of the side effects was low (I2 < 50%), which indicates that variation in study outcomes between the included studies was low, even though different companies and different research groups across the world have been included. More detailed information such as Forest plot, Funnel plot, heterogeneity test, and sub-group analysis of each side effects are shown in Figures S1–S21 (in the Supplementary Materials). 

#### Serious Adverse Side Effects of COVID-19 Vaccines

Only three studies reported anaphylactic shock as an adverse effect of COVID-19 vaccines—(1) 1 out of 84 vaccine cases for the inactivated vaccine [30]; (2) 1 case out of 2063 vaccinated for the adenovirus-based vaccine [38], (3) 1 case out of 15,181 in the vaccine group, and 1 case out of 15,170 in the placebo group, for the mRNA-based vaccine [40]. A total of 37 blot clots, including 22 pulmonary embolus cases (PE) and 5 deep vein thrombosis (DVT), have been reported for the Oxford-AstraZeneca vaccine among 17 million people in the EU and Britain [43]; see discussion for recent contributions. The number of clotting events is not greater than what is seen in the general population, with no indication that there is a causal effect. 

### 3.5. Side Effects of COVID-19 Vaccines Based on Different Adjuvants 

**Figure 4:**
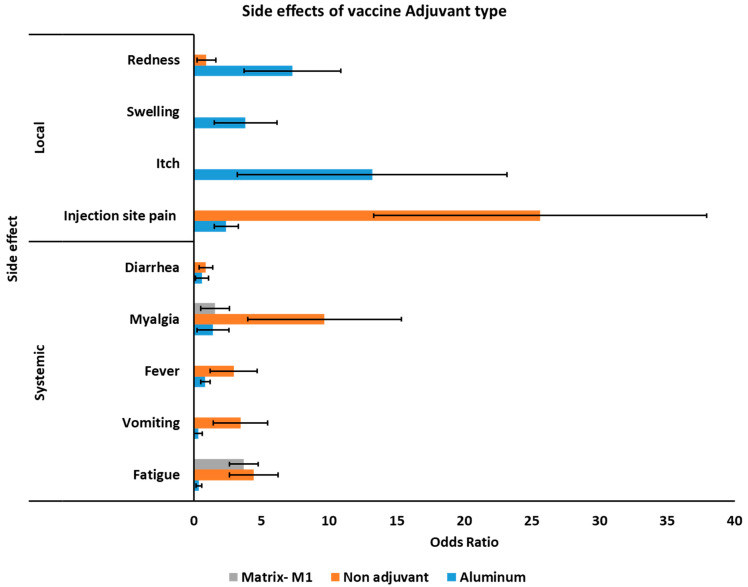
Association of adjuvant side effects of different COVID-19 vaccines.

The sub-group analysis was assessed to estimate the potential side effects of COVID-19 vaccines based on the different types of administrated adjuvants. Interestingly, in all cases, potassium aluminum sulfate (alum) had the smallest number of systemic and local side effects compared to other adjuvants or vaccines without adjuvant, except injection site redness, of which vaccines without adjuvant had higher rates of site redness (Figure 4). Accordingly, vaccines with alum adjuvant had lower systemic side effects of fatigue OR 0.392 (95% 0.18–0.82), vomiting 0.325 (95% 0.02–5.30), fever 0.85 (95% 0.51–1.43), myalgia 1.43 (95% 0.25–8.0), diarrhea 0.608 (95% 0.13–2.87), and injection site pain 2.40 (95% 1.51–3.83) between different adjuvants and vaccines with no adjuvant (Table 5). The vaccine with no adjuvant was associated with higher redness OR 0.923 (95% 0.23–3.6). Itch OR 13.20 (95% 3.23–53.90) and swelling OR of 3.83 (95% 1.52–9.64) was only reported for vaccines with alum adjuvant. For more detailed information see Figures S22–S30.

## 4. Discussion

The purpose of vaccination is to protect individuals from infection and transmission. Although the emergency use authorization for some of the COVID-19 vaccines has been approved by the Food and Drug Administration in the US and the Department of Health and Human Services of each country, the vaccines’ efficacy and side effects have not yet been comprehensively discussed, although popular media and politicians have made many unsubstantiated claims. Therefore, in the current meta-analysis, we provide systematic and comprehensive data regarding the vaccines’ safety, efficacy, and immunogenicity against SARS-CoV-2. Here, we mainly focused on available RCTs publications on the safety, efficacy, and immunogenicity of COVID-19 vaccines. 

The present study was carefully surveyed for the general and specific target antigen efficacy of each vaccine group. Our analysis showed that variation in the efficacy of vaccines after the first doses are remarkable in comparison with the efficacies after the second doses. Therefore, enrollment of the second dose should produce a more reliable outcome and efficacy compared to a single dose. In total, mRNA-based COVID-19 vaccines had 94.6% efficacy. The RNA-based vaccine elicited high levels of NAbs after one month of the first (70%) and second (99.5%) doses. Unfortunately, data for the RNA-based vaccines against RBD antigen were not available after the first dose. Protection against variants has been shown with the mRNA-based vaccine against the United Kingdom (B.1.1.7, also called 20I/501Y.V1) variant [44,45], but they may be less effective against the variant first detected in South Africa (B.1.351, known as 20H/501Y.V2) [46]. A week after the second dose of mRNA-based vaccine, induction of neutralizing antibody titers in the serum sample was 6-fold lower for participants bearing B.1.351 variant compared to original Wuhan-Hu-1 spike protein [47]. The B.1.351 variant carries two substitutions within the S-protein, which can escape three classes of therapeutically relevant antibodies. These data indicate reinfection with antigenically distinct variants and mitigates the full efficacy of spike-based COVID-19 vaccines [48]. 

From our summary analysis, the total efficacy of the adenovirus-vectored COVID-19 vaccines was 80.2%. The highest efficacy after a single dose is reported with the adenovirus-vectored COVID-19 vaccines, with very low variation and CI against RBD at 3 weeks (96.7%) and 4 weeks (96.6%) after vaccination compared to placebo controls. Some of the adenovirus-vectored COVID-19 vaccines, such as Johnson & Johnson, need just one dose, with the efficacy against RBD being a possible reason. However, based on the rollout timeline, long-term (more than four weeks) efficacy of adenovirus-vectored COVID-19 vaccines was not reported by any of the RCTs. For the other vaccine types, total efficacy has not been reported, only the antigen-specific efficacy was reported in these RCTs. The pro-subunit vaccine had the highest efficacy against spike antigen at 1 month after the first injection. The efficacy of the VLP vaccines was lower than other COVID-19 vaccines and reported only against RBD after the first (23.8%) and second dose (78.7%). All reports for VLP vaccines are from RCT phase I trials, and the lower efficacy of these vaccines may be the most probable reason. 

Any vaccine is expected to cause temporary side effects caused by activation of an immune response and injection site tissue trauma. Uptake of vaccines is related to perceived and real adverse side effects, both short-term and long-term. In this study, adjusted pooled odds ratios between vaccine and placebo groups indicated that RNA-based vaccines had higher rates of side effects in reactogenicity, including site pain, swelling, redness, fever, headache, fatigue, induration, vomiting, myalgia, chills, and pruritus (Table 2). No sign of cough or itch was found in RNA-based vaccines, and lower rates of diarrhea and arthralgia were observed for this vaccine. By avoiding negativity bias, this might provide strong evidence of RNA-based vaccines’ effectiveness, by eliciting a more robust immune response than other vaccine groups. Additionally, the rate of serious adverse side effects such as anaphylactic shock, an allergic reaction, was not remarkable with this vaccine, with only one case reported in both the vaccine and placebo groups [40]. 

In the context of side effects, the adenovirus-vectored vaccines are associated with increased diarrhea and arthralgia in comparison with other vaccines, see Table 2. A recent systematic review and meta-analysis by Yuan et al. [49] showed no significant difference in systemic reactions, with only local side effects, including pain, itching, and redness, being reported [49]. One case of anaphylactic shock was reported for this vaccine [38]. 

Several pulmonary emboli (PE) and deep vein thrombosis (DVT) cases have been reported as rare events for the Oxford-AstraZeneca vaccine, causing a temporary suspension of this vaccine’s use in many countries and age-specific rollout in others. However, to date, the data are too weak and anecdotal to provide clear evidence of cause and effect [50]. Similarly, the Johnson & Johnson vaccine was also temporarily suspended in April 2021 by the FDA, as several people developed rare blood-related problems of thrombosis with thrombocytopenia syndrome leading to cerebral venous sinus thrombosis (CVST) [51]. A DVT has also been reported shortly after the second dose of an mRNA-based vaccine as well [52]. Anaphylaxis as an acute allergic reaction has also been reported as a rare event for some vaccines, such as mRNA COVID-19 vaccines [53] and adenovector vaccines against COVID-19 [54]. Overall, these severe life-threatening adverse events are occurring rarely, thus supporting the ongoing rollout of global vaccination programs.

Data are currently emerging on Vaccine-induced Immune Thrombotic Thrombocytopenia (VITT) following vaccination with COVID-19 vaccines [55]. VITT presents with symptoms of thromboembolism and especially signs of thrombocytopenia, cerebral blood clots, or abdominal or arterial clots, such as easy bruising, bleeding or new and/or severe headaches, and pain in the abdomen or a painful, cold numb extremity, particularly with onset 4 to 28 days after immunization. This is due to thrombosis (blood clots) involving the cerebral venous sinuses, or CVST (large blood vessels in the brain), and other sites in the body (including but not limited to the large blood vessels of the abdomen and the veins of the legs) along with thrombocytopenia, or low blood platelet counts. These events are rare, but to date have been documented for the mRNA vaccines BNT162b2 (Pfizer-BioNTech) and mRNA-1273 (Moderna) and the adenoviral vector vaccines ChAdOx1 nCoV-19 vaccine (Astra Zeneca) and Ad26. COV2-S vaccine (Janssen; Johnson & Johnson). Given the very recent emergence, our meta study does not include an analysis of VITT.

Co-administration of vaccine with adjuvants is being used in VLP subunit vaccines and certain inactivated vaccines [55]. Adjuvants have an essential role owning to inducing specific immune responses, IgG_1_, and NAbs titers. It also considers potential dose-sparing of CoV vaccine [56]. Multiple adjuvants, such as alum salts, emulsions, and TLR agonists have been formulated for SARS-CoV, SARS-CoV-2, and MERS-CoV [55]. The potential side effects of COVID-19 vaccines based on the different types of adjuvants investigated showed that alum-adjuvanted CoV vaccines had the lowest systemic side effects among other adjuvants or non-adjuvant in Table 3. The non-adjuvanted vaccines revealed immunopathologic reactions including high fatigue, vomiting, fever, myalgia, and diarrhea and redness, while alum-adjuvanted CoV vaccines showed itch and swelling. Overall, the metadata obtained in this study demonstrated that the alum-adjuvanted CoV vaccines had the smallest number of issues compared with other adjuvants and the non-adjuvant formulations. 

The limitations of this study are: 1. The overall effectiveness and antigen-specific efficacy of some vaccines have not been reported after the first or second dose. 2. Some trials had considerable bias by not including a sufficient number of samples or a broad enough geographical, economic, and age diversity. 3. Timing of vaccine trials in relation to overall prevalence through the COVID-19 pandemic impacts direct comparison. 4. The IgG and IgM antibodies in serum levels had a wide range of variation across the different vaccines after the first or second dose, thus, these data were not included in the meta-analysis. 5. The lack of data on specific categories of patients such as pregnant patients and lifestyles. 6. All RCTs followed up the vaccine and placebo groups one month after both first and second doses, therefore, all reports are related to short-term impacts of the vaccine. 7. For the prevention of database bias, we searched various databases and websites for finding all relevant and gray publications and a proper test for publication bias using Egger’s regression test conducted. We did not find remarkable publication bias in this study by Egger’s regression test. However, publication bias and heterogeneity for some of the pooled results, as well as all the above limitations, must be considered when interpreting the outcomes.

## 5. Conclusions

The adenovirus-vectored and mRNA-based vaccines for COVID-19 showed the highest efficacy after first and second doses, respectively. The mRNA-based vaccines had higher side effects. Only a rare few recipients have experienced extreme adverse effects and all stimulated robust immune responses. All RCTs followed up the vaccine and placebo groups after one month after both first and second doses, therefore, all reports are related to short-term impacts. Due to the timeline, all the vaccines are missing longer-term assessments. This meta-analysis allows us to incorporate relevant new evidence for summarizing and analyzing the clinical features of current vaccines for COVID-19 in phase I, II, and III RCTs. The results support the overall efficacy and safety of all available COVID-19 vaccines, providing clear data-driven evidence to support the ongoing global public health effort to vaccinate the entire population.

## Figures and Tables

**Table 1:** Characterization of included studies.

Study	Trial Initiation Date	Pub. Year	Vaccine Name	Company	Study Type	Vaccine Type	Adjuvant	Store Temp (°C)	RCT Phase	Dose (s)	Age Range (Year)	Injection Interval (Days)	Concentration	Trial Country	Ref
Yang et al.	22 June and 3 July 2020	2020	ZF2001	Anhui Zhifei Longcom	Randomized, double-blind, placebo-controlled	Pro-Subunit	Aluminum hydroxide	2–8	I	3	22.9–54.7	30	25 μg *	China	[18]
Yang et al.	22 June and 3 July 2020	2020	ZF2001	Anhui Zhifei Longcom	Randomized, double-blind, placebo-controlled	Pro-Subunit	Aluminum hydroxide	2–8	I	3	20.9–49.4	30	50 μg *	China	[18]
Ella et al.	13 and 30 July 2020	2021	BBV152 (Covaxin)	Bharat Biotech	Randomized, double-blind, placebo-controlled	Inactivated	Algel-IMDG	2–8	I	2	18–55	14	3 μg *	India	[19]
Ella et al.	13 and 30 July 2020	2021	BBV152 (Covaxin)	Bharat Biotech	Randomized, double-blind, placebo-controlled	Inactivated	Algel-IMDG	2–8	I	2	18–55	14	6 μg *	India	[19]
Ella et al.	13 and 30 July 2020	2021	BBV152 (Covaxin)	Bharat Biotech	Randomized, double-blind, placebo-controlled	Inactivated	Algel	2–8	I	2	18–55	14	6 μg*	India	[19]
Zhu et al.	16 and 27 March 2020	2020	Ad5-nCoV	CanSino	Non-randomized	Adenovirus-based	No adjuvant	UN	I	1	18–60	No	5 × 10^1^⁰ VP *	China	[20]
Zhu et al.	16 and 27 March 2020	2020	Ad5-nCoV	CanSino	Non-randomized	Adenovirus-based	No adjuvant	UN	I	1	18–60	No	1 × 10^11^ VP *	China	[20]
Zhu et al.	16 and 27 March 2020	2020	Ad5-nCoV	CanSino	Non-randomized	Adenovirus-based	No adjuvant	UN	I	1	18–60	No	1.5 × 10^11^ VP *	China	[20]
Richmond et al.	19 June and 23 September 2020	2021	SCB-2019	Clover	Randomized, double-blind, placebo-controlled	Pro-Subunit	No adjuvant	2–8	I	2	20–50	21	3 μg *	Australia	[21]
Richmond et al.	19 June and 23 September 2020	2021	SCB-2019	Clover	Randomized, double-blind, placebo-controlled	Pro-Subunit	AS03	2–8	I	2	24–53	21	3 μg *	Australia	[21]
Richmond et al.	19 June and 23 September 2020	2021	SCB-2019	Clover	Randomized, double-blind, placebo-controlled	Pro-Subunit	AS03	2–8	I	2	55–70	21	3 μg *	Australia	[21]
Richmond et al.	19 June and 23 September 2020	2021	SCB-2019	Clover	Randomized, double-blind, placebo-controlled	Pro-Subunit	CpG/Alum	2–8	I	2	20–53	21	3 μg *	Australia	[21]
Richmond et al.	19 June and 23 September 2020	2021	SCB-2019	Clover	Randomized, double-blind, placebo-controlled	Pro-Subunit	CpG/Alum	2–8	I	2	55–71	21	3 μg *	Australia	[21]
Richmond et al.	19 June and 23 September 2020	2021	SCB-2019	Clover	Randomized, double-blind, placebo-controlled	Pro-Subunit	No adjuvant	2–8	I	2	20–54	21	9 μg *	Australia	[21]
Richmond et al.	19 June and 23 September 2020	2021	SCB-2019	Clover	Randomized, double-blind, placebo-controlled	Pro-Subunit	AS03	2–8	I	2	21–53	21	9 μg *	Australia	[21]
Richmond et al.	19 June and 23 September 2020	2021	SCB-2019	Clover	Randomized, double-blind, placebo-controlled	Pro-Subunit	AS03	2–8	I	2	55–64	21	9 μg *	Australia	[21]
Richmond et al.	19 June and 23 September 2020	2021	SCB-2019	Clover	Randomized, double-blind, placebo-controlled	Pro-Subunit	CpG/Alum	2–8	I	2	19–55	21	9 μg *	Australia	[21]
Richmond et al.	19 June and 23 September 2020	2021	SCB-2019	Clover	Randomized, double-blind, placebo-controlled	Pro-Subunit	CpG/Alum	2–8	I	2	55–67	21	9 μg *	Australia	[21]
Richmond et al.	19 June and 23 September 2020	2021	SCB-2019	Clover	Randomized, double-blind, placebo-controlled	Pro-Subunit	No adjuvant	2–8	I	2	18–49	21	30 μg *	Australia	[21]
Richmond et al.	19 June and 23 September 2020	2021	SCB-2019	Clover	Randomized, double-blind, placebo-controlled	Pro-Subunit	AS03	2–8	I	2	19–47	21	30 μg *	Australia	[21]
Richmond et al.	19 June and 23 September 2020	2021	SCB-2019	Clover	Randomized, double-blind, placebo-controlled	Pro-Subunit	AS03	2–8	I	2	55–63	21	30 μg *	Australia	[21]
Richmond et al.	19 June and 23 September 2020	2021	SCB-2019	Clover	Randomized, double-blind, placebo-controlled	Pro-Subunit	CpG/Alum	2–8	I	2	21–50	21	30 μg *	Australia	[21]
Richmond et al.	19 June and 23 September 2020	2021	SCB-2019	Clover	Randomized, double-blind, placebo-controlled	Pro-Subunit	CpG/Alum	2–8	I	2	55–74	21	30 μg *	Australia	[21]
Kremsner et al.	June, 2020	2020	CVnCoV	Curevac	Randomized, partially blind, placebo-controlled	mRNA-based	No adjuvant	5	I	2	18–60	28	2 μg *	Germany	[22]
Kremsner et al.	June, 2020	2020	CVnCoV	Curevac	Randomized, partially blind, placebo-controlled	mRNA-based	No adjuvant	5	I	2	19–59	28	4 μg *	Germany	[22]
Kremsner et al.	June, 2020	2020	CVnCoV	Curevac	Randomized, partially blind, placebo-controlled	mRNA-based	No adjuvant	5	I	2	20–59	28	6 μg *	Germany	[22]
Kremsner et al.	June, 2020	2020	CVnCoV	Curevac	Randomized, partially blind, placebo-controlled	mRNA-based	No adjuvant	5	I	2	20–59	28	8 μg *	Germany	[22]
Kremsner et al.	June, 2020	2020	CVnCoV	Curevac	Randomized, partially blind, placebo-controlled	mRNA-based	No adjuvant	5	I	2	19–59	28	12 μg *	Germany	[22]
Ward et al.	July, 2020	2020	CoVLP	Medicago	Randomized, partially blind	VLP	No adjuvant	2–8	I	2	18–55	21	3.75 μg *	Canada	[23]
Ward et al.	July, 2020	2020	CoVLP	Medicago	Randomized, partially blind	VLP	CpG 1018	2–8	I	2	18–55	21	3.75 μg *	Canada	[23]
Ward et al.	July, 2020	2020	CoVLP	Medicago	Randomized, partially blind	VLP	AS03	2–8	I	2	18–55	21	3.75 μg *	Canada	[23]
Ward et al.	July, 2020	2020	CoVLP	Medicago	Randomized, partially blind	VLP	No adjuvant	2–8	I	2	18–55	21	7.5 μg *	Canada	[23]
Ward et al.	July, 2020	2020	CoVLP	Medicago	Randomized, partially blind	VLP	CpG 1018	2–8	I	2	18–55	21	7.5 μg *	Canada	[23]
Ward et al.	July, 2020	2020	CoVLP	Medicago	Randomized, partially blind	VLP	AS03	2–8	I	2	18–55	21	7.5 μg *	Canada	[23]
Ward et al.	July, 2020	2020	CoVLP	Medicago	Randomized, partially blind	VLP	No adjuvant	2–8	I	2	18–55	21	15 μg *	Canada	[23]
Ward et al.	July, 2020	2020	CoVLP	Medicago	Randomized, partially blind	VLP	CpG 1018	2–8	I	2	18–55	21	15 μg *	Canada	[23]
Ward et al.	July, 2020	2020	CoVLP	Medicago	Randomized, partially blind	VLP	AS03	2–8	I	2	18–55	21	15 μg	Canada	[23]
Jackson et al.	16 March and 14 April 2020	2020	mRNA-1273	Moderna	Open-label	mRNA-based	No adjuvant	−20	I	2	18–55	28	25 μg *	United States	[24]
Jackson et al.	16 March and 14 April 2020	2020	mRNA-1273	Moderna	Open-label	mRNA-based	No adjuvant	−20	I	2	18–55	28	100 mg *	United States	[24]
Jackson et al.	16 March and 14 April 2020	2020	mRNA-1273	Moderna	Open-label	mRNA-based	No adjuvant	−20	I	2	18–55	28	250 mg *	United States	[24]
Anderson et al.	16 April and 12 May 2020	2020	mRNA-1273	Moderna	Open-label	mRNA-based	No adjuvant	−20	I	2	56–70	28	25 mg *	United States	[25]
Anderson et al.	16 April and 12 May 2020	2020	mRNA-1273	Moderna	Open-label	mRNA-based	No adjuvant	−20	I	2	71≤	28	25 mg *	United States	[25]
Anderson et al.	16 April and 12 May 2020	2020	mRNA-1273	Moderna	Open-label	mRNA-based	No adjuvant	−20	I	2	56–70	28	100 mg *	United States	[25]
Anderson et al.	16 April and 12 May 2020	2020	mRNA-1273	Moderna	Open-label	mRNA-based	No adjuvant	−20	I	2	71≤	28	100 mg *	United States	[25]
Keech et al.	26 May and 6 June 2020	2020	NVX-CoV2373	Novavax	Randomized, observer-blind, placebo-controlled	Pro-Subunit	No adjuvant	2–8	I	2	18–59	21	25 μg/0.6 ml	Australia, United States	[26]
Keech et al.	26 May and 6 June 2020	2020	NVX-CoV2373	Novavax	Randomized, observer-blind, placebo-controlled	Pro-Subunit	Matrix-M1	2–8	I	2	18–59	21	5 μg/0.6 ml	Australia, United States	[26]
Keech et al.	26 May and 6 June 2020	2020	NVX-CoV2373	Novavax	Randomized, observer-blind, placebo-controlled	Pro-Subunit	Matrix-M1	2–8	I	2	18–59	21	25 μg/0.6 ml	Australia, United States	[26]
Keech et al.	26 May and 6 June 2020	2020	NVX-CoV2373	Novavax	Randomized, observer-blind, placebo-controlled	Pro-Subunit	Matrix-M1	2–8	I	1	18–59	21	25 μg/0.6 ml	Australia, United States	[26]
Sahin et al.	23 April and 22 May 2020	2020	BNT162b1	Pfizer/BioNTech	Randomized, single-blind	mRNA-based	No adjuvant	(−60)–(−80)	I	2	18–55	21	1 μg *	Germany	[27]
Sahin et al.	23 April and 22 May 2020	2020	BNT162b1	Pfizer/BioNTech	Randomized, single-blind	mRNA-based	No adjuvant	(−60)–(−80)	I	2	21.4–55.8	21	10 μg *	Germany	[27]
Sahin et al.	23 April and 22 May 2020	2020	BNT162b1	Pfizer/BioNTech	Randomized, single-blind	mRNA-based	No adjuvant	(−60)–(−80)	I	2	25.1–55	21	30 μg *	Germany	[27]
Sahin et al.	23 April and 22 May 2020	2020	BNT162b1	Pfizer/BioNTech	Randomized, single-blind	mRNA-based	No adjuvant	(−60)–(−80)	I	2	23.9–54	21	50 μg *	Germany	[27]
Sahin et al.	23 April and 22 May 2020	2020	BNT162b1	Pfizer/BioNTech	Randomized, single-blind	mRNA-based	No adjuvant	(−60)–(−80)	I	1	19.9–47.8	No	60 μg *	Germany	[27]
Walsh et al.	4 May and 22 June 2020	2020	BNT162b1	Pfizer/BioNTech	Randomized, observer-blind, placebo-controlled	mRNA-based	No adjuvant	(−60)–(−80)	I	2	20.9–53.2	21	10 μg *	United States, Germany	[28]
Walsh et al.	4 May and 22 June 2020	2020	BNT162b1	Pfizer/BioNTech	Randomized, observer-blind, placebo-controlled	mRNA-based	No adjuvant	(−60)–(−80)	I	2	18–55	21	10 μg *	United States, Germany	[28]
Walsh et al.	4 May and 22 June 2020	2020	BNT162b1	Pfizer/BioNTech	Randomized, observer-blind, placebo-controlled	mRNA-based	No adjuvant	(−60)–(−80)	I	2	65–85	21	20 μg *	United States, Germany	[28]
Walsh et al.	4 May and 22 June 2020	2020	BNT162b1	Pfizer/BioNTech	Randomized, observer-blind, placebo-controlled	mRNA-based	No adjuvant	(−60)–(−80)	I	2	18–55	21	20 μg *	United States, Germany	[28]
Walsh et al.	4 May and 22 June 2020	2020	BNT162b1	Pfizer/BioNTech	Randomized, observer-blind, placebo-controlled	mRNA-based	No adjuvant	(−60)–(−80)	I	2	65–85	21	30 μg *	United States, Germany	[28]
Walsh et al.	4 May and 22 June 2020	2020	BNT162b1	Pfizer/BioNTech	Randomized, observer-blind, placebo-controlled	mRNA-based	No adjuvant	(−60)–(−80)	1	2	18–55	21	30 μg *	United States, Germany	[28]
Walsh et al.	4 May and 22 June 2020	2020	BNT162b2	Pfizer/BioNTech	Randomized, observer-blind, placebo-controlled	mRNA-based	No adjuvant	(−60)–(−80)	I	2	65–85	21	10 μg *	United States, Germany	[28]
Walsh et al.	4 May and 22 June 2020	2020	BNT162b2	Pfizer/BioNTech	Randomized, observer-blind, placebo-controlled	mRNA-based	No adjuvant	(−60)–(−80)	I	2	18–55	21	10 μg *	United States, Germany	[28]
Walsh et al.	4 May and 22 June 2020	2020	BNT162b2	Pfizer/BioNTech	Randomized, observer-blind, placebo-controlled	mRNA-based	No adjuvant	(−60)–(−80)	I	2	65–85	21	20 μg *	United States, Germany	[28]
Walsh et al.	4 May and 22 June 2020	2020	BNT162b2	Pfizer/BioNTech	Randomized, observer-blind, placebo-controlled	mRNA-based	No adjuvant	(−60)–(−80)	I	2	18–55	21	20 μg *	United States, Germany	[28]
Walsh et al.	4 May and 22 June 2020	2020	BNT162b2	Pfizer/BioNTech	Randomized, observer-blind, placebo-controlled	mRNA-based	No adjuvant	(−60)–(−80)	I	2	65–85	21	30 μg *	United States, Germany	[28]
Walsh et al.	4 May and 22 June 2020	2020	BNT162b2	Pfizer/BioNTech	Randomized, observer-blind, placebo-controlled	mRNA-based	No adjuvant	(−60)–(−80)	I	2	18–55	21	30 μg *	United States, Germany	[28]
Xia et al.	12 April and 2 May 2020	2020	BBIBP-CorV	Sinopharm	Randomized, double-blind, placebo controlled	Inactivated	Aluminum hydroxide	2–8	I	3	65–85	28	2.5 μg *	China	[29]
Xia et al.	12 April and 2 May 2020	2020	BBIBP-CorV	Sinopharm	Randomized, double-blind, placebo controlled	Inactivated	Aluminum hydroxide	2–8	I	3	18–59	28	5 μg *	China	[29]
Xia et al.	12 April and 2 May 2020	2020	BBIBP-CorV	Sinopharm	Randomized, double-blind, placebo controlled	Inactivated	Aluminum hydroxide	2–8	I	3	18–59	28	10 μg *	China	[29]
Xia et al.	29 April and 28 June 2020	2020	BBIBP-CorV	Sinopharm	Randomized, double-blind, placebo controlled	Inactivated	Aluminum hydroxide	2–8	I	2	18–59	28	2 μg *	China	[30]
Xia et al.	29 April and 28 June 2020	2020	BBIBP-CorV	Sinopharm	Randomized, double-blind, placebo controlled	Inactivated	Aluminum hydroxide	2–8	I	2	18–59	28	2 μg *	China	[30]
Xia et al.	29 April and 28 June 2020	2020	BBIBP-CorV	Sinopharm	Randomized, double-blind, placebo controlled	Inactivated	Aluminum hydroxide	2–8	I	2	60 ≤	28	4 μg *	China	[30]
Xia et al.	29 April and 28 June 2020	2020	BBIBP-CorV	Sinopharm	Randomized, double-blind, placebo controlled	Inactivated	Aluminum hydroxide	2–8	I	2	18–59	28	4 μg *	China	[30]
Xia et al.	29 April and 28 June 2020	2020	BBIBP-CorV	Sinopharm	Randomized, double-blind, placebo controlled	Inactivated	Aluminum hydroxide	2–8	I	2	60 ≤	28	8 μg *	China	[30]
Xia et al.	29 April and 28 June 2020	2020	BBIBP-CorV	Sinopharm	Randomized, double-blind, placebo controlled	Inactivated	Aluminum hydroxide	2–8	I	2	18–59	28	8 μg *	China	[30]
Zhang et al.	16 and 25 April 2020	2020	CoronaVac	Sinovac	Randomized, double-blind, placebo controlled	Inactivated	Aluminum hydroxide	2–8	I	2	60≤	14	3 μg *	China	[31]
Zhang et al.	16 and 25 April 2020	2020	CoronaVac	Sinovac	Randomized, double-blind, placebo controlled	Inactivated	Aluminum hydroxide	2–8	I	2	18–59	28	3 μg *	China	[31]
Zhang et al.	16 and 25 April 2020	2020	CoronaVac	Sinovac	Randomized, double-blind, placebo controlled	Inactivated	Aluminum hydroxide	2–8	I	2	18–59	14	6 μg *	China	[31]
Zhang et al.	16 and 25 April 2020	2020	CoronaVac	Sinovac	Randomized, double-blind, placebo controlled	Inactivated	Aluminum hydroxide	2–8	I	2	18–59	28	6 μg *	China	[31]
Logunov et al.	18 June and 3 August 2020	2020	Sputnik V	Gamaleya	Non-randomized	Adenovirus-based	No adjuvant	2–8	I	1	18–59	No	10^11^ VP *	Russia	[32]
Logunov et al.	18 June and 3 August 2020	2020	Sputnik V	Gamaleya	Non-randomized	Adenovirus-based	No adjuvant	2–8	I	1	18–60	No	10^11^ *	Russia	[32]
Logunov et al.	18 June and 3 August 2020	2020	Sputnik V (Lyo)	Gamaleya	Non-randomized	Adenovirus-based	No adjuvant	2–8	I	1	18–60	No	10^11^ *	Russia	[32]
Logunov et al.	18 June and 3 August 2020	2020	Sputnik V (Lyo)	Gamaleya	Non-randomized	Adenovirus-based	No adjuvant	2–8	I	1	18–60	No	10^11^ *	Russia	[32]
Folegatti et al.	23 April and 21 May 2020	2020	AZD1222	Oxford/AstraZeneca	Randomized, participant-blind, placebo-controlled	Adenovirus-based	No adjuvant	2–8	I/II	2	18–60	28	5 × 10^1^⁰ VP *	United Kingdom	[33]
Mulligan et al.	4 May and 19 June 2020	2020	BNT162b1	Pfizer/BioNTech	Randomized, observer-blind, placebo-controlled	mRNA-based	No adjuvant	(−60)–(−80)	I/II	2	18–55	21	10 μg *	Multicenter ^1^	[34]
Mulligan et al.	4 May and 19 June,2020	2020	BNT162b1	Pfizer/BioNTech	Randomized, observer-blind, placebo-controlled	mRNA-based	No adjuvant	(−60)–(−80)	I/II	2	24–42	21	30 μg *	Multicenter ^1^	[34]
Mulligan et al.	4 May and 19 June 2020	2020	BNT162b1	Pfizer/BioNTech	Randomized, observer-blind, placebo-controlled	mRNA-based	No adjuvant	(−60)–(−80)	I/II	1	23–52	No	100 μg *	Multicenter ^1^	[34]
Yang et al.	12 and 17 July 2020	2020	ZF2001	Anhui Zhifei Longcom	Randomized, double-blind, placebo-controlled	Pro-Subunit	Aluminum hydroxide	2–8	II	2	25–53	30	25 μg *	China	[18]
Yang et al.	12 and 17 July 2021	2020	ZF2001	Anhui Zhifei Longcom	Randomized, double-blind, placebo-controlled	Pro-Subunit	Aluminum hydroxide	2–8	II	2	18.8–58.4	30	50 μg *	China	[18]
Yang et al.	12 and 17 July 2022	2020	ZF2001	Anhui Zhifei Longcom	Randomized, double-blind, placebo-controlled	Pro-Subunit	Aluminum hydroxide	2–8	II	3	19.9–59.1	30	25 μg *	China	[18]
Yang et al.	12 and 17 July 2023	2020	ZF2001	Anhui Zhifei Longcom	Randomized, double-blind, placebo-controlled	Pro-Subunit	Aluminum hydroxide	2–8	II	3	20–59.7	30	50 μg *	China	[18]
Zhu et al.	11 and 16 April 2020	2020	Ad5-nCoV	CanSino	Randomized, double-blind, placebo-controlled	Adenovirus-based	No adjuvant	UN	II	1	19.3–59.6	No	1 × 10^11^ *	China	[35]
Zhu et al.	11 and 16 April 2020	2020	Ad5-nCoV	CanSino	Randomized, double-blind, placebo-controlled	Adenovirus-based	No adjuvant	UN	II	1	18≤	No	5 × 10^1^⁰ *	China	[35]
Chu et al.	29 May and 8 July 2020	2020	mRNA-1273	Moderna	Randomized, observer-blind, placebo-controlled	mRNA-based	No adjuvant	−20	II	2	18≤	28	50 mg *	United States	[36]
Chu et al.	29 May and 8 July 2020	2020	mRNA-1273	Moderna	Randomized, observer-blind, placebo-controlled	mRNA-based	No adjuvant	−20	II	2	18–54.99	28	50 mg *	United States	[36]
Chu et al.	29 May and 8 July 2020	2020	mRNA-1273	Moderna	Randomized, observer-blind, placebo-controlled	mRNA-based	No adjuvant	−20	II	2	55≤	28	100 mg *	United States	[36]
Chu et al.	29 May and 8 July 2020	2020	mRNA-1273	Moderna	Randomized, observer-blind, placebo-controlled	mRNA-based	No adjuvant	−20	II	2	18–54.99	28	100 mg *	United States	[36]
Ramasamy et al.	30 May and 8 August 2020	2020	AZD1222	Oxford/AstraZeneca	Randomized, participant-blind, placebo-controlled	Adenovirus-based	No adjuvant	2–8	II	2	55≤	28	2.2 × 10^1^⁰ VP *	United Kingdom	[37]
Ramasamy et al.	30 May and 8 August 2020	2020	AZD1222	Oxford/AstraZeneca	Randomized, participant-blind, placebo-controlled	Adenovirus-based	No adjuvant	2–8	II	1	18–55	No	2.2 × 10^1^⁰ VP *	United Kingdom	[37]
Ramasamy et al.	30 May and 8 August 2020	2020	AZD1222	Oxford/AstraZeneca	Randomized, participant-blind, placebo-controlled	Adenovirus-based	No adjuvant	2–8	II	2	56–69	28	2.2 × 10^1^⁰ VP *	United Kingdom	[37]
Ramasamy et al.	30 May and 8 August 2020	2020	AZD1222	Oxford/AstraZeneca	Randomized, participant-blind, placebo-controlled	Adenovirus-based	No adjuvant	2–8	II	1	56–69	No	2.2 × 10^1^⁰ VP *	United Kingdom	[37]
Ramasamy et al.	30 May and 8 August 2020	2020	AZD1222	Oxford/AstraZeneca	Randomized, participant-blind, placebo-controlled	Adenovirus-based	No adjuvant	2–8	II	2	70≤	28	2.2 × 10^1^⁰ VP *	United Kingdom	[37]
Ramasamy et al.	30 May and 8 August 2020	2020	AZD1222	Oxford/AstraZeneca	Randomized, participant-blind, placebo-controlled	Adenovirus-based	No adjuvant	2–8	II	2	70≤	28	3.5–6.5 × 10^1^⁰ VP *	United Kingdom	[37]
Ramasamy et al.	30 May and 8 August 2020	2020	AZD1222	Oxford/AstraZeneca	Randomized, participant-blind, placebo-controlled	Adenovirus-based	No adjuvant	2–8	II	1	18–55	No	3.5–6.5 × 10^1^⁰ VP *	United Kingdom	[37]
Ramasamy et al.	30 May and 8 August 2020	2020	AZD1222	Oxford/AstraZeneca	Randomized, participant-blind, placebo-controlled	Adenovirus-based	No adjuvant	2–8	II	2	56–69	28	3.5–6.5 × 10^1^⁰ VP *	United Kingdom	[37]
Ramasamy et al.	30 May and 8 August 2020	2020	AZD1222	Oxford/AstraZeneca	Randomized, participant-blind, placebo-controlled	Adenovirus-based	No adjuvant	2–8	II	1	56–69	No	3.5–6.5 ×10^1^⁰ VP *	United Kingdom	[37]
Ramasamy et al.	30 May and 8 August 2020	2020	AZD1222	Oxford/AstraZeneca	Randomized, participant-blind, placebo-controlled	Adenovirus-based	No adjuvant	2–8	II	2	70≤	28	3.5–6.5 × 10^1^⁰ VP *	United Kingdom	[37]
Xia et al.	12 April and 2 May 2020	2020	BBIBP-CorV	Sinopharm	Randomized, double-blind, placebo controlled	Inactivated	Aluminum hydroxide	2–8	II	3	70≤	28	5 μg *	China	[29]
Xia et al.	12 April and 2 May 2020	2020	BBIBP-CorV	Sinopharm	Randomized, double-blind, placebo controlled	Inactivated	Aluminum hydroxide	2–8	II	3	18–59	28	5 μg *	China	[29]
Xia et al.	18 May and 30 July 2020	2020	BBIBP-CorV	Sinopharm	Randomized, double-blind, placebo controlled	Inactivated	Aluminum hydroxide	2–8	II	1	18–59	No	8 μg *	China	[30]
Xia et al.	18 May and 30 July 2020	2020	BBIBP-CorV	Sinopharm	Randomized, double-blind, placebo controlled	Inactivated	Aluminum hydroxide	2–8	II	2	18–59	14	4 μg *	China	[30]
Xia et al.	18 May and 30 July 2020	2020	BBIBP-CorV	Sinopharm	Randomized, double-blind, placebo controlled	Inactivated	Aluminum hydroxide	2–8	II	2	18–59	21	4 μg *	China	[30]
Xia et al.	18 May and 30 July 2020	2020	BBIBP-CorV	Sinopharm	Randomized, double-blind, placebo controlled	Inactivated	Aluminum hydroxide	2–8	II	2	18–59	28	4 μg *	China	[30]
Zhang et al.	3 and 5 May 2020	2020	CoronaVac	Sinovac	Randomized, double-blind, placebo controlled	Inactivated	Aluminum hydroxide	2–8	II	2	18–59	14	3 μg *	China	[31]
Zhang et al.	3 and 5 May 2020	2020	CoronaVac	Sinovac	Randomized, double-blind, placebo controlled	Inactivated	Aluminum hydroxide	2–8	II	2	18–59	28	3 μg *	China	[31]
Zhang et al.	3 and 5 May 2020	2020	CoronaVac	Sinovac	Randomized, double-blind, placebo controlled	Inactivated	Aluminum hydroxide	2–8	II	2	18–59	14	6 μg *	China	[31]
Zhang et al.	3 and 5 May 2020	2020	CoronaVac	Sinovac	Randomized, double-blind, placebo controlled	Inactivated	Aluminum hydroxide	2–8	II	2	18–59	28	6 μg *	China	[31]
Logunov et al.	18 June and 3 August 2020	2020	Sputnik V	Gamaleya	Non-randomized	Adenovirus-based	No adjuvant	2–8	II	2	18–59	21	10^11^ VP *	Russia	[32]
Logunov et al.	18 June and 3 August 2020	2020	Sputnik V (lyophilised)	Gamaleya	Non-randomized	Adenovirus-based	No adjuvant	2–8	II	2	18–60	21	10^11^ VP *	Russia	[32]
Voysey et al.	23 April and 4 November 2020	2021	AZD1222	Oxford/AstraZeneca	Randomized, single-blind, placebo-controlled	Adenovirus-based	No adjuvant	2–8	II/III	2	18–60	28	2.2 × 10^1^⁰ VP * (1st)/5 × 10^1^⁰ VP * (2nd)	United Kingdom	[38]
Voysey et al.	23 April and 4 November 2020	2021	AZD1222	Oxford/AstraZeneca	Randomized, single-blind, placebo-controlled	Adenovirus-based	No adjuvant	2–8	II/III	2	18≤	28	5 × 10^1^⁰ VP *	United Kingdom	[38]
Pollack et al.	27 July and 14 November 2020	2020	BNT162b2	Pfizer/BioNTech	Randomized, observer-blind, placebo-controlled	mRNA-based	No adjuvant	(−60)–(−80)	II/III	2	18≤	21	30 μg/0.3 ml	Multinational ^1^	[39]
Baden et al.	27 July and 23 October	2020	mRNA-1273	Moderna	Randomized, observer-blind, placebo-controlled	mRNA-based	No adjuvant	−20	III	2	16–89	28	100 μg *	United States	[40]
Voysey et al.	23 April and 4 November 2020	2021	AZD1222	Oxford/AstraZeneca	Randomized, single-blind, placebo-controlled	Adenovirus-based	No adjuvant	2–8	III	2	18–95	28	5 × 10^1^⁰ VP *	Brazil	[38]
Logunov et al.	7 September and 24 November 2020	2021	Sputnik V	Gamaleya	Randomized, double-blind, placebo controlled	Adenovirus-based	No adjuvant	2–8	III	2	18≤	21	10^11^ VP *	Russia	[41]
Sadoff et al.	20 July 2020	2021	Ad26.COV2.S	Johnson & Johnson	Randomized, double-blind, placebo-controlled	Adenoviral vector	No adjuvant	UN	I/II	2	18–55	No	5 × 10^10^	Belgium, US	[42]
Sadoff et al.	20 July 2020	2021	Ad26.COV2.S	Johnson & Johnson	Randomized, double-blind, placebo-controlled	Adenoviral vector	No adjuvant	UN	I/II	2	19–55	No	1 × 10^11^	Belgium, US	[42]
Sadoff et al.	November 2020	2021	Ad26.COV2.S	Johnson & Johnson	Randomized, double-blind, placebo-controlled	Adenoviral vector	No adjuvant	UN	I/II	2	65–83	No	5 × 10^10^	Belgium, US	[42]
Sadoff et al.	November 2020	2021	Ad26.COV2.S	Johnson & Johnson	Randomized, double-blind, placebo-controlled	Adenoviral vector	No adjuvant	UN	I/II	2	65–88	No	1 × 10^11^	Belgium, US	[42]

^1^ From 152 sites worldwide (United States, 130 sites; Argentina, 1; Brazil, 2; South Africa, 4; Germany, 6; and Turkey, 9). UN = unavailable. VP = virus particle; Pro-Subunit = protein subunit; VLP = virus-like particle; Alum = aluminium; Adv= adenovirus; CpG = cytosine-guanine oligodeoxynucleotide; AS03= squalene-based immunologic adjuvant; Algel-IMDG (an imidazoquinoline molecule chemisorbed on alum [Algel]); RCT = randomized control trial. * per 0.5 m. Studies with different reports for the vaccine phase, the vaccine dose, injection concentration, different case, and control group numbers are considered as a separate dataset. More detailed information is provided in Table S3.

**Table 2:** Efficacy of adenovirus-based and mRNA-based COVID-19 vaccines.

Vaccine Type	RCT Phase	Number Studies	Efficacy (%)	95% CI (%)		Included Case N	Heterogeneity Test, *p*-Value
				Lower Limit	Upper Limit		
Adenovirus-based	2/3	4	80.2	0.564	0.927	20771	<0.001
mRNA-based	2/3	2	94.6	0.936	0.954	34041	<0.001

RCT = randomized control trial.

**Table 3:** Efficacy of different COVID-19 vaccines after the first and second doses.

				Vaccine					Placebo				
Shot	Antigen/Antibody	After Injection (Week)	Vaccine Type	Studies N	Efficacy	Lower Limit	Upper Limit	I-Squared	Studies N	Efficacy	Lower Limit	Upper Limit	I-Squared
**After 1st dose**	RBD	2	Adenovirus-based	4	0.603	0.471	0.722	73.8	2	0.004	0.001	0.027	0
			Inactivated	4	0.870	0.734	0.983	93.8	4	0.024	0.008	0.072	0
		3	Adenovirus-based	2	0.976	0.939	0.997	0.0	NA	NA	NA	NA	NA
			VLP	8	0.238	0.091	0.375	84.3	NA	NA	NA	NA	NA
		4	Adenovirus-based	2	0.966	0.942	0.980	0.0	2	0.004	0.001	0.027	0
			Inactivated	4	0.913	0.564	0.958	90.7	4	0.061	0.033	0.110	0
			Pro-Subunit	6	0.628	0.590	0.665	0.0	NA	NA	NA	NA	NA
	S-protein	2	Inactivated	2	0.293	0.182	0.437	0.0	2	0.083	0.032	0.202	0
		3	Pro-Subunit	4	0.790	0.474	0.874	91.0	4	0.021	0.005	0.079	0
		4	Inactivated	2	0.396	0.269	0.539	0.0	2	0.021	0.003	0.134	0
			Pro-Subunit	4	0.873	0.671	0.892	91.9	4	0.021	0.005	0.079	0
	NAb	2	Inactivated	4	0.583	0.210	0.868	95.0	4	0.030	0.013	0.070	0
		3	Pro-Subunit	4	0.551	0.291	0.786	85.0	4	0.021	0.005	0.079	0
		4	Adenovirus-based	2	0.547	0.496	0.596	76.8	2	0.008	0.002	0.031	0
			Inactivated	4	0.691	0.537	0.812	95.0	4	0.025	0.008	0.074	0
			mRNA-based	4	0.702	0.655	0.746	73.9	4	0.010	0.004	0.026	0
**After 2nd dose**	RBD	2	Inactivated	9	0.929	0.876	0.960	61.8	9	0.171	0.077	0.336	85
			mRNA-based	5	0.731	0.532	0.866	86.0	NA	NA	NA	NA	NA
		3	Adenovirus-based	3	0.982	0.980	0.984	0.0	1	0.149	0.139	0.159	0
			VLP	9	0.787	0.581	0.908	78.1	NA	NA	NA	NA	NA
		4	Inactivated	4	0.944	0.842	0.982	15.9	4	0.063	0.026	0.143	0
			Pro-Subunit	6	0.956	0.937	0.970	0.0	NA	NA	NA	NA	NA
	S-protein	2	Inactivated	7	0.941	0.877	0.973	61.4	7	0.290	0.139	0.507	87
			Pro-Subunit	18	0.852	0.719	0.928	62.4	18	0.028	0.014	0.052	0
			mRNA-based	5	0.786	0.725	0.836	0.0	NA	NA	NA	NA	NA
		4	Inactivated	4	0.934	0.842	0.974	15.9	4	0.063	0.026	0.143	0
			Pro-Subunit	14	0.792	0.679	0.873	50.7	15	0.031	0.015	0.061	0
	NAbs	2	Adenovirus-based	1	0.999	0.985	1.000	0.0	NA	NA	NA	NA	NA
			Inactivated	9	0.845	0.724	0.919	86.5	9	0.151	0.068	0.303	85
			Pro-Subunit	23	0.753	0.667	0.823	68.2	19	0.028	0.015	0.052	0
			mRNA-based	9	0.870	0.747	0.938	82.4	4	0.008	0.003	0.024	0
		3	Adenovirus-based	1	0.958	0.955	0.961	0.0	1	0.071	0.065	0.079	0
		4	Adenovirus-based	1	0.999	0.985	1.000	0.0	NA	NA	NA	NA	NA
			Inactivated	4	0.700	0.375	0.901	86.1	4	0.033	0.011	0.099	0
			Pro-Subunit	17	0.759	0.574	0.881	62.9	15	0.031	0.015	0.061	0
			mRNA-based	4	0.995	0.980	0.999	0.0	4	0.016	0.007	0.035	0

S-protein = spike protein, Alum = aluminium, CpG = cytosine-guanine oligodeoxynucleotide, AS03 = squalene-based immunologic adjuvant, RBD = receptor-binding domain, NAb = neutralizing antibody, Pro-Subunit = protein subunit, NA = not available.

**Table 4:** Association of side effects with different COVID-19 vaccines.

Side Effect	Vaccine Type	Phase	Odds Ratio (95% CI)	Included Study	Heterogeneity Test, I-Squared
Site pain	mRNA-based	2/3	83.06 (37.05–186.1)	5	81.33
		1/2	28.26 (16.18–49.35)	17	0
	Adenovirus-based	2/3	13.64 (8.39–22.17)	2	0
		1/2	3.2 (2.7–4)	2	0
	Inactivated	2/3	1.73 (0.667–4.5)	6	46.64
		1/2	3.19 (1.3–7.6)	10	52.14
	Pro-Subunit	2/3	2.14 (1.01–4.5)	4	48.55
		1/2	2.29 (0.48–10.8)	2	29.93
Swelling	Adenovirus-based	1/2/3	1.21 (0.77–1.89)	2	0
	Inactivated	1/2/3	0.402 (0.056–2.90)	2	0
	Pro-Subunit	1/2/3	6.48 (3.09–13.67)	5	0
	mRNA-based	1/2/3	18.79 (4.87–72.40)	3	59.08
Redness	Adenovirus-based	1/2/3	1.35 (0.815–2.25)	4	0
	Pro-Subunit	1/2/3	7.29 (3.70–14.38)	6	0
	mRNA-based	1/2/3	24.40 (18.73–31.77)	1	0
Itch	Adenovirus-based	1/2	3.10 (1.96–4.89)	1	0
	Inactivated	1/2	0.32 (0.02–5.3)	1	0
	Pro-Subunit	1/2	25.44 (7.85–82.40)	6	0
Cough	Adenovirus-based	1/2/3	1.76 (1.20–2.58)	3	0
Fever	Adenovirus-based	1/2/3	1.73 (0.57–5.66)	3	90.5
	Inactivated	1/2/3	0.27 (0.09–0.76)	5	0
	Pro-Subunit	1/2/3	1.17 (0.73–1.86)	4	0
	mRNA-based	1/2/3	36.90 (12.34–105.21)	3	43.31
Headache	mRNA-based	3	4.63 (4.4–4.86)	1	0
		2	2.32 (1.28–4.19)	4	69.20
		1/2	5.13 (2.32–11.31)	5	63.02
	Adenovirus-based	2	2.54 (1.65–3.91)	2	0
		1/2	3.01 (2.35–3.87)	1	0
		3	0.58 (0.49–0.68)	1	0
	Inactivated	2	0.18 (0.02–1.14)	2	0
	Pro-Subunit	2	1.25 (0.33–4.7)	2	0
		1/2	1.99 (1.21–3.26)	14	0
Fatigue	Adenovirus-based	1/2	2.72 (2.2–3.37)	3	0
	Inactivated	1/2	0.39 (0.18–0.82)	7	0
	Pro-Subunit	1/2	2.7 (1.01–7.16)	4	37.2
	mRNA-based	1/2	5.0 (3.42–7.33)	24	48.23
		2–3	4.87 (4.65–5.09)	1	0
		3	6.16 (5.86–6.48)	1	0
Induration	Adenovirus-based	1/2	0.16 (0.05–0.49)	2	46.44
	Inactivated	1/2	0.18 (0.06–0.58)	4	0
	Pro-Subunit	1/2	2.62 (1.23–5.58)	2	0
	mRNA-based	1/2	17.5 (1.96–155.58)	1	0
Vomiting	Adenovirus-based	1/2	2.75 (1.99–3.82)	3	0
	Inactivated	1/2	0.32 (0.02–5.38)	1	0
	mRNA-based	1/2	8.71 (4.38–17.34)	8	0
		2–3	4.87 (4.65–5.09)	1	0
		3	6.16 (5.86–6.48)	1	0
Diarrhea	Adenovirus-based	1/2	2.51 (1.12–5.63)	2	0
	Inactivated	1/2	0.60 (0.13–2.83)	3	0
	mRNA-based	1/2	0.54 (0.27–1.10)	5	0
Myalgia	Adenovirus-based	1/2	4.59 (3.58–5.89)	3	0
	Inactivated	1/2	1.43 (0.25–8.08)	2	0
	Pro-Subunit		2.92 (0.57–8.75)	8	53.30
	mRNA-based	1/2	10.71 (6.51–17.60)	10	33.74
		2/3	7.0 (6.57–7.47)	1	0
		3	1.43 (0.25–8.08)	1	0
Arthralgia	Adenovirus-based	2/3	4.06 (2.99–5.57)	3	0
	Pro-Subunit	2/3	1.34 (0.47–3.83)	4	4.833
	mRNA-based	2/3	9.67 (1.27–76.90)	3	67.97
Chills	Adenovirus-based	2/3	10.61 (7.60–14.83)	1	0
	mRNA-based	2/3	13.11 (7.19–23.89)	8	3.82
Pruritus	Adenovirus-based	2/3	0.54 (0.23–1.25)	2	0
	mRNA-based	2/3	17.50 (1.98–155.58)	1	0

**Table 5:** Side effects of COVID-19 vaccines based on different adjuvant types.

Side Effect		Adjuvant Type	Phase	Odds Ratio (95% CI)	Included Study	Heterogeneity Test, I-Squared
Systemic	Fatigue	Alum	2/3	0.392 (0.18–0.82)	7	0
		Matrix-M1	2/3	3.70 (1.36–10.02)	3	24.81
		No adjuvant	2/3	4.43 (2.62–7.49)	6	54.08
	Vomiting	Alum	2/3	0.325 (0.02–5.30)	1	0
		No adjuvant	2/3	3.46 (1.45–8.26)	7	0
	Fever	Alum	2/3	0.85 (0.51–1.43)	9	20.78
		No adjuvant	2/3	2.96 (1.22–7.17)	2	68.19
	Myalgia	Alum	2/3	1.43 (0.25–8.0)	2	0
		AS03	2/3	14.331 (3.39–60.56)	3	0
		CpG/Alum	2/3	2.42 (0.13–44.50)	1	0
		Matrix-M1	2/3	1.57 (0.26–9.4)	3	67.96
		No adjuvant	2/3	9.66 (3.97–23.47)	8	49.99
	Diarrhea	Alum	2/3	0.608 (0.13–2.87)	3	0
		No adjuvant	2/3	0.89 (0.40–1.97)	6	50.47
Local	Injection site pain	Alum	2/3	2.40 (1.51–3.83)	22	44.55
		No adjuvant	2/3	25.61 (13.31–49.30)	7	36.60
	Itch	Alum	2/3	13.20 (3.23–53.90)	7	40.58
	Swelling	Alum	2/3	3.83 (1.52–9.64)	7	37.52
	Redness	Alum	2/3	7.29 (3.7–14.39)	6	0
		No adjuvant	2/3	0.923 (0.23–3.6)	2	0

Alum = aluminum, CpG = cytosine-guanine oligodeoxynucleotide, AS03 = squalene-based immunologic adjuvant.

## Data Availability

All data needed to evaluate the conclusions in the paper are included and/or available within the Supplementary Materials. Additional data related to this paper may be requested from the authors.

## Supplementary Materials

The following are available online at https://www.mdpi.com/article/10.3390/vaccines9050467/s1, Figure S1. Meta-analysis A. Forest plot, B. Funnel plot for the injection site pain as a side effect of different COVID 19 vaccine in phase 2/3 RCT, Figure S2. Meta-analysis A. Forest plot, B. Funnel plot for the injection site pain as a side effect of different COVID 19 vaccine in phase 1/2 RCT, Figure S3. Meta-analysis A. Forest plot, B. Funnel plot for the Swelling as a side effect of different COVID 19 vaccine in phase 1/2/3 RCT, Figure S4. Meta-analysis A. Forest plot, B. Funnel plot for the Redness as a side effect of different COVID 19 vaccine in phase 1/2/3 RCT, Figure S5. Meta-analysis A. Forest plot, B. Funnel plot for the Itch as a side effect of different COVID 19 vaccine in phase 1/2 RCT, Figure S6. Meta-analysis A. Forest plot, B. Funnel plot for the Cough as a side effect of different COVID 19 vaccine in phase 1/2/3 RCT, Figure S7. Meta-analysis A. Forest plot, B. Funnel plot for the Fever as a side effect of different COVID 19 vaccine in phase 1/2/3 RCT, Figure S8. Meta-analysis A. Forest plot, B. Funnel plot for the Headache as a side effect of different COVID 19 vaccine in phase 1/2 RCT, Figure S9. Meta-analysis A. Forest plot, B. Funnel plot for the Headache as a side effect of different COVID 19 vaccine in phase 2 RCT, Figure S10. Meta-analysis A. Forest plot, B. Funnel plot for the Headache as a side effect of different COVID 19 vaccine in phase 3 RCT, Figure S11. Meta-analysis A. Forest plot, B. Funnel plot for the Fatigue as a side effect of different COVID 19 vaccine in phase 1/2 RCT, Figure S12. Meta-analysis A. Forest plot, B. Funnel plot for the Fatigue as a side effect of different COVID 19 vaccine in both mRNA-based vaccine, in RCT 2/3, and 3, Figure S13. Meta-analysis A. Forest plot, B. Funnel plot for the Induration as a side effect of different COVID 19 vaccine in RCT ½, Figure S14. Meta-analysis A. Forest plot, B. Funnel plot for the Vomiting as a side effect of different COVID 19 vaccine in RCT ½, Figure S15. Meta-analysis A. Forest plot, B. Funnel plot for the Vomiting as a side effect of different COVID 19 vaccine in both mRNA-based vaccine, in RCT 2/3, and 3, Figure S16. Meta-analysis A. Forest plot, B. Funnel plot for the Diarrhea as a side effect of different COVID 19 vaccine in RCT ½, Figure S17. Meta-analysis A. Forest plot, B. Funnel plot for the Myalgia as a side effect of different COVID 19 vaccine in RCT ½, Figure S18. Meta-analysis A. Forest plot, B. Funnel plot for the Myalgia as a side effect of different COVID 19 vaccine in RCT 3, Figure S19. Meta-analysis A. Forest plot, B. Funnel plot for the Arthralgia as a side effect of different COVID 19 vaccine in RCT 2/3, Figure S20. Meta-analysis A. Forest plot, B. Funnel plot for the Chills as a side effect of different COVID 19 vaccine in RCT 2/3, Figure S21. Meta-analysis A. Forest plot, B. Funnel plot for the Pruritus as a side effect of different COVID 19 vaccine in RCT 2/3, Figure S22. Meta-analysis A. Forest plot, B. Funnel plot for the Fatigue as a side effect based on the adjuvant type in phase 2/3 RCT, Figure S23. Meta-analysis A. Forest plot, B. Funnel plot for the Diarrhea as a side effect based on the adjuvant type in phase 2/3 RCT, Figure S24. Meta-analysis A. Forest plot, B. Funnel plot for the Injection site pain as a side effect based on the adjuvant type in phase 2/3 RCT, Figure S25. Meta-analysis A. Forest plot, B. Funnel plot for the Itch as a side effect based on the adjuvant type in phase 2/3 RCT, Figure S26. Meta-analysis A. Forest plot, B. Funnel plot for the Myalgia as a side effect based on the adjuvant type in phase 2/3 RCT, Figure S27. Meta-analysis A. Forest plot, B. Funnel plot for the Swelling as a side effect based on the adjuvant type in phase 2/3 RCT, Figure S28. Meta-analysis A. Forest plot, B. Funnel plot for the Redness as a side effect based on the adjuvant type in phase 2/3 RCT, Figure S29. Meta-analysis A. Forest plot, B. Funnel plot for the Vomiting as a side effect based on the adjuvant type in phase 2/3 RCT, Figure S30. Meta-analysis A. Forest plot, B. Funnel plot for the Fever as a side effect based on the adjuvant type in phase 2/3 RCT, Table S1. Search strategy, Table S2. Quality assessment of included studies.

## Abbreviations

PRISMA	The Preferred Reporting Items for Systematic Reviews and Meta-Analyses Statement
MeSH	Medical subject headings
WHO	World Health Organization
CDC	Center for Disease Control
RCT	Randomized clinical trial
COVID-19	Coronavirus disease 2019
alum	Potassium aluminum sulfate
VLPs	Virus-like particles
ORs	Odds ratios
6-HB	six-helical bundle
SARS-CoV-2	Severe acute respiratory syndrome coronavirus 2
95% CI	95% confidence interval
GMT	Geometric mean titer
RBD	Receptor-binding domain
PLpro	Papain-like proteases
3CLpro	Cysteine-like protease
NAb	Neutralizing antibody
Pro-subunit	Protein subunit
VLP	Virus-like particle
BMI	Body mass index
CFR	Case fatality ratio
RNA	Ribonucleic acid
messenger RNA	mRNA
S-protein	Spike protein
ACE2	Angiotensin-converting enzyme 2
nsp	Non-structural proteins
IM	Intramuscular
