# Supplementary material for: Efficacy and Safety of COVID-19 Vaccines: A Systematic Review and Meta-Analysis of Randomized Clinical Trials
Source: Vaccines (Basel). 2021 May 6;9(5):467. doi: 10.3390/vaccines9050467 (PMC8148145; doi:10.3390/vaccines9050467)
Supplement: Supplementary file 1 [file vaccines-09-00467-s001.zip › vaccines-1192470-supplementary.pdf]

Supplementary A

Efficacy and safety of COVID-19 Vaccines: a systematic review and meta-analysis of randomized clinical trials

Ali Pormohammad[1], Mohammad Zarei[2,3], Saied Ghorbani[4], Mehdi Mohammadi[5], Mohammad Hossein Razizadeh[6], Raymond J. Turner[7]*

[1]Department of Biological Sciences, University of Calgary, Calgary, AB, Canada ali.pormohammad@ucalgary.ca

[2]John B. Little Center for Radiation Sciences, Harvard T.H. Chan School of Public Health, Boston, MA 02115. mzarei@hsph.harvard.edu

[3] Harvard Medical School, Boston, Massachusetts, USA. mzarei@hsph.harvard.edu

[4]Department of Virology, Faculty of Medicine, Iran University of Medical Science, Tehran, Iran. vet.s.ghorbani@gmail.com

[5]Department of Biological Sciences, University of Calgary, Calgary, AB, Canada mehdi.mohammadiashan@ucalgary.ca

[6]Department of Virology, Faculty of Medicine, Iran University of Medical Science, Tehran, Iran. razizadeh.mh@iums.ac.ir

[7]Department of Biological Sciences, University of Calgary, Calgary, AB, Canada turnerr@ucalgary.ca

***Corresponding Authors:**

Raymond J. Turner, Professor, Department of Biological Sciences, University of Calgary

Phone No: +1 (403) 220-4308 Mail: turnerr@ucalgary.ca

Table S1. Search strategy.

ID	ID Search Terms	Results
**Medlin (PubMed)**		
**#1**	covid-19 AND vaccine	**7952**
**#2**	Sars-cov-2 AND vaccine	**5736**
**#3**	covid-19 AND vaccine AND clinical trial	**536**
**#4**	Covid-19 vaccine trials	**957**
**Web of Science**		
**#1**	covid-19 AND vaccine	**3970**
**#2**	Sars-cov-2 AND vaccine	**2790**
**#3**	covid-19 AND vaccine AND clinical trial	**582**
**#4**	Covid-19 vaccine trials	**783**
**Scopus**		
**#1**	covid-19 AND vaccine	**14**
**#2**	Sars-cov-2 AND vaccine	**14**
**#3**	covid-19 AND vaccine AND clinical trial	**3955**
**#4**	Covid-19 vaccine trials	**29**
**EMBASE**		
**#1**	covid-19 AND vaccine	**3213**
**#2**	Sars-cov-2 AND vaccine	**2105**
**#3**	covid-19 AND vaccine AND clinical trial	**87**
**#4**	Covid-19 vaccine trials	**67**

Table S2. Quality assessment of included studies

Study	Randomization		Blinding		An account of all patients	Quality Score (out of 5)
	1 point if randomization is mentioned	1 point if the method of randomization is appropriate	1 point if blinding is mentioned	1 point if the method of blinding is appropriate	1 point if the fate of all patients in the trial is known	
Anderson et al.	1	1	1	1	0	4
Baden et al.	1	1	1	1	0	4
Chu et al.	1	1	1	1	0	4
Ella et al.	1	1	1	1	0	4
Folegatti et al.	1	1	1	1	0	4
Jackson et al.	1	1	1	1	0	4
Keech et al.	1	1	1	1	0	4
Logunov et al.	1	1	1	1	0	4
Mulligan et al.	1	1	1	1	0	4
Kremsner et al.	1	1	1	1	0	4
Polack et al.	1	1	1	1	0	4
Ramasamy et al.	1	1	1	1	0	4
Richmond et al.	1	1	1	1	1	5
Sahin et al.	1	1	1	1	0	4
Voysey et al.	1	1	1	1	0	4
Walsh et al.	1	1	1	1	0	4
Ward et al.	1	1	1	1	0	4
Xia et al.	1	1	1	1	0	4
Xia et al.	1	1	1	1	0	4
Yang et al.	1	1	1	1	0	4
Zhang et al.	1	1	1	1	1	5
Zhu et al.	1	1	1	1	0	4
Sadoff et al	1	1	1	1	0	4

Supplementary Figures:

A

Meta Analysis

Group by Vaccine type	Study name	Odds ratio	Lower limit	Upper limit	Z-Value	p-Value	site pain / Total: Vaccine	site pain / Total: Placebo
Adeno vector	Zhu et al.	14.036	7.205	27.344	7.764	0.000	145 / 253	11 / 126
Adeno vector	Zhu1 et al.	13.206	6.496	26.847	7.129	0.000	72 / 129	11 / 126
Adeno vector		13.640	8.391	22.174	10.540	0.000		
Inactivated	Xia et al.	0.203	0.032	1.285	-1.693	0.090	2 / 84	3 / 28
Inactivated	Xia1 et al.	4.500	0.558	36.287	1.412	0.158	12 / 84	1 / 28
Inactivated	Zhang et al.	4.462	0.991	20.089	1.948	0.051	16 / 120	2 / 60
Inactivated	Zhang1 et al.	0.744	0.121	4.574	-0.320	0.749	3 / 120	2 / 60
Inactivated	Zhang2 et al.	3.830	0.841	17.439	1.736	0.082	14 / 120	2 / 60
Inactivated	Zhang3 et al.	1.796	0.362	8.925	0.716	0.474	7 / 120	2 / 60
Inactivated		1.734	0.667	4.506	1.130	0.259		
Pro-Subunit	Yang et al.	0.828	0.247	2.773	-0.307	0.759	5 / 150	6 / 150
Pro-Subunit	Yang1 et al.	1.352	0.458	3.996	0.546	0.585	8 / 150	6 / 150
Pro-Subunit	Yang2 et al.	3.955	1.428	10.950	2.646	0.008	18 / 150	5 / 150
Pro-Subunit	Yang3 et al.	3.955	1.428	10.950	2.646	0.008	18 / 150	5 / 150
Pro-Subunit		2.142	1.010	4.544	1.986	0.047		
RNA	Chu et al.	40.444	17.424	93.881	8.611	0.000	80 / 100	9 / 100
RNA	Chu1 et al.	96.000	31.519	292.397	8.032	0.000	80 / 100	4 / 100
RNA	Chu2 et al.	192.111	62.034	594.939	9.117	0.000	95 / 100	9 / 100
RNA	Chu3 et al.	276.000	80.365	947.875	8.928	0.000	92 / 100	4 / 100
RNA	Baden et al.	36.414	34.123	38.859	108.434	0.000	13387 / 15181	2580 / 15170
RNA		83.060	37.058	186.165	10.733	0.000		
Overall		9.906	7.049	13.922	13.208	0.000		

Odds ratio and 95% CI

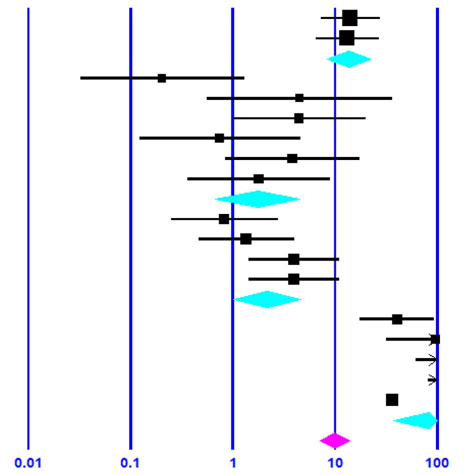

Groups		Effect size and 95% interval			Test of null (2-Tail)		Heterogeneity			
Group	Number Studies	Point estimate	Lower limit	Upper limit	Z-value	P-value	Q-value	df (Q)	P-value	I-squared
**Mixed effects analysis**										
Adeno vector	2	13.640	8.391	22.174	10.540	0.000	0.015	1	0.902	0.000
Inactivated	6	1.734	0.667	4.506	1.130	0.259	9.372	5	0.095	46.648
Pro-Subunit	4	2.142	1.010	4.544	1.986	0.047	5.832	3	0.120	48.559
RNA	5	83.060	37.058	186.165	10.733	0.000	21.435	4	0.000	81.339

B

Funnel Plot of Standard Error by Log odds ratio

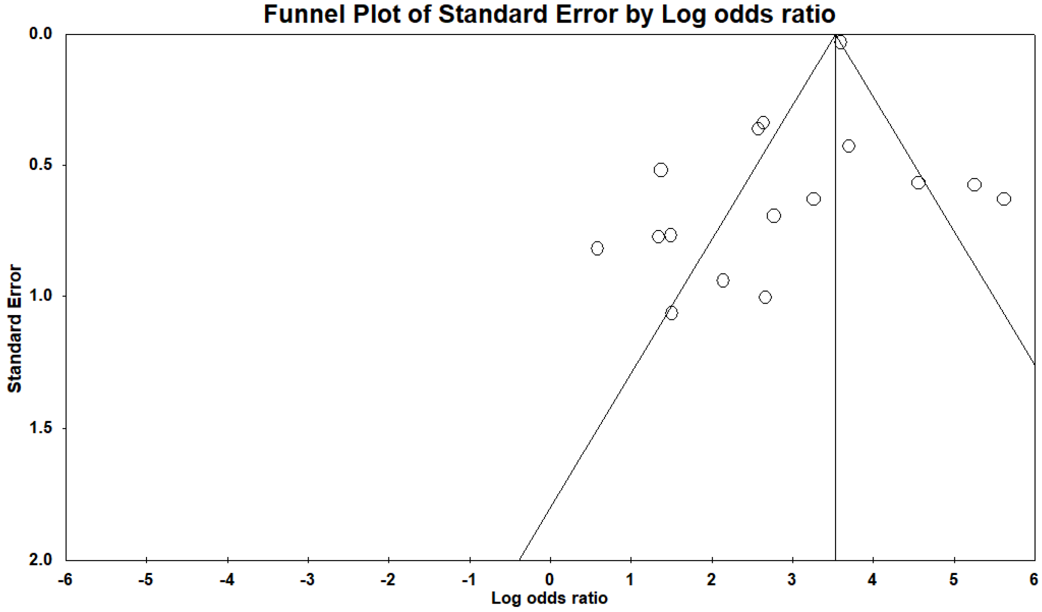

Fig S1. Meta-analysis A. Forest plot, B. Funnel plot for the injection site pain as a side effect of different COVID 19 vaccine in phase 2/3 RCT.

Meta Analysis

Group by Vaccine type	Study name	Odds ratio	Lower limit	Upper limit	Z-Value	p-Value	Swelling / Total: Vaccine	Swelling / Total: Placebo
Adeno vector	Folegatti1 et al.	1.210	0.642	2.284	0.590	0.555	22 / 543	18 / 534
Adeno vector	Folegatti et al.	1.210	0.642	2.284	0.590	0.555	22 / 543	18 / 534
Adeno vector		1.210	0.773	1.896	0.834	0.404		
Inactivated	Xia.1 et al.	0.325	0.020	5.380	-0.785	0.433	1 / 84	1 / 28
Inactivated	Zhang.3 et al.	0.496	0.030	8.067	-0.493	0.622	1 / 120	1 / 60
Inactivated		0.402	0.056	2.907	-0.903	0.367		
Pro-Subunit	Yang et al.	3.083	0.612	15.528	1.365	0.172	6 / 150	2 / 150
Pro-Subunit	Yang1 et al.	4.723	1.003	22.242	1.964	0.050	9 / 150	2 / 150
Pro-Subunit	Yang2 et al.	12.047	2.771	52.366	3.319	0.001	21 / 150	2 / 150
Pro-Subunit	Yang3 et al.	11.385	2.611	49.639	3.237	0.001	20 / 150	2 / 150
Pro-Subunit	Yang.1 et al.	1.615	0.060	43.247	0.286	0.775	1 / 20	0 / 10
Pro-Subunit		6.489	3.090	13.630	4.939	0.000		
RNA	Chu et al.	5.211	0.598	45.426	1.494	0.135	5 / 100	1 / 100
RNA	Chu2 et al.	11.000	1.381	87.641	2.265	0.024	10 / 100	1 / 100
RNA	Baden et al.	41.140	31.118	54.389	26.095	0.000	1850 / 15181	51 / 15170
RNA		18.792	4.877	72.409	4.262	0.000		
Overall		2.127	1.479	3.058	4.073	0.000		

Odds ratio and 95% CI

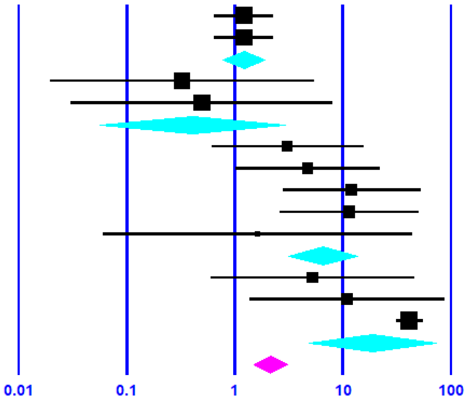

Groups		Effect size and 95% interval			Test of null (2-Tail)		Heterogeneity			
Group	Number Studies	Point estimate	Lower limit	Upper limit	Z-value	P-value	Q-value	df (Q)	P-value	I-squared
**Mixed effects analysis**										
Adeno vector	2	1.210	0.773	1.896	0.834	0.404	0.000	1	1.000	0.000
Inactivated	2	0.402	0.056	2.907	-0.903	0.367	0.044	1	0.835	0.000
Pro-Subunit	5	6.489	3.090	13.630	4.939	0.000	2.903	4	0.574	0.000
RNA	3	18.792	4.877	72.409	4.262	0.000	4.888	2	0.087	59.086

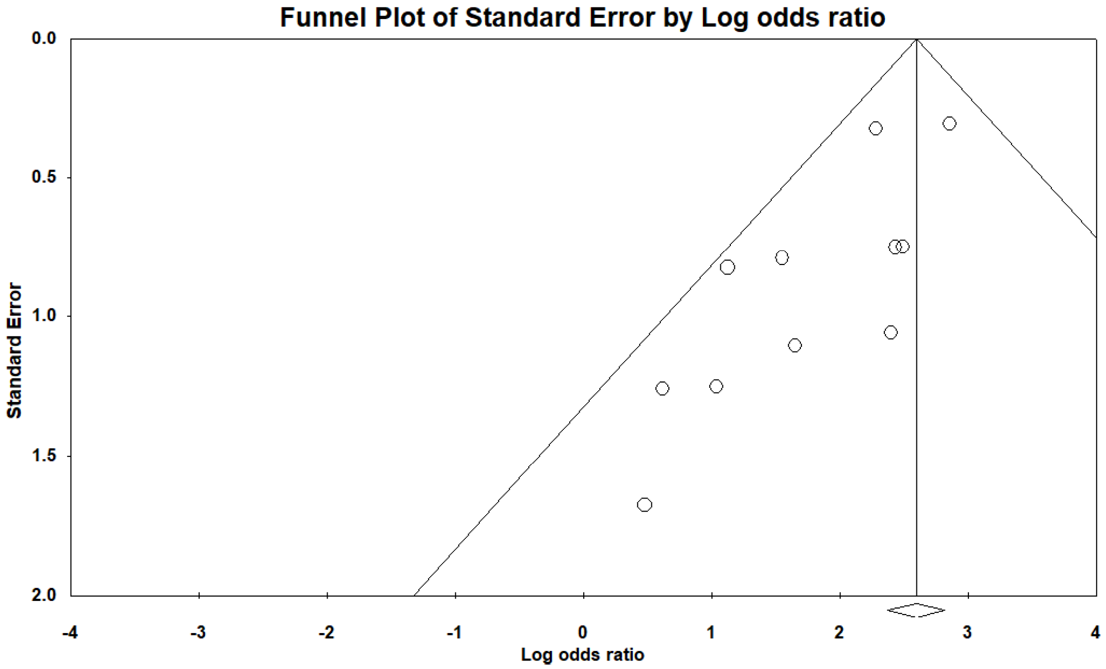

Fig S2. Meta-analysis A. Forest plot, B. Funnel plot for the Swelling as a side effect of different COVID 19 vaccine in phase 1/2/3 RCT.

A

Meta Analysis

Group by Vaccine type	Study name	Odds ratio	Lower limit	Upper limit	Z-Value	p-Value	Redness / Total: Vaccine	Redness / Total: Placebo
Adeno vector	Zhu et al.	1.250	0.239	6.534	0.264	0.791	5 / 253	2 / 126
Adeno vector	Zhu1 et al.	0.484	0.043	5.410	-0.589	0.556	1 / 129	2 / 126
Adeno vector	Folegatti1 et al.	1.444	0.664	3.140	0.926	0.355	16 / 543	11 / 534
Adeno vector	Folegatti et al.	1.444	0.664	3.140	0.926	0.355	16 / 543	11 / 534
Adeno vector		1.356	0.815	2.258	1.172	0.241	38 / 1468	26 / 1320
Pro-Subunit	Yang et al.	6.435	1.415	29.269	2.409	0.016	12 / 150	2 / 150
Pro-Subunit	Yang1 et al.	6.435	1.415	29.269	2.409	0.016	12 / 150	2 / 150
Pro-Subunit	Yang2 et al.	14.095	3.267	60.811	3.547	0.000	24 / 150	2 / 150
Pro-Subunit	Yang3 et al.	12.047	2.771	52.366	3.319	0.001	21 / 150	2 / 150
Pro-Subunit	Yang. et al.	2.250	0.217	23.324	0.680	0.497	4 / 20	1 / 10
Pro-Subunit	Yang.1 et al.	2.250	0.217	23.324	0.680	0.497	4 / 20	1 / 10
Pro-Subunit		7.298	3.701	14.393	5.736	0.000	77 / 640	10 / 620
RNA	Baden et al.	24.402	18.739	31.775	23.714	0.000	1300 / 15181	58 / 15170
RNA		24.402	18.739	31.775	23.714	0.000	1300 / 15181	58 / 15170
Overall		12.426	9.956	15.509	22.285	0.000	1415 / 17289	94 / 17110

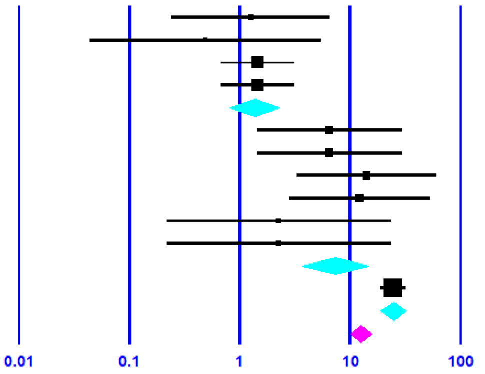

Groups		Effect size and 95% interval			Test of null (2-Tail)		Heterogeneity			
Group	Number Studies	Point estimate	Lower limit	Upper limit	Z-value	P-value	Q-value	df (Q)	P-value	I-squared
**Mixed effects analysis**										
Adeno vector	4	1.356	0.815	2.258	1.172	0.241	0.758	3	0.859	0.000
Pro-Subunit	6	7.298	3.701	14.393	5.736	0.000	3.224	5	0.666	0.000
RNA	1	24.402	18.739	31.775	23.714	0.000	0.000	0	1.000	0.000

B

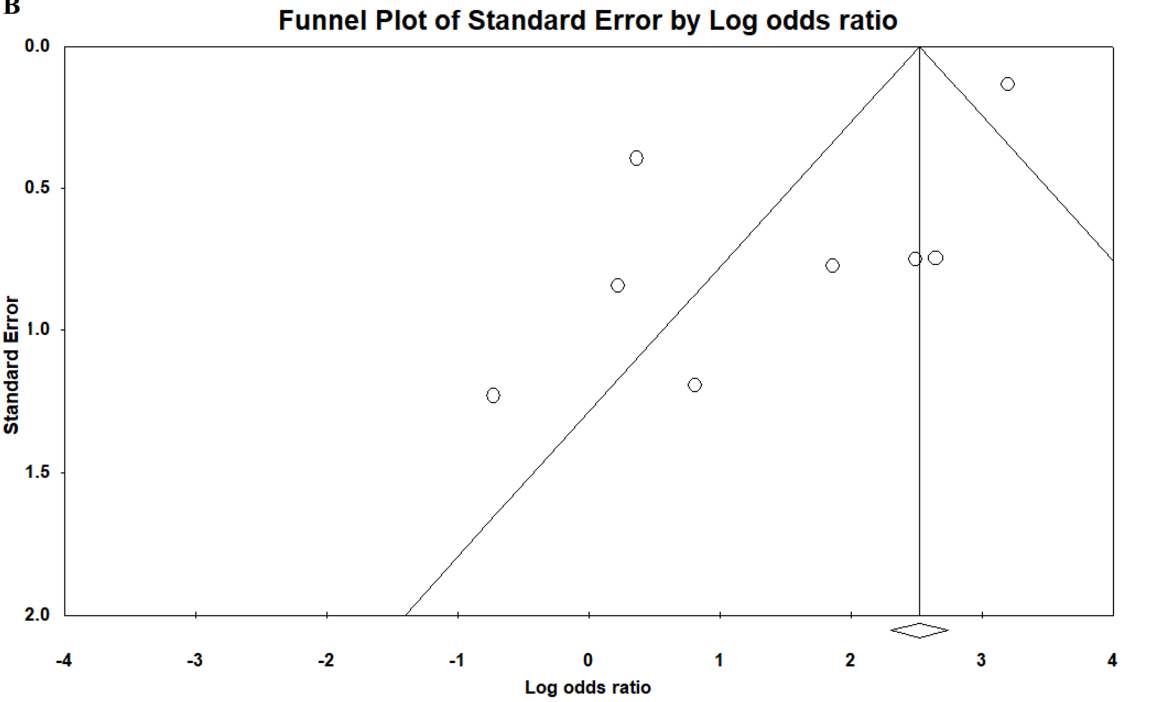

Fig S3. Meta-analysis A. Forest plot, B. Funnel plot for the Redness as a side effect of different COVID 19 vaccine in phase 1/2/3 RCT.

A

Meta Analysis

Group by Vaccine type	Study name	Statistics for each study					Itch / Total	
		Odds ratio	Lower limit	Upper limit	Z-Value	p-Value	Vaccine	Placebo
Adeno vector	Folegatti1 et al.	3.103	1.966	4.896	4.866	0.000	77 / 543	27 / 534
Adeno vector		3.103	1.966	4.896	4.866	0.000	77 / 543	27 / 534
Inactivated	Xia.1 et al.	0.325	0.020	5.380	-0.785	0.433	1 / 84	1 / 28
Inactivated		0.325	0.020	5.380	-0.785	0.433	1 / 84	1 / 28
Pro-Subunit	Yang et al.	20.208	1.165	350.431	2.065	0.039	9 / 150	0 / 150
Pro-Subunit	Yang1 et al.	31.974	1.889	541.113	2.401	0.016	14 / 150	0 / 150
Pro-Subunit	Yang2 et al.	73.082	4.420	1208.322	2.998	0.003	29 / 150	0 / 150
Pro-Subunit	Yang3 et al.	64.068	3.865	1061.928	2.904	0.004	26 / 150	0 / 150
Pro-Subunit	Yang. et al.	5.727	0.279	117.648	1.132	0.258	4 / 20	0 / 10
Pro-Subunit	Yang.1 et al.	11.667	0.596	228.349	1.619	0.105	7 / 20	0 / 10
Pro-Subunit		25.446	7.858	82.401	5.398	0.000	89 / 640	0 / 620
Overall		3.861	2.536	5.879	6.299	0.000	167 / 1267	28 / 1182

Odds ratio and 95% CI

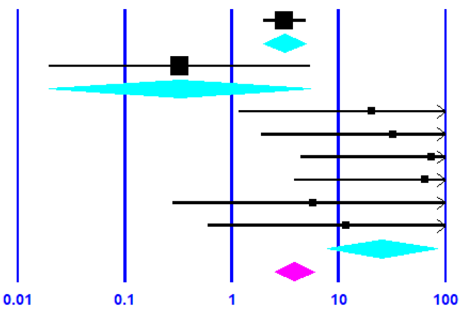

Groups		Effect size and 95% interval			Test of null (2-Tail)		Heterogeneity			
Group	Number Studies	Point estimate	Lower limit	Upper limit	Z-value	P-value	Q-value	df (Q)	P-value	I-squared
**Mixed effects analysis**										
Adeno vector	1	3.103	1.966	4.896	4.866	0.000	0.000	0	1.000	0.000
Inactivated	1	0.325	0.020	5.380	-0.785	0.433	0.000	0	1.000	0.000
Pro-Subunit	6	25.446	7.858	82.401	5.398	0.000	2.208	5	0.820	0.000

B

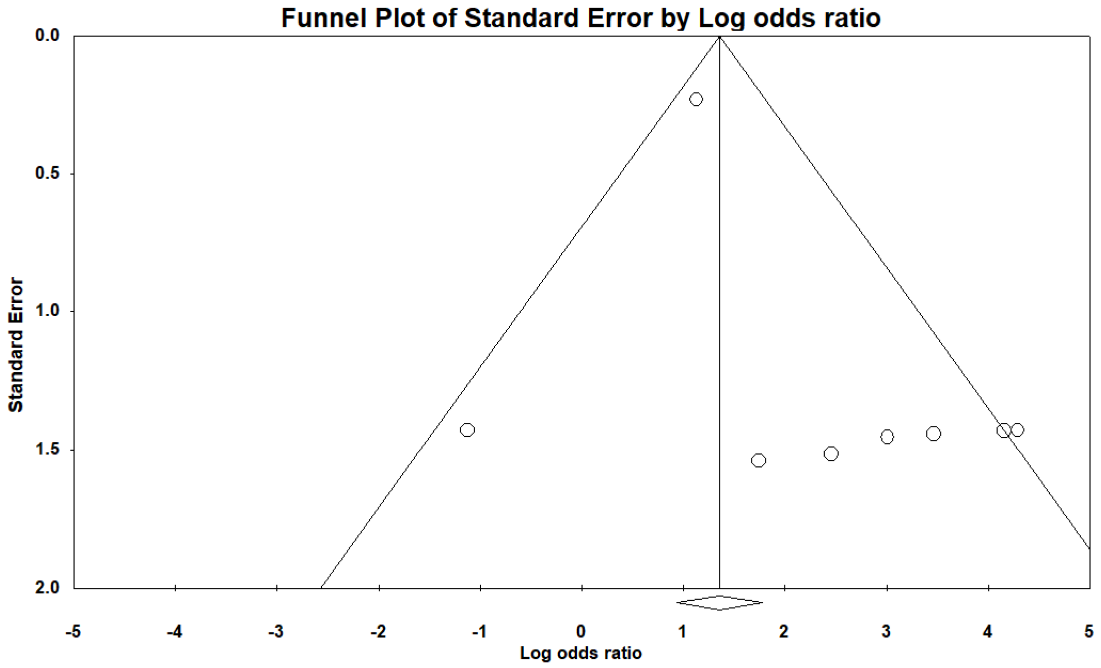

Fig S4. Meta-analysis A. Forest plot, B. Funnel plot for the Itch as a side effect of different COVID 19 vaccine in phase 1/2 RCT.

A

Meta Analysis

Group by Vaccine type	Study name	Odds ratio	Lower limit	Upper limit	Z-Value	p-Value	Vaccine	Placebo
		Statistics for each study					site pain / Total	
Adeno vector	Folegatti1 et al.	3.231	2.518	4.145	9.218	0.000	356 / 543	198 / 534
Adeno vector	Folegatti et al.	3.231	2.518	4.145	9.218	0.000	356 / 543	198 / 534
Adeno vector		3.231	2.708	3.853	13.036	0.000		
Inactivated	Xia et al.	2.895	0.503	16.674	1.190	0.234	5 / 24	2 / 24
Inactivated	Xia1 et al.	0.478	0.040	5.658	-0.585	0.558	1 / 24	2 / 24
Inactivated	Xia2 et al.	3.667	0.658	20.421	1.483	0.138	6 / 24	2 / 24
Inactivated	Xia3 et al.	2.739	0.294	25.537	0.885	0.376	9 / 32	1 / 8
Inactivated	Xia4 et al.	0.226	0.013	4.065	-1.009	0.313	1 / 32	1 / 8
Inactivated	Xia5 et al.	1.960	0.205	18.721	0.584	0.559	7 / 32	1 / 8
Inactivated	Zhang et al.	26.714	4.909	145.377	3.801	0.000	17 / 24	2 / 24
Inactivated	Zhang1 et al.	1.333	0.264	6.739	0.348	0.728	4 / 24	3 / 23
Inactivated	Zhang2 et al.	26.714	4.909	145.377	3.801	0.000	17 / 24	2 / 24
Inactivated	Zhang3 et al.	2.745	0.613	12.292	1.320	0.187	7 / 24	3 / 23
Inactivated		3.190	1.334	7.632	2.607	0.009		
Pro-Subunit	Yang et al.	1.000	0.150	6.671	0.000	1.000	4 / 20	2 / 10
Pro-Subunit	Yang1 et al.	4.889	0.822	29.060	1.745	0.081	11 / 20	2 / 10
Pro-Subunit		2.289	0.484	10.826	1.045	0.296		
RNA	Kremsner et al.	32.348	4.074	256.819	3.289	0.001	24 / 47	1 / 32
RNA	Kremsner1 et al.	75.286	9.346	606.446	4.060	0.000	34 / 48	1 / 32
RNA	Kremsner2 et al.	51.667	6.485	411.622	3.726	0.000	30 / 48	1 / 32
RNA	Kremsner3 et al.	95.818	11.685	785.747	4.250	0.000	34 / 45	1 / 32
RNA	Kremsner4 et al.	113.667	12.767	1011.960	4.243	0.000	22 / 28	1 / 32
RNA	Walsh5 et al.	25.000	2.921	213.986	2.938	0.003	10 / 12	2 / 12
RNA	Walsh7 et al.	55.000	4.300	703.432	3.082	0.002	11 / 12	2 / 12
RNA	Walsh9 et al.	105.000	4.521	2438.704	2.900	0.004	12 / 12	2 / 12
RNA	Walsh11 et al.	4.900	0.700	34.300	1.601	0.109	7 / 12	2 / 9
RNA	Walsh12 et al.	4.000	0.363	44.113	1.132	0.258	4 / 12	1 / 9
RNA	Walsh13 et al.	17.500	1.968	155.592	2.567	0.010	10 / 12	2 / 9
RNA	Walsh14 et al.	11.200	1.042	120.363	1.994	0.046	7 / 12	1 / 9
RNA	Walsh15 et al.	17.500	1.968	155.592	2.567	0.010	10 / 12	2 / 9
RNA	Walsh16 et al.	16.000	1.451	176.451	2.264	0.024	8 / 12	1 / 9
RNA	Mulligan et al.	17.500	1.968	155.592	2.567	0.010	10 / 12	2 / 9
RNA	Mulligan1 et al.	75.000	3.155	1782.778	2.671	0.008	12 / 12	2 / 9
RNA	Mulligan2 et al.	75.000	3.155	1782.778	2.671	0.008	12 / 12	2 / 9
RNA		28.265	16.187	49.357	11.749	0.000		
Overall		3.882	3.295	4.575	16.197	0.000		

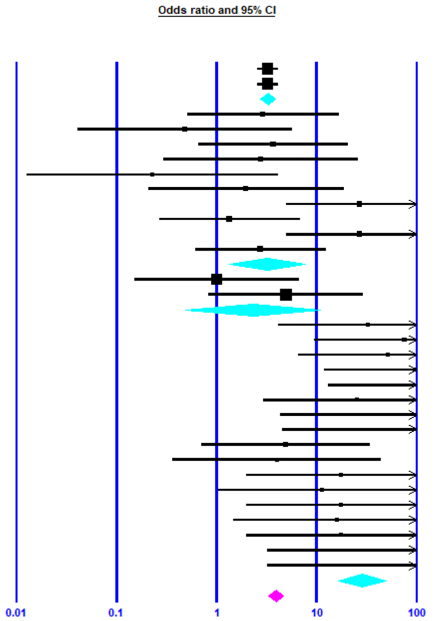

Group	Number Studies	Point estimate	Lower limit	Upper limit	Z-value	P-value	Q-value	df (Q)	P-value	I-squared
Groups		Effect size and 95% interval			Test of null (2-Tail)		Heterogeneity			
**Mixed effects analysis**										
Adeno vector	2	3.231	2.708	3.853	13.036	0.000	0.000	1	1.000	0.000
Inactivated	10	3.190	1.334	7.632	2.607	0.009	18.808	9	0.027	52.149
Pro-Subunit	2	2.289	0.484	10.826	1.045	0.296	1.427	1	0.232	29.936
RNA	17	28.265	16.187	49.357	11.749	0.000	12.728	16	0.693	0.000
Total between							32.964	27	0.198	
Overall	31	3.882	3.295	4.575	16.197	0.000	53.556	3	0.000	
							86.520	30	0.000	65.326

B

Funnel Plot of Standard Error by Log odds ratio

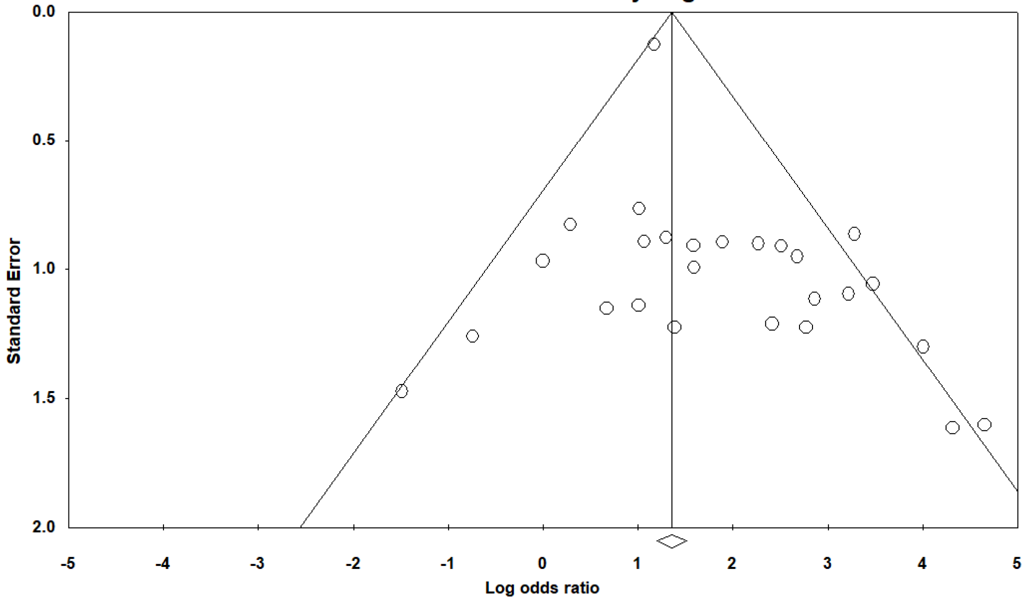

Fig S5. Meta-analysis A. Forest plot, B. Funnel plot for the injection site pain as a side effect of different COVID 19 vaccine in phase 1/2 RCT.

Meta Analysis

Group by Vaccine type	Study name	Odds ratio	Lower limit	Upper limit	Z-Value	p-Value	Vaccine (Cough / Total)	Placebo (Cough / Total)
Adeno vector	Zhu et al.	2.041	0.566	7.369	1.090	0.276	12 / 253	3 / 126
Adeno vector	Zhu1 et al.	0.646	0.106	3.931	-0.475	0.635	2 / 129	3 / 126
Adeno vector	Logunov2 et al.	1.828	1.212	2.758	2.877	0.004	150 / 14964	27 / 4902
Adeno vector		1.762	1.202	2.583	2.902	0.004	164 / 15346	33 / 5154
Overall		1.762	1.202	2.583	2.902	0.004	164 / 15346	33 / 5154

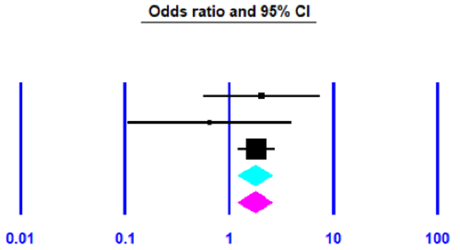

Group	Number Studies	Point estimate (Effect size and 95% interval)	Lower limit	Upper limit	Z-value (Test of null (2-Tail))	P-value	Q-value (Heterogeneity)	df (Q)	P-value	I-squared
Mixed effects analysis										
Adeno vector	3	1.762	1.202	2.583	2.902	0.004	1.268	2	0.530	0.000

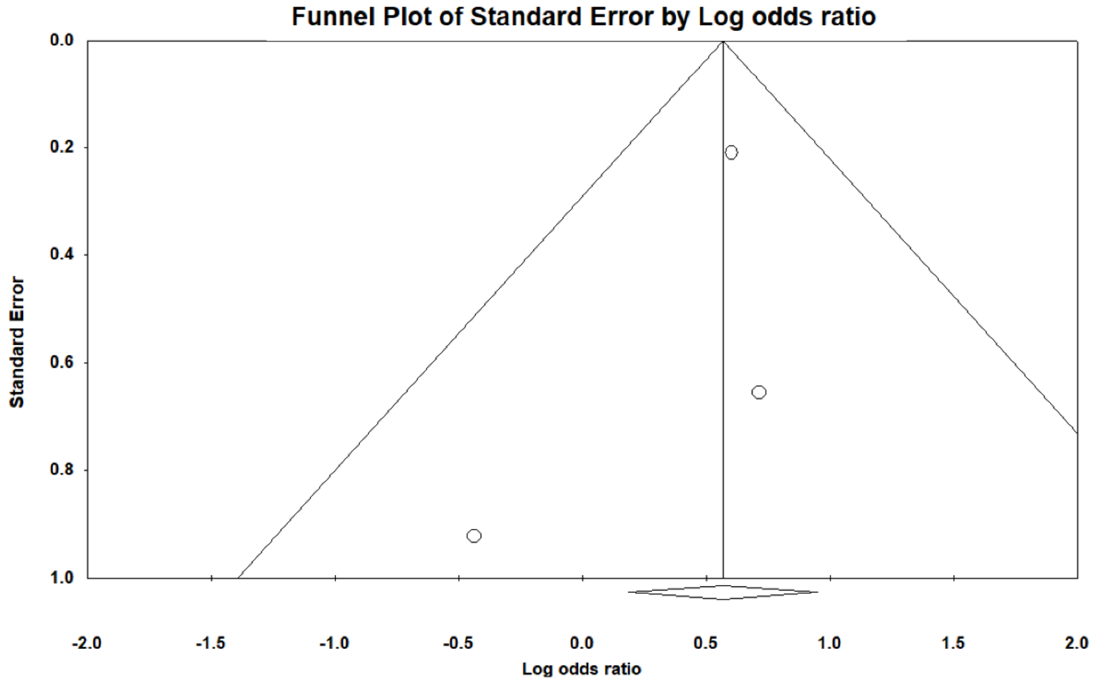

Fig S6. Meta-analysis A. Forest plot, B. Funnel plot for the Cough as a side effect of different COVID 19 vaccine in phase 1/2/3 RCT.

A

Meta Analysis

Group by Vaccine type	Study name	Odds ratio	Lower limit	Upper limit	Z-Value	p-Value	Vaccine	Placebo
		Statistics for each study					Events / Total	
Adeno vector	Zhu et al.	4.556	2.377	8.731	4.569	0.000	82 / 253	12 / 126
Adeno vector	Zhu1 et al.	1.847	0.867	3.936	1.590	0.112	21 / 129	12 / 126
Adeno vector	Logunov2 et al.	0.727	0.461	1.146	-1.373	0.170	60 / 14964	27 / 4902
Adeno vector		1.797	0.570	5.668	1.000	0.317	163 / 15346	51 / 5154
Inactivated	Xia.1 et al.	0.659	0.057	7.552	-0.336	0.737	2 / 84	1 / 28
Inactivated	Xia.2 et al.	0.243	0.015	4.014	-0.988	0.323	1 / 112	1 / 28
Inactivated	Xia.4 et al.	0.743	0.074	7.427	-0.253	0.800	3 / 112	1 / 28
Inactivated	Xia.5 et al.	0.152	0.024	0.955	-2.009	0.045	2 / 112	3 / 28
Inactivated	Xia3 et al.	0.097	0.008	1.245	-1.792	0.073	1 / 32	2 / 8
Inactivated		0.270	0.096	0.761	-2.478	0.013	9 / 452	8 / 120
Pro-Subunit	Yang et al.	1.352	0.458	3.996	0.546	0.585	8 / 150	6 / 150
Pro-Subunit	Yang1 et al.	1.899	0.684	5.276	1.231	0.218	11 / 150	6 / 150
Pro-Subunit	Yang2 et al.	1.000	0.434	2.303	0.000	1.000	12 / 150	12 / 150
Pro-Subunit	Yang3 et al.	0.910	0.388	2.132	-0.217	0.828	11 / 150	12 / 150
Pro-Subunit		1.174	0.737	1.869	0.676	0.499	42 / 600	36 / 600
RNA	Chu et al.	11.000	1.381	87.641	2.265	0.024	10 / 100	1 / 100
RNA	Chu2 et al.	21.732	2.840	166.268	2.966	0.003	18 / 100	1 / 100
RNA	Baden et al.	61.745	45.930	83.005	27.311	0.000	2356 / 15181	45 / 15170
RNA		36.905	12.945	105.216	6.751	0.000	2384 / 15381	47 / 15370
Overall		1.568	1.081	2.275	2.371	0.018	2598 / 31779	142 / 21244

Odds ratio and 95% CI

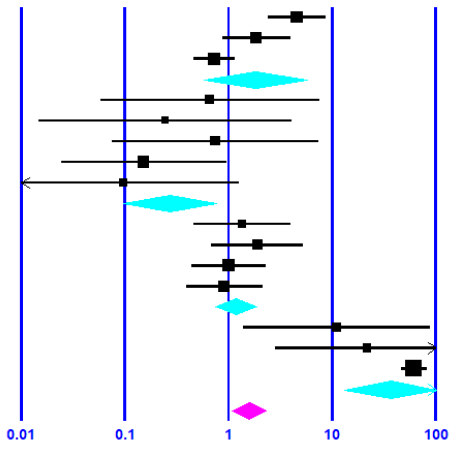

Groups		Effect size and 95% interval			Test of null (2-Tail)		Heterogeneity			
Group	Number Studies	Point estimate	Lower limit	Upper limit	Z-value	P-value	Q-value	df (Q)	P-value	I-squared
**Mixed effects analysis**										
Adeno vector	3	1.797	0.570	5.668	1.000	0.317	21.109	2	0.000	90.525
Inactivated	5	0.270	0.096	0.761	-2.478	0.013	2.259	4	0.688	0.000
Pro-Subunit	4	1.174	0.737	1.869	0.676	0.499	1.403	3	0.705	0.000
RNA	3	36.905	12.945	105.216	6.751	0.000	3.528	2	0.171	43.317

B

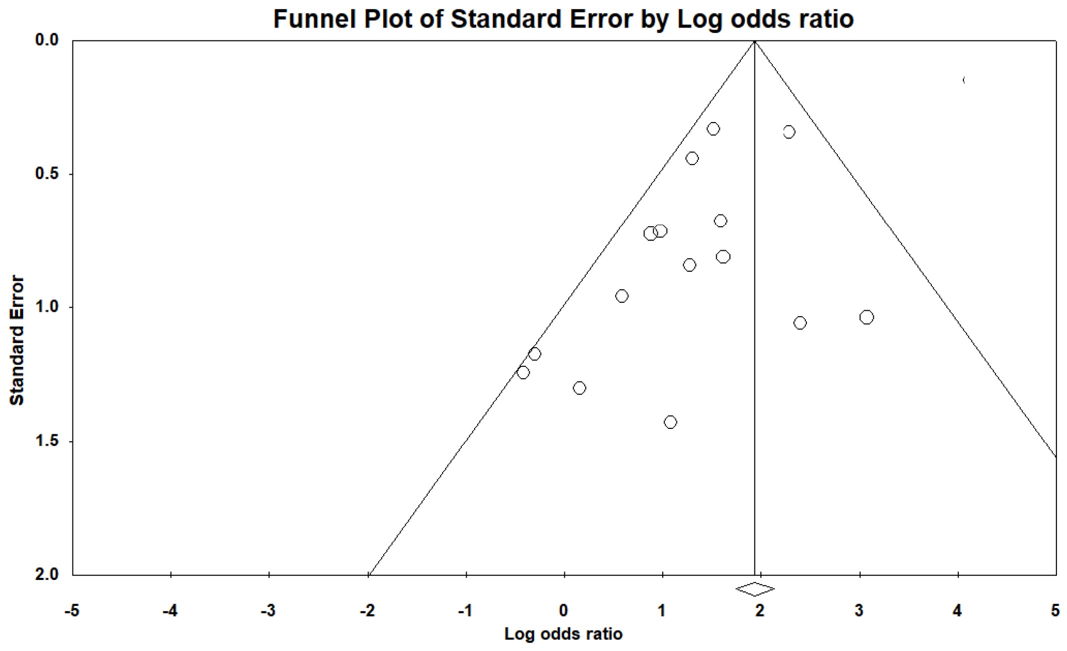

Fig S7. Meta-analysis A. Forest plot, B. Funnel plot for the Fever as a side effect of different COVID 19 vaccine in phase 1/2/3 RCT.

A

Meta Analysis

Group by Vaccine type	Study name	Odds ratio	Lower limit	Upper limit	Z-Value	p-Value	Vaccine	Placebo
		Statistics for each study					Events / Total	
Adeno vector	Folegatti1 et al.	3.019	2.354	3.872	8.699	0.000	365 / 543	216 / 534
Adeno vector		3.019	2.354	3.872	8.699	0.000		
Pro-Subunit	Richmond1 et al.	5.667	0.432	74.379	1.321	0.187	2 / 8	1 / 18
Pro-Subunit	Richmond3 et al.	2.429	0.133	44.501	0.598	0.550	1 / 8	1 / 18
Pro-Subunit	Richmond4 et al.	0.714	0.054	9.497	-0.255	0.799	1 / 8	2 / 12
Pro-Subunit	Richmond6 et al.	5.667	0.432	74.379	1.321	0.187	2 / 8	1 / 18
Pro-Subunit	Richmond7 et al.	3.000	0.372	24.171	1.032	0.302	3 / 8	2 / 12
Pro-Subunit	Richmond9 et al.	0.714	0.054	9.497	-0.255	0.799	1 / 8	2 / 12
Pro-Subunit	Richmond10 et al.	2.429	0.133	44.501	0.598	0.550	1 / 8	1 / 18
Pro-Subunit	Richmond11 et al.	5.667	0.432	74.379	1.321	0.187	2 / 8	1 / 18
Pro-Subunit	Richmond12 et al.	0.714	0.054	9.497	-0.255	0.799	1 / 8	2 / 12
Pro-Subunit	Richmond13 et al.	5.667	0.432	74.379	1.321	0.187	2 / 8	1 / 18
Pro-Subunit	Keech9 et al.	1.333	0.381	4.672	0.450	0.653	8 / 25	6 / 23
Pro-Subunit	Keech10 et al.	2.429	0.725	8.133	1.439	0.150	12 / 26	6 / 23
Pro-Subunit	Keech11 et al.	3.606	1.064	12.222	2.060	0.039	14 / 25	6 / 23
Pro-Subunit	Keech12 et al.	0.675	0.175	2.597	-0.572	0.567	5 / 26	6 / 23
Pro-Subunit		1.990	1.211	3.268	2.717	0.007		
RNA	Kremsner et al.	1.224	0.422	3.549	0.373	0.709	12 / 47	7 / 32
RNA	Kremsner1 et al.	4.221	1.534	11.616	2.788	0.005	26 / 48	7 / 32
RNA	Kremsner2 et al.	5.952	2.143	16.535	3.422	0.001	30 / 48	7 / 32
RNA	Kremsner3 et al.	9.821	3.378	28.554	4.196	0.000	33 / 45	7 / 32
RNA	Kremsner4 et al.	13.095	3.821	44.882	4.093	0.000	22 / 28	7 / 32
RNA		5.130	2.326	11.314	4.052	0.000		
Overall		2.904	2.344	3.598	9.755	0.000		

Odds ratio and 95% CI

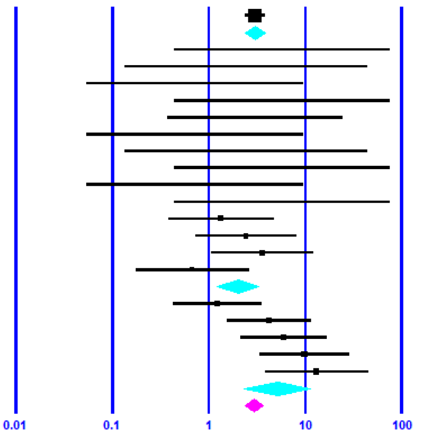

Group	Number Studies	Point estimate	Lower limit	Upper limit	Z-value	P-value	Q-value	df (Q)	P-value	I-squared
Groups	Effect size and 95% interval				Test of null (2-Tail)		Heterogeneity			
Mixed effects analysis										
Adeno vector	1	3.019	2.354	3.872	8.699	0.000	0.000	0	1.000	0.000
Pro-Subunit	14	1.990	1.211	3.268	2.717	0.007	8.411	13	0.816	0.000
RNA	5	5.130	2.326	11.314	4.052	0.000	10.819	4	0.029	63.027

B

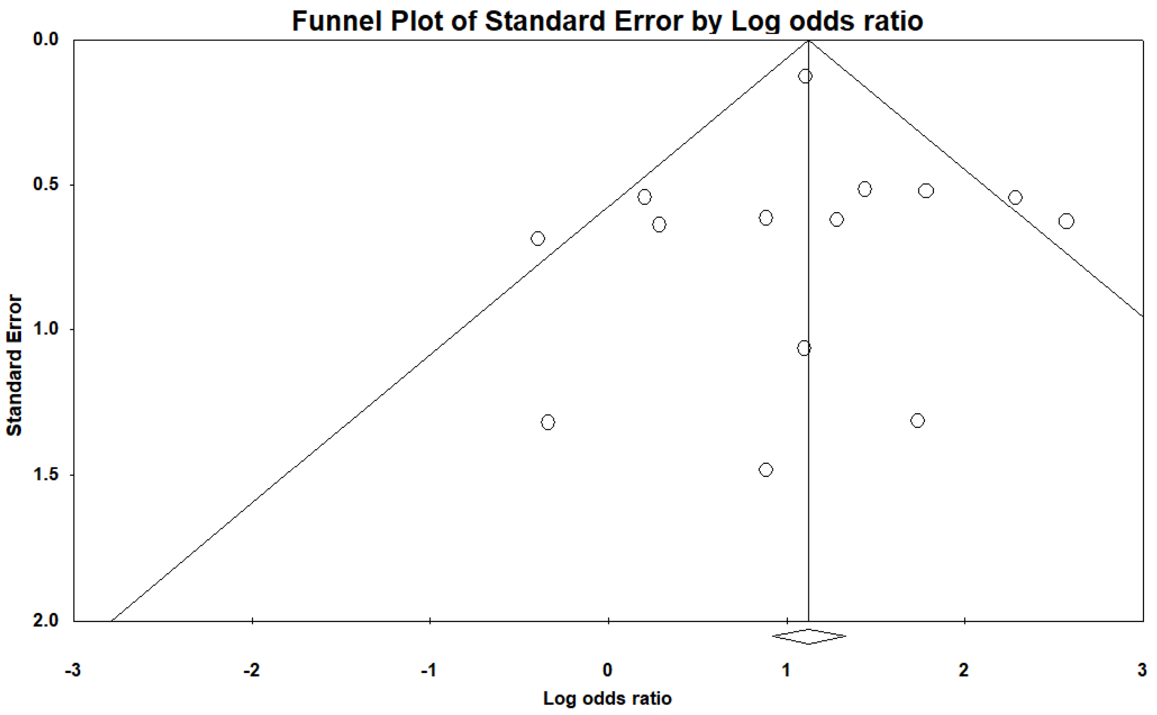

Fig S8. Meta-analysis A. Forest plot, B. Funnel plot for the Headache as a side effect of different COVID 19 vaccine in phase 1/2 RCT.

A

Meta Analysis

Group by Vaccine type	Study name	Odds ratio	Lower limit	Upper limit	Z-Value	p-Value	Vaccine	Placebo
		Statistics for each study					Events / Total	
Adeno vector	Zhu et al.	2.600	1.457	4.639	3.235	0.001	73 / 253	17 / 126
Adeno vector	Zhu1 et al.	2.482	1.309	4.706	2.785	0.005	36 / 129	17 / 126
Adeno vector		2.546	1.658	3.911	4.268	0.000	109 / 382	34 / 252
Inactivated	Xia et al.	0.325	0.020	5.380	-0.785	0.433	1 / 84	1 / 28
Inactivated	Xia4 et al.	0.117	0.010	1.341	-1.724	0.085	1 / 112	2 / 28
Inactivated		0.182	0.029	1.145	-1.816	0.069	2 / 196	3 / 56
Pro-Subunit	Yang et al.	1.510	0.249	9.170	0.448	0.654	3 / 150	2 / 150
Pro-Subunit	Yang1 et al.	1.000	0.139	7.193	0.000	1.000	2 / 150	2 / 150
Pro-Subunit		1.252	0.331	4.739	0.331	0.741	5 / 300	4 / 300
RNA	Chu et al.	1.519	0.768	3.003	1.200	0.230	25 / 100	18 / 100
RNA	Chu1 et al.	3.692	1.971	6.917	4.079	0.000	48 / 100	20 / 100
RNA	Chu2 et al.	1.211	0.601	2.442	0.535	0.593	21 / 100	18 / 100
RNA	Chu3 et al.	4.000	2.136	7.492	4.330	0.000	50 / 100	20 / 100
RNA		2.320	1.282	4.199	2.780	0.005	144 / 400	76 / 400
Overall		2.174	1.561	3.026	4.597	0.000	260 / 1278	117 / 1008

Odds ratio and 95% CI

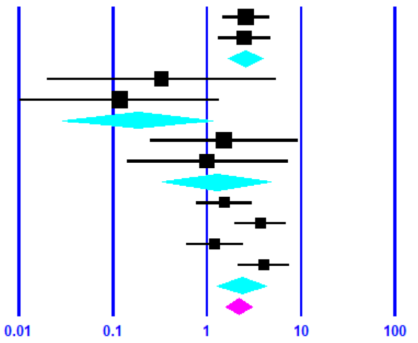

Group	Number Studies	Point estimate	Lower limit	Upper limit	Z-value	P-value	Q-value	df (Q)	P-value	I-squared
Groups	Effect size and 95% interval				Test of null (2-Tail)		Heterogeneity			
**Mixed effects analysis**										
Adeno vector	2	2.546	1.658	3.911	4.268	0.000	0.011	1	0.916	0.000
Inactivated	2	0.182	0.029	1.145	-1.816	0.069	0.290	1	0.590	0.000
Pro-Subunit	2	1.252	0.331	4.739	0.331	0.741	0.091	1	0.762	0.000
RNA	4	2.320	1.282	4.199	2.780	0.005	9.740	3	0.021	69.200

B

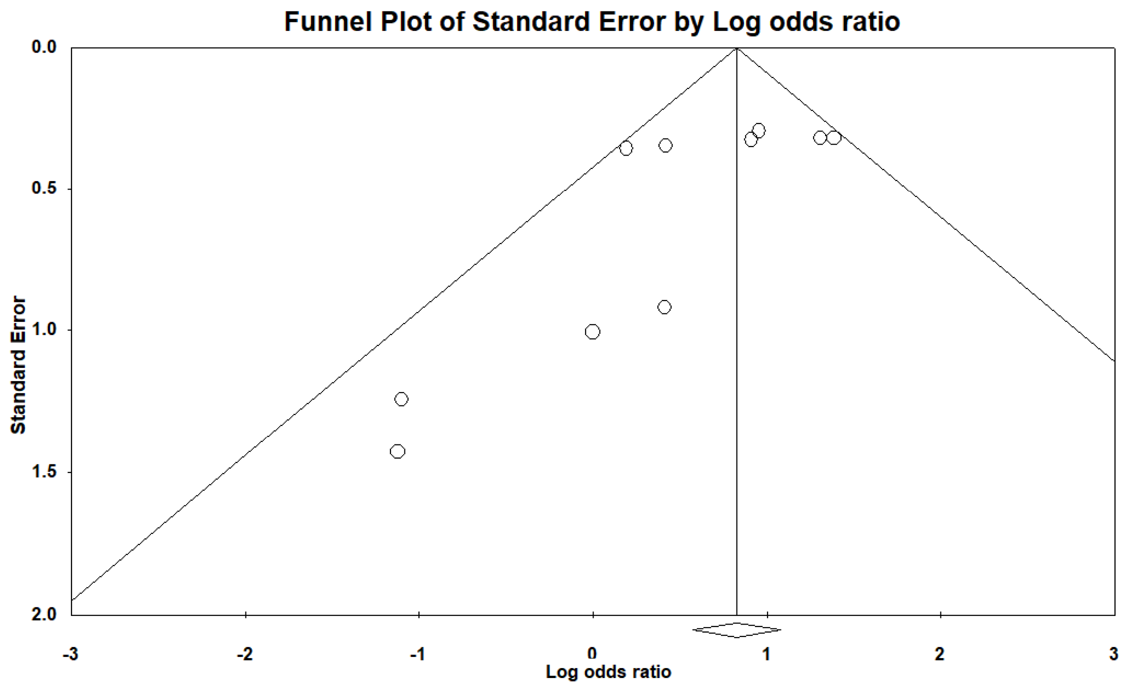

Fig S9. Meta-analysis A. Forest plot, B. Funnel plot for the Headache as a side effect of different COVID 19 vaccine in phase 2 RCT.

Meta Analysis

Group by Vaccine type	Study name	Odds ratio	Lower limit	Upper limit	Z-Value	p-Value	Vaccine	Placebo
Adenoviral vector (rAd26-S+rAd5-S)	Logunov et al.	0.585	0.498	0.688	-6.500	0.000	434 / 14964	238 / 4902
Adenoviral vector (rAd26-S+rAd5-S)		0.585	0.498	0.688	-6.500	0.000	434 / 14964	238 / 4902
RNA	Baden et al.	4.633	4.409	4.868	60.639	0.000	8897 / 15181	3551 / 15170
RNA		4.633	4.409	4.868	60.639	0.000	8897 / 15181	3551 / 15170
Overall		3.877	3.698	4.065	56.065	0.000	9331 / 30145	3789 / 20072

Statistics for each study; Events / Total; Odds ratio and 95% CI

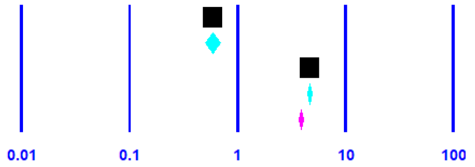

Groups		Effect size and 95% interval				Test of null (2-Tail)		Heterogeneity			
Group	Number Studies	Point estimate	Lower limit	Upper limit		Z-value	P-value	Q-value	df (Q)	P-value	I-squared
Mixed effects analysis											
Adenoviral	1	0.585	0.498	0.688		-6.500	0.000	0.000	0	1.000	0.000
RNA	1	4.633	4.409	4.868		60.639	0.000	0.000	0	1.000	0.000

Fig S10. Meta-analysis A. Forest plot, B. Funnel plot for the Headache as a side effect of different COVID 19 vaccine in phase 3 RCT.

A

Meta Analysis

Group by Vaccine type	Study name	Odds ratio	Lower limit	Upper limit	Z-Value	p-Value	Vaccine	Placebo
		Statistics for each study					Events / Total	
Adeno vector	Zhu et al.	3.605	2.120	6.131	4.734	0.000	106 / 253	21 / 126
Adeno vector	Zhu1 et al.	2.588	1.430	4.685	3.142	0.002	44 / 129	21 / 126
Adeno vector	Folegatti1 et al.	2.589	2.016	3.325	7.457	0.000	380 / 543	253 / 534
Adeno vector		2.729	2.209	3.371	9.308	0.000	530 / 925	295 / 786
Inactivated	Xia.3 et al.	0.491	0.043	5.616	-0.572	0.567	2 / 112	1 / 28
Inactivated	Xia.4 et al.	0.075	0.007	0.752	-2.202	0.028	1 / 112	3 / 28
Inactivated	Xia.5 et al.	0.243	0.015	4.014	-0.988	0.323	1 / 112	1 / 28
Inactivated	Zhang. et al.	0.322	0.052	1.981	-1.222	0.222	2 / 120	3 / 60
Inactivated	Zhang.2 et al.	0.322	0.052	1.981	-1.222	0.222	2 / 120	3 / 60
Inactivated	Zhang et al.	0.455	0.075	2.756	-0.857	0.391	2 / 24	4 / 24
Inactivated	Zhang2 et al.	1.000	0.219	4.564	0.000	1.000	4 / 24	4 / 24
Inactivated		0.392	0.187	0.820	-2.486	0.013	14 / 624	19 / 252
Pro-Subunit	Keech9 et al.	0.909	0.164	5.032	-0.109	0.913	3 / 25	3 / 23
Pro-Subunit	Keech10 et al.	5.714	1.357	24.064	2.376	0.017	12 / 26	3 / 23
Pro-Subunit	Keech11 et al.	6.154	1.451	26.105	2.465	0.014	12 / 25	3 / 23
Pro-Subunit	Keech12 et al.	1.212	0.241	6.093	0.233	0.815	4 / 26	3 / 23
Pro-Subunit		2.702	1.019	7.165	1.998	0.046	31 / 102	12 / 92
RNA	Chu et al.	3.273	1.745	6.137	3.696	0.000	45 / 100	20 / 100
RNA	Chu1 et al.	5.785	3.106	10.775	5.531	0.000	62 / 100	22 / 100
RNA	Chu2 et al.	7.429	3.919	14.081	6.146	0.000	65 / 100	20 / 100
RNA	Chu3 et al.	6.584	3.518	12.322	5.895	0.000	65 / 100	22 / 100
RNA	Kremsner et al.	2.065	0.655	6.511	1.237	0.216	13 / 47	5 / 32
RNA	Kremsner1 et al.	5.870	1.935	17.804	3.126	0.002	25 / 48	5 / 32
RNA	Kremsner2 et al.	7.560	2.483	23.020	3.561	0.000	28 / 48	5 / 32
RNA	Kremsner3 et al.	16.691	5.172	53.867	4.709	0.000	34 / 45	5 / 32
RNA	Kremsner4 et al.	45.000	9.732	208.077	4.872	0.000	25 / 28	5 / 32
RNA	Walsh5 et al.	6.000	1.018	35.374	1.979	0.048	8 / 12	3 / 12
RNA	Walsh6 et al.	1.167	0.151	9.006	0.148	0.882	3 / 12	2 / 9
RNA	Walsh7 et al.	15.000	2.024	111.174	2.650	0.008	10 / 12	3 / 12
RNA	Walsh8 et al.	4.900	0.700	34.300	1.601	0.109	7 / 12	2 / 9
RNA	Walsh9 et al.	15.000	2.024	111.174	2.650	0.008	10 / 12	3 / 12
RNA	Walsh10 et al.	7.000	0.969	50.567	1.929	0.054	8 / 12	2 / 9
RNA	Walsh11 et al.	0.400	0.067	2.372	-1.009	0.313	4 / 12	5 / 9
RNA	Walsh12 et al.	0.318	0.024	4.202	-0.870	0.384	1 / 12	2 / 9
RNA	Walsh13 et al.	1.120	0.196	6.414	0.127	0.899	7 / 12	5 / 9
RNA	Walsh14 et al.	1.750	0.242	12.642	0.555	0.579	4 / 12	2 / 9
RNA	Walsh15 et al.	2.400	0.376	15.319	0.926	0.355	9 / 12	5 / 9
RNA	Walsh16 et al.	1.167	0.151	9.006	0.148	0.882	3 / 12	2 / 9
RNA	Mulligan et al.	7.000	0.969	50.567	1.929	0.054	8 / 12	2 / 9
RNA	Mulligan1 et al.	17.500	1.968	155.592	2.567	0.010	10 / 12	2 / 9
RNA	Mulligan2 et al.	17.500	1.968	155.592	2.567	0.010	10 / 12	2 / 9
RNA		5.008	3.422	7.330	8.290	0.000	464 / 796	151 / 704
Overall		2.782	2.332	3.318	11.371	0.000	1039 / 2447	477 / 1834

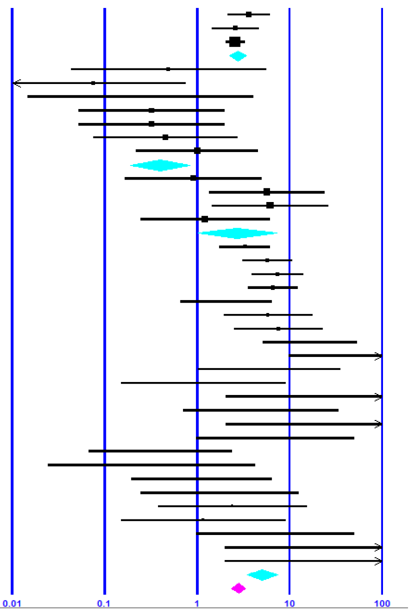

Group	Number Studies	Point estimate	Lower limit	Upper limit	Z-value	P-value	Q-value	df (Q)	P-value	I-squared
Groups		Effect size and 95% interval			Test of null (2-Tail)		Heterogeneity			
**Mixed effects analysis**										
Adeno vector	3	2.729	2.209	3.371	9.308	0.000	1.257	2	0.533	0.000
Inactivated	7	0.392	0.187	0.820	-2.486	0.013	3.698	6	0.718	0.000
Pro-Subunit	4	2.702	1.019	7.165	1.998	0.046	4.779	3	0.189	37.224
RNA	24	5.008	3.422	7.330	8.290	0.000	44.427	23	0.005	48.230

B

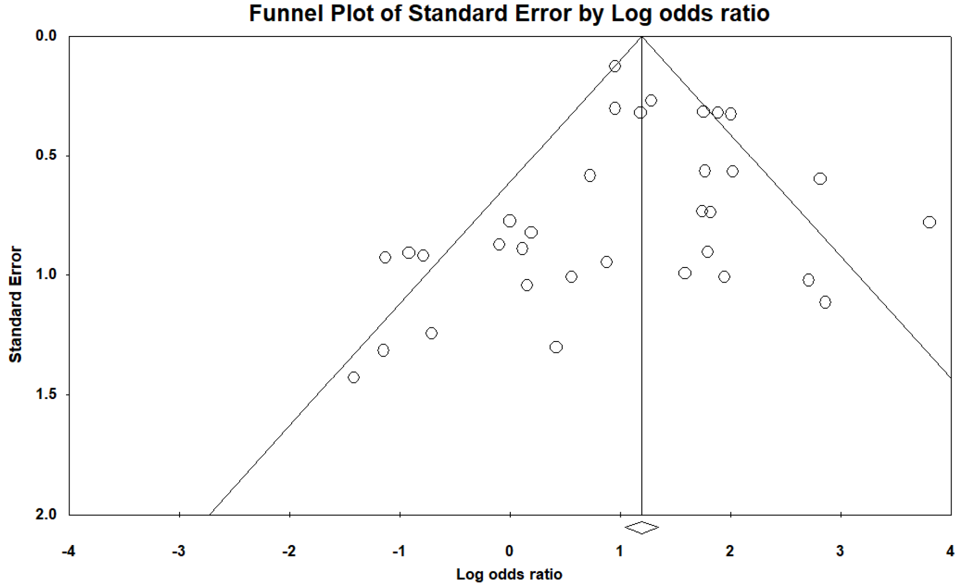

Fig S11. Meta-analysis A. Forest plot, B. Funnel plot for the Fatigue as a side effect of different COVID 19 vaccine in phase 1/2 RCT.

Meta Analysis

Group by Phase	Study name	Odds ratio	Lower limit	Upper limit	Z-Value	p-Value	Vaccine	Placebo
		Statistics for each study					Events / Total	
2-3	Polack et al.	4.870	4.653	5.098	68.080	0.000	10490 / 18860	3857 / 18846
2-3		4.870	4.653	5.098	68.080	0.000		
3	Baden et al.	6.169	5.867	6.488	70.891	0.000	9911 / 15181	3544 / 15170
3		6.169	5.867	6.488	70.891	0.000		
Overall		5.418	5.238	5.604	98.050	0.000		

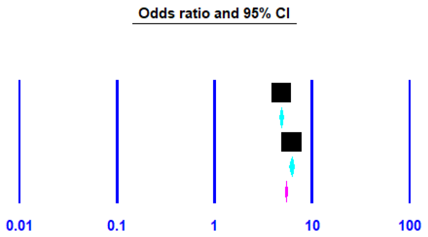

Group	Number Studies	Point estimate	Lower limit	Upper limit	Z-value	P-value	Q-value	df (Q)	P-value	I-squared
Groups		Effect size and 95% interval			Test of null (2-Tail)		Heterogeneity			
Mixed effects analysis										
2-3	1	4.870	4.653	5.098	68.080	0.000	0.000	0	1.000	0.000
3	1	6.169	5.867	6.488	70.891	0.000	0.000	0	1.000	0.000

Fig S12. Meta-analysis A. Forest plot, B. Funnel plot for the Fatigue as a side effect of different COVID 19 vaccine in both mRNA-based vaccine, in RCT 2/3, and 3.

A

Meta Analysis

Group by Vaccine type	Study name	Odds ratio	Lower limit	Upper limit	Z-Value	p-Value	Vaccine (Events / Total)	Placebo (Events / Total)
Adeno vector	Zhu et al.	0.249	0.118	0.525	-3.656	0.000	12 / 253	21 / 126
Adeno vector	Zhu1 et al.	0.079	0.018	0.344	-3.381	0.001	2 / 129	21 / 126
Adeno vector		0.168	0.058	0.490	-3.267	0.001	14 / 382	42 / 252
Inactivated	Zhang. et al.	0.160	0.016	1.569	-1.574	0.116	1 / 120	3 / 60
Inactivated	Zhang.2 et al.	0.160	0.016	1.569	-1.574	0.116	1 / 120	3 / 60
Inactivated	Zhang et al.	0.217	0.022	2.108	-1.317	0.188	1 / 24	4 / 24
Inactivated	Zhang2 et al.	0.217	0.022	2.108	-1.317	0.188	1 / 24	4 / 24
Inactivated		0.186	0.060	0.583	-2.889	0.004	4 / 288	14 / 168
Pro-Subunit	Yang2 et al.	2.985	1.047	8.510	2.046	0.041	14 / 150	5 / 150
Pro-Subunit	Yang3 et al.	2.295	0.777	6.774	1.504	0.133	11 / 150	5 / 150
Pro-Subunit		2.629	1.238	5.580	2.516	0.012	25 / 300	10 / 300
RNA	Mulligan2 et al.	17.500	1.968	155.592	2.567	0.010	10 / 12	2 / 9
RNA		17.500	1.968	155.592	2.567	0.010	10 / 12	2 / 9
Overall		0.860	0.508	1.454	-0.564	0.573	53 / 982	68 / 729

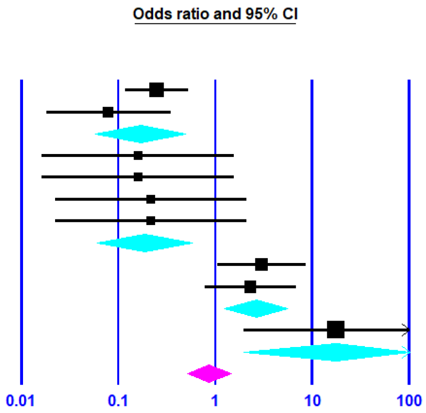

Groups	Effect size and 95% interval				Test of null (2-Tail)		Heterogeneity			
Group	Number Studies	Point estimate	Lower limit	Upper limit	Z-value	P-value	Q-value	df (Q)	P-value	I-squared
**Mixed effects analysis**										
Adeno vector	2	0.168	0.058	0.490	-3.267	0.001	1.867	1	0.172	46.448
Inactivated	4	0.186	0.060	0.583	-2.889	0.004	0.070	3	0.995	0.000
Pro-Subunit	2	2.629	1.238	5.580	2.516	0.012	0.117	1	0.732	0.000
RNA	1	17.500	1.968	155.592	2.567	0.010	0.000	0	1.000	0.000

B

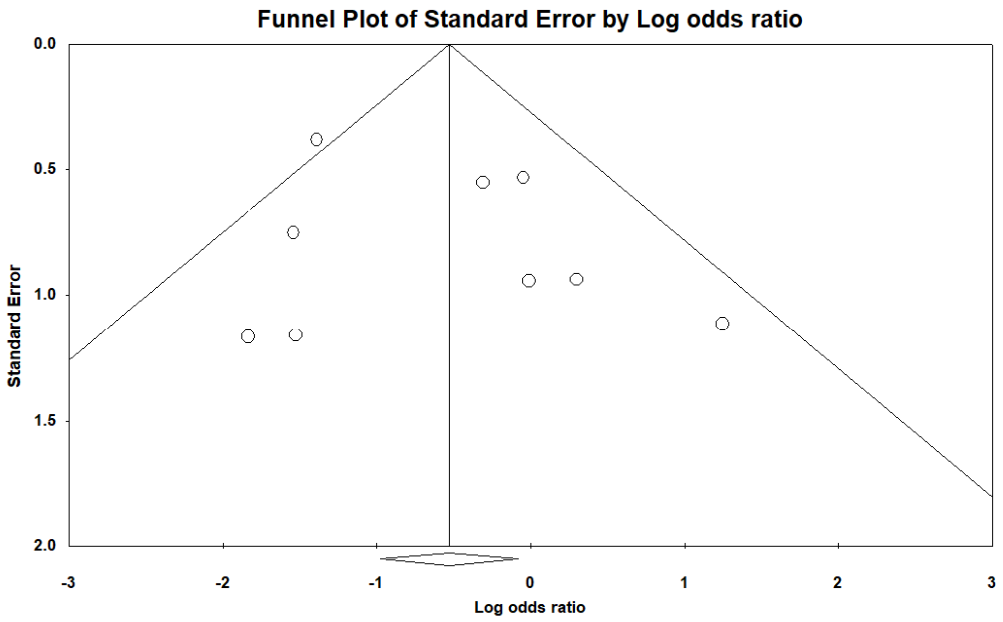

Fig S13. Meta-analysis A. Forest plot, B. Funnel plot for the Induration as a side effect of different COVID 19 vaccine in RCT 1/2.

A

Meta Analysis

Group by Vaccine type	Study name	Odds ratio	Lower limit	Upper limit	Z-Value	p-Value
Adeno vector	Zhu et al.	2.008	0.222	18.155	0.621	0.535
Adeno vector	Zhu1 et al.	0.977	0.060	15.784	-0.017	0.987
Adeno vector	Folegatti1 et al.	2.824	2.031	3.927	6.175	0.000
Adeno vector		2.764	1.999	3.820	6.155	0.000
Inactivated	Xia.1 et al.	0.325	0.020	5.380	-0.785	0.433
Inactivated		0.325	0.020	5.380	-0.785	0.433
RNA	Chu et al.	11.494	2.600	50.819	3.220	0.001
RNA	Chu2 et al.	19.056	4.397	82.583	3.939	0.000
RNA	Kremsner et al.	0.674	0.041	11.182	-0.275	0.783
RNA	Kremsner1 et al.	4.429	0.507	38.681	1.346	0.178
RNA	Kremsner2 et al.	4.429	0.507	38.681	1.346	0.178
RNA	Kremsner3 et al.	6.703	0.794	56.568	1.748	0.080
RNA	Kremsner4 et al.	10.333	1.183	90.256	2.112	0.035
RNA	Mulligan2 et al.	17.500	1.968	155.592	2.567	0.010
RNA		8.719	4.382	17.349	6.169	0.000
Overall		3.318	2.480	4.440	8.069	0.000

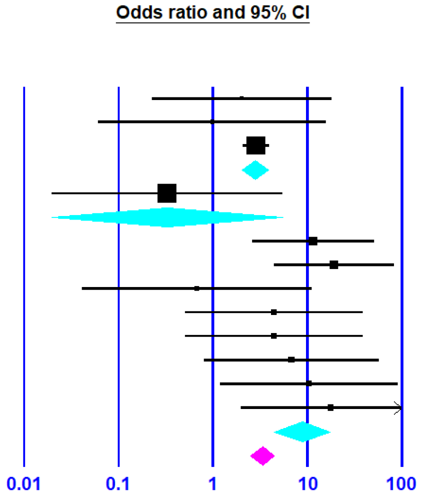

Groups		Effect size and 95% interval			Test of null (2-Tail)		Heterogeneity			
Group	Number Studies	Point estimate	Lower limit	Upper limit	Z-value	P-value	Q-value	df (Q)	P-value	I-squared
Mixed effects analysis										
Adeno vector	3	2.764	1.999	3.820	6.155	0.000	0.634	2	0.728	0.000
Inactivated	1	0.325	0.020	5.380	-0.785	0.433	0.000	0	1.000	0.000
RNA	8	8.719	4.382	17.349	6.169	0.000	5.639	7	0.582	0.000

B

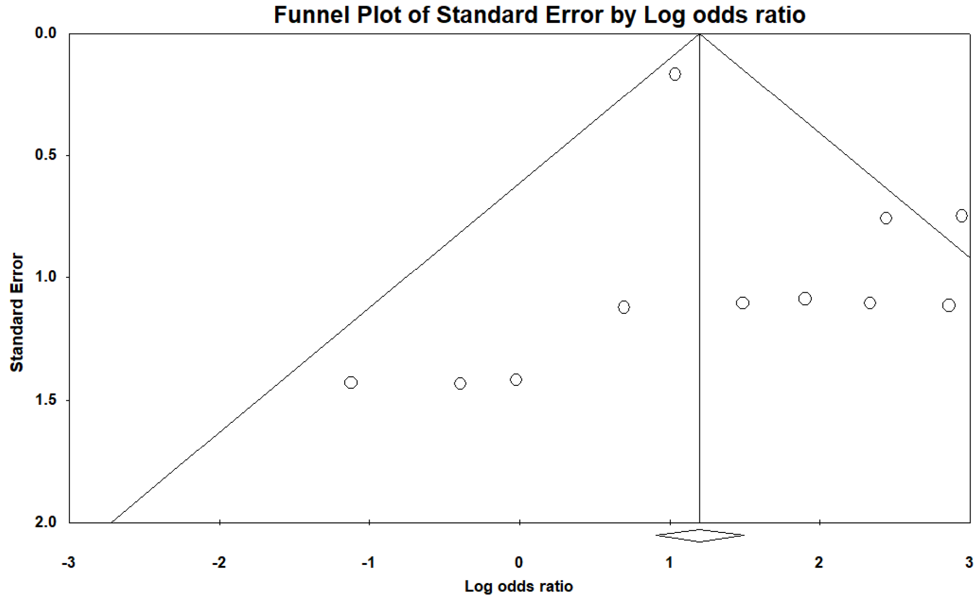

Fig S14. Meta-analysis A. Forest plot, B. Funnel plot for the Vomiting as a side effect of different COVID 19 vaccine in RCT 1/2.

Meta Analysis

Group by Phase	Study name	Statistics for each study: Odds ratio	Lower limit	Upper limit	Z-Value	p-Value	Events / Total: Vaccine	Placebo
2-3	Polack et al.	4.870	4.653	5.098	68.080	0.000	10490 / 18860	3857 / 18846
2-3		4.870	4.653	5.098	68.080	0.000	10490 / 18860	3857 / 18846
3.00	Baden et al.	6.169	5.867	6.488	70.891	0.000	9911 / 15181	3544 / 15170
3.00		6.169	5.867	6.488	70.891	0.000	9911 / 15181	3544 / 15170
Overall		5.418	5.238	5.604	98.050	0.000	20401 / 34041	7401 / 34016

Odds ratio and 95% CI

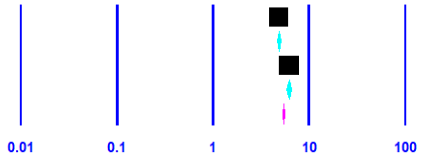

Groups		Effect size and 95% interval			Test of null (2-Tail)		Heterogeneity			
Group	Number Studies	Point estimate	Lower limit	Upper limit	Z-value	P-value	Q-value	df (Q)	P-value	I-squared
Mixed effects analysis										
2-3	1	4.870	4.653	5.098	68.080	0.000	0.000	0	1.000	0.000
3	1	6.169	5.867	6.488	70.891	0.000	0.000	0	1.000	0.000

Fig S15. Meta-analysis A. Forest plot, B. Funnel plot for the Vomiting as a side effect of different COVID 19 vaccine in both mRNA-based vaccine, in RCT 2/3, and 3.

A

Meta Analysis

Group by Vaccine type	Study name	Odds ratio	Lower limit	Upper limit	Z-Value	p-Value	Vaccine	Placebo
		Statistics for each study					Events / Total	
Adeno vector	Zhu et al.	2.476	0.824	7.441	1.616	0.106	19 / 253	4 / 126
Adeno vector	Zhu1 et al.	2.563	0.782	8.397	1.554	0.120	10 / 129	4 / 126
Adeno vector		2.516	1.123	5.638	2.242	0.025	29 / 382	8 / 252
Inactivated	Xia.3 et al.	0.491	0.043	5.616	-0.572	0.567	2 / 112	1 / 28
Inactivated	Zhang. et al.	0.496	0.030	8.067	-0.493	0.622	1 / 120	1 / 60
Inactivated	Zhang et al.	1.000	0.059	16.970	0.000	1.000	1 / 24	1 / 24
Inactivated		0.608	0.130	2.835	-0.634	0.526	4 / 256	3 / 112
RNA	Kremsner1 et al.	0.235	0.043	1.295	-1.664	0.096	2 / 48	5 / 32
RNA	Kremsner2 et al.	0.628	0.166	2.374	-0.686	0.493	5 / 48	5 / 32
RNA	Kremsner3 et al.	0.386	0.085	1.747	-1.236	0.217	3 / 45	5 / 32
RNA	Kremsner4 et al.	0.648	0.140	2.996	-0.555	0.579	3 / 28	5 / 32
RNA	Mulligan2 et al.	1.750	0.242	12.642	0.555	0.579	4 / 12	2 / 9
RNA		0.548	0.272	1.106	-1.678	0.093	17 / 181	22 / 137
Overall		0.997	0.604	1.645	-0.012	0.991	50 / 819	33 / 501

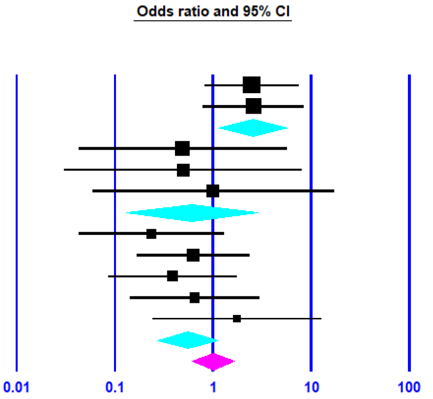

Group	Number Studies	Point estimate	Lower limit	Upper limit	Z-value	P-value	Q-value	df (Q)	P-value	I-squared
Groups		Effect size and 95% interval			Test of null (2-Tail)		Heterogeneity			
Mixed effects analysis										
Adeno vector	2	2.516	1.123	5.638	2.242	0.025	0.002	1	0.967	0.000
Inactivated	3	0.608	0.130	2.835	-0.634	0.526	0.169	2	0.919	0.000
RNA	5	0.548	0.272	1.106	-1.678	0.093	2.565	4	0.633	0.000

B

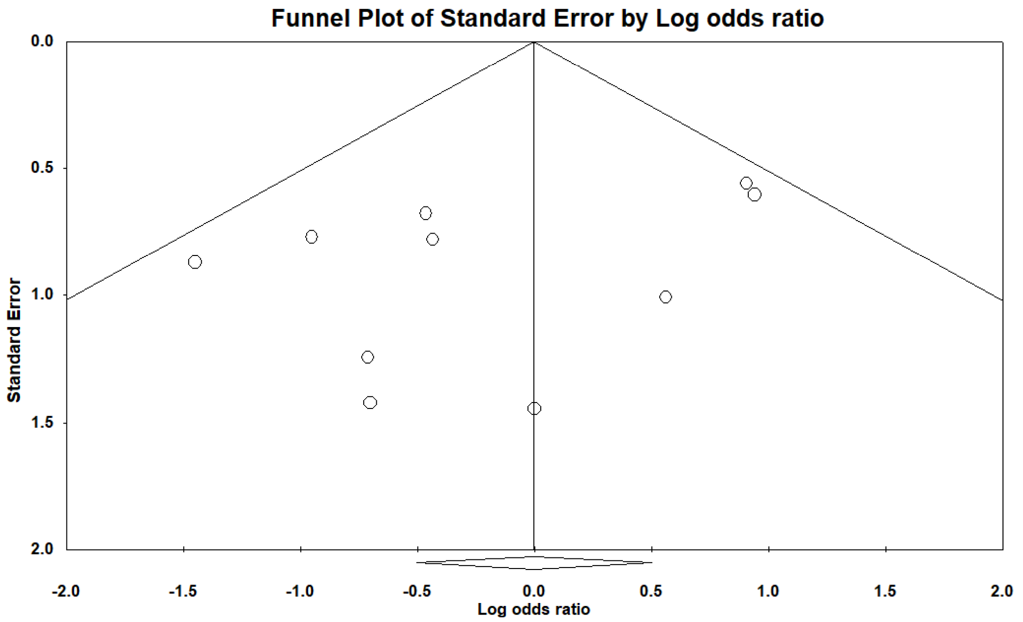

Fig S16. Meta-analysis A. Forest plot, B. Funnel plot for the Diarrhea as a side effect of different COVID 19 vaccine in RCT 1/2.

A

Meta Analysis

Group by Vaccine type	Study name	Odds ratio	Lower limit	Upper limit	Z-Value	p-Value	Vaccine	Placebo
Adeno vector	Zhu et al.	7.472	2.262	24.686	3.298	0.001	39 / 253	3 / 126
Adeno vector	Zhu1 et al.	8.896	2.598	30.462	3.480	0.001	23 / 129	3 / 126
Adeno vector	Folegatti1 et al.	4.360	3.361	5.656	11.088	0.000	321 / 543	133 / 534
Adeno vector		4.595	3.582	5.894	12.002	0.000	383 / 925	139 / 786
Inactivated	Zhang.2 et al.	1.000	0.089	11.254	0.000	1.000	2 / 120	1 / 60
Inactivated	Zhang2 et al.	2.091	0.177	24.734	0.585	0.558	2 / 24	1 / 24
Inactivated		1.435	0.255	8.088	0.410	0.682	4 / 144	2 / 84
Pro-Subunit	Richmond1 et al.	17.000	1.471	196.426	2.269	0.023	4 / 8	1 / 18
Pro-Subunit	Richmond3 et al.	2.429	0.133	44.501	0.598	0.550	1 / 8	1 / 18
Pro-Subunit	Richmond6 et al.	5.667	0.432	74.379	1.321	0.187	2 / 8	1 / 18
Pro-Subunit	Richmond11 et al.	28.333	2.389	336.008	2.650	0.008	5 / 8	1 / 18
Pro-Subunit	Keech9 et al.	0.580	0.088	3.825	-0.566	0.571	2 / 25	3 / 23
Pro-Subunit	Keech10 et al.	1.212	0.241	6.093	0.233	0.815	4 / 26	3 / 23
Pro-Subunit	Keech11 et al.	7.222	1.703	30.637	2.682	0.007	13 / 25	3 / 23
Pro-Subunit	Keech12 et al.	0.267	0.026	2.764	-1.108	0.268	1 / 26	3 / 23
Pro-Subunit		2.928	0.979	8.757	1.923	0.055	32 / 134	16 / 164
RNA	Chu et al.	11.819	4.732	29.519	5.288	0.000	43 / 100	6 / 100
RNA	Chu1 et al.	4.889	2.371	10.082	4.298	0.000	40 / 100	12 / 100
RNA	Chu2 et al.	22.545	9.016	56.372	6.663	0.000	59 / 100	6 / 100
RNA	Chu3 et al.	6.769	3.297	13.900	5.209	0.000	48 / 100	12 / 100
RNA	Kremsner et al.	5.425	0.634	46.440	1.544	0.123	7 / 47	1 / 32
RNA	Kremsner1 et al.	14.091	1.756	113.102	2.490	0.013	15 / 48	1 / 32
RNA	Kremsner2 et al.	28.520	3.598	226.079	3.172	0.002	23 / 48	1 / 32
RNA	Kremsner3 et al.	24.800	3.110	197.782	3.031	0.002	20 / 45	1 / 32
RNA	Kremsner4 et al.	65.444	7.673	558.165	3.823	0.000	19 / 28	1 / 32
RNA	Mulligan2 et al.	4.900	0.700	34.300	1.601	0.109	7 / 12	2 / 9
RNA		10.713	6.519	17.604	9.358	0.000	281 / 628	43 / 569
Overall		5.206	4.193	6.465	14.940	0.000	700 / 1831	200 / 1603

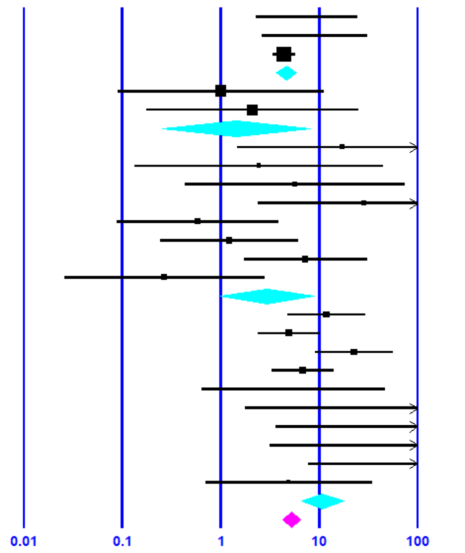

Groups		Effect size and 95% interval			Test of null (2-Tail)		Heterogeneity			
Group	Number Studies	Point estimate	Lower limit	Upper limit	Z-value	P-value	Q-value	df (Q)	P-value	I-squared
**Mixed effects analysis**										
Adeno vector	3	4.595	3.582	5.894	12.002	0.000	1.899	2	0.387	0.000
Inactivated	2	1.435	0.255	8.088	0.410	0.682	0.175	1	0.676	0.000
Pro-Subunit	8	2.928	0.979	8.757	1.923	0.055	14.992	7	0.036	53.307
RNA	10	10.713	6.519	17.604	9.358	0.000	13.583	9	0.138	33.741

B

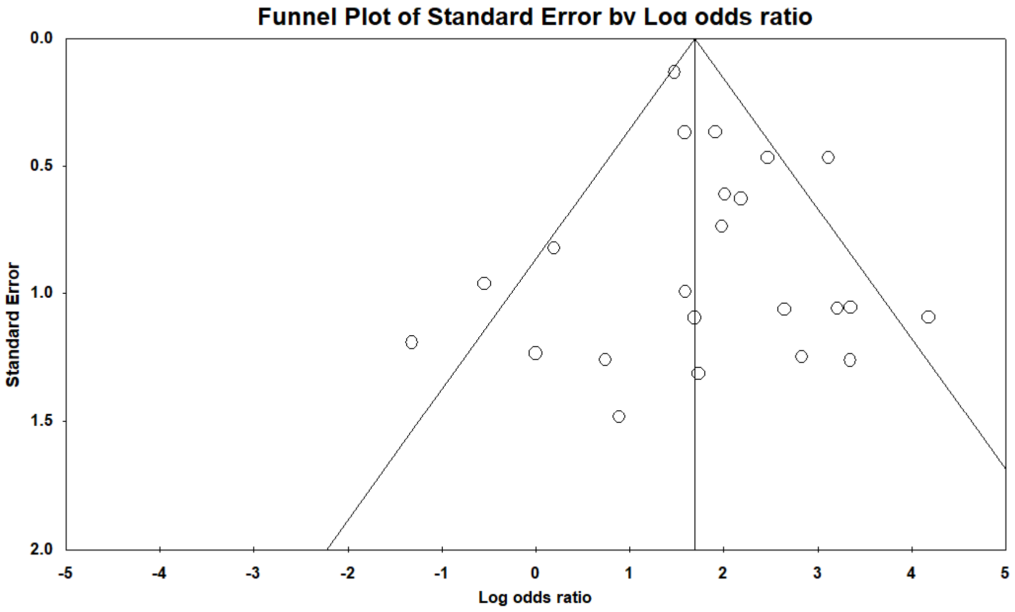

Fig S17. Meta-analysis A. Forest plot, B. Funnel plot for the Myalgia as a side effect of different COVID 19 vaccine in RCT 1/2.

Meta Analysis

Group by Phase	Study name	Odds ratio	Lower limit	Upper limit	Z-Value	p-Value	Vaccine	Placebo
		Statistics for each study					Events / Total	
2/3	Polack et al.	7.008	6.571	7.475	59.195	0.000	6340 / 18860	1270 / 18846
2/3		7.008	6.571	7.475	59.195	0.000	6340 / 18860	1270 / 18846
3.00	Baden et al.	9.725	9.177	10.306	76.839	0.000	8800 / 15181	1884 / 15170
3.00		9.725	9.177	10.306	76.839	0.000	8800 / 15181	1884 / 15170
Overall		8.399	8.044	8.769	96.713	0.000	15140 / 34041	3154 / 34016

Odds ratio and 95% CI

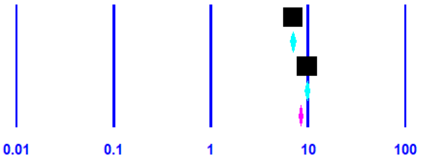

Groups	Effect size and 95% interval				Test of null (2-Tail)		Heterogeneity			
Group	Number Studies	Point estimate	Lower limit	Upper limit	Z-value	P-value	Q-value	df (Q)	P-value	I-squared
Mixed effects analysis										
2/3	1	7.008	6.571	7.475	59.195	0.000	0.000	0	1.000	0.000
3.	1	9.725	9.177	10.306	76.839	0.000	0.000	0	1.000	0.000

Fig S18. Meta-analysis A. Forest plot, B. Funnel plot for the Myalgia as a side effect of different COVID 19 vaccine in RCT 3.

Meta Analysis

Group by Vaccine type	Study name	Odds ratio	Lower limit	Upper limit	Z-Value	p-Value	Vaccine	Placebo
Adeno vector	Zhu et al.	4.735	1.641	13.660	2.877	0.004	34 / 253	4 / 126
Adeno vector	Zhu1 et al.	3.418	1.083	10.785	2.096	0.036	13 / 129	4 / 126
Adeno vector	Folegatti1 et al.	4.081	2.907	5.731	8.123	0.000	166 / 543	52 / 534
Adeno vector		4.081	2.990	5.570	8.859	0.000	213 / 925	60 / 786
Pro-Subunit	Keech9 et al.	0.438	0.037	5.177	-0.656	0.512	1 / 25	2 / 23
Pro-Subunit	Keech10 et al.	3.868	0.714	20.956	1.569	0.117	7 / 26	2 / 23
Pro-Subunit	Keech11 et al.	1.432	0.217	9.444	0.373	0.709	3 / 25	2 / 23
Pro-Subunit	Keech12 et al.	0.420	0.036	4.963	-0.688	0.491	1 / 26	2 / 23
Pro-Subunit		1.343	0.470	3.839	0.550	0.582	12 / 102	8 / 92
RNA	Chu et al.	23.222	3.043	177.207	3.033	0.002	19 / 100	1 / 100
RNA	Chu2 et al.	33.000	4.372	249.057	3.391	0.001	25 / 100	1 / 100
RNA	Mulligan2 et al.	1.167	0.151	9.006	0.148	0.882	3 / 12	2 / 9
RNA		9.672	1.216	76.901	2.145	0.032	47 / 212	4 / 209
Overall		3.804	2.831	5.110	8.868	0.000	272 / 1239	72 / 1087

Odds ratio and 95% CI

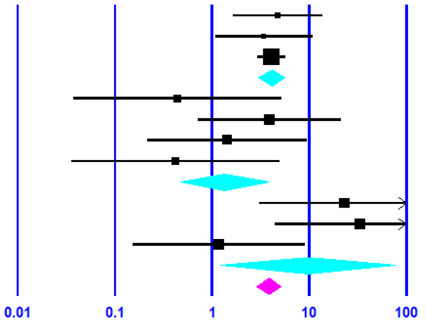

Groups		Effect size and 95% interval			Test of null (2-Tail)		Heterogeneity			
Group	Number Studies	Point estimate	Lower limit	Upper limit	Z-value	P-value	Q-value	df (Q)	P-value	I-squared
**Mixed effects analysis**										
Adeno vector	3	4.081	2.990	5.570	8.859	0.000	0.167	2	0.920	0.000
Pro-Subunit	4	1.343	0.470	3.839	0.550	0.582	3.152	3	0.369	4.833
RNA	3	9.672	1.216	76.901	2.145	0.032	6.244	2	0.044	67.970

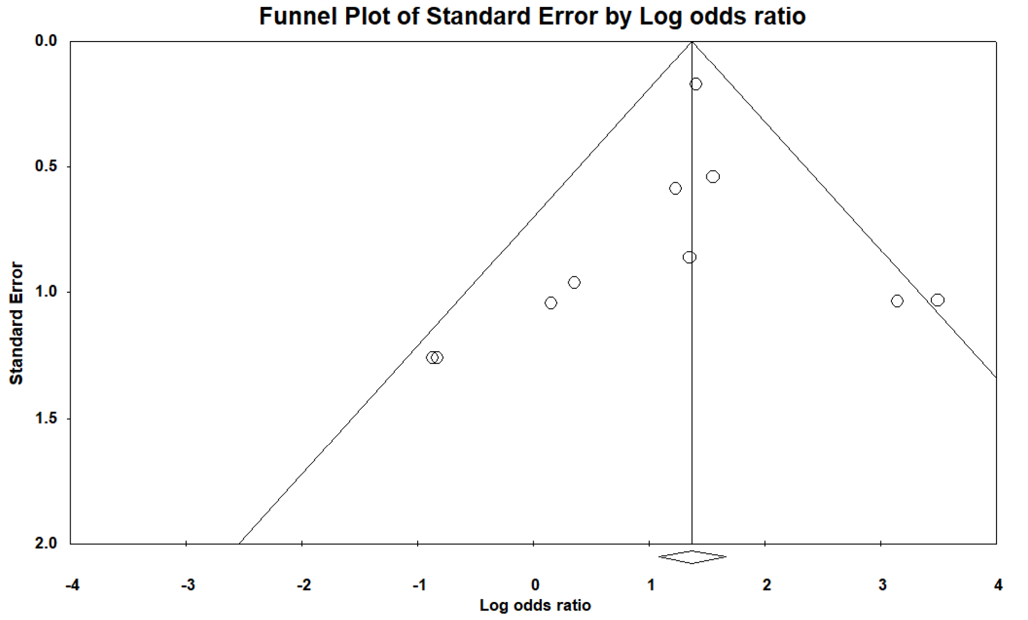

Fig S19. Meta-analysis A. Forest plot, B. Funnel plot for the Arthralgia as a side effect of different COVID 19 vaccine in RCT 2/3.

A

Meta Analysis

Group by Vaccine type	Study name	Odds ratio	Lower limit	Upper limit	Z-Value	p-Value	Vaccine	Placebo
		Statistics for each study					Events / Total	
Adeno vector	Folegatti1 et al.	10.617	7.601	14.830	13.857	0.000	287 / 543	51 / 534
Adeno vector		10.617	7.601	14.830	13.857	0.000	287 / 543	51 / 534
RNA	Chu et al.	21.000	4.858	90.774	4.076	0.000	30 / 100	2 / 100
RNA	Chu1 et al.	8.083	2.318	28.187	3.279	0.001	20 / 100	3 / 100
RNA	Chu2 et al.	36.965	8.629	158.356	4.863	0.000	43 / 100	2 / 100
RNA	Chu3 et al.	14.527	4.269	49.432	4.283	0.000	31 / 100	3 / 100
RNA	Walsh11 et al.	0.727	0.039	13.452	-0.214	0.831	1 / 12	1 / 9
RNA	Walsh13 et al.	5.714	0.532	61.410	1.439	0.150	5 / 12	1 / 9
RNA	Walsh15 et al.	11.200	1.042	120.363	1.994	0.046	7 / 12	1 / 9
RNA	Mulligan2 et al.	17.500	1.968	155.592	2.567	0.010	10 / 12	2 / 9
RNA		13.112	7.195	23.894	8.405	0.000	147 / 448	15 / 436
Overall		11.161	8.335	14.945	16.195	0.000	434 / 991	66 / 970

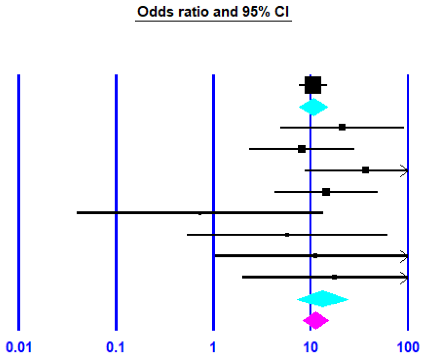

Group	Number Studies	Point estimate	Lower limit	Upper limit	Z-value	P-value	Q-value	df (Q)	P-value	I-squared
Groups		Effect size and 95% interval			Test of null (2-Tail)		Heterogeneity			
Mixed effects analysis										
Adeno vector	1	10.617	7.601	14.830	13.857	0.000	0.000	0	1.000	0.000
RNA	8	13.112	7.195	23.894	8.405	0.000	7.278	7	0.400	3.823

B

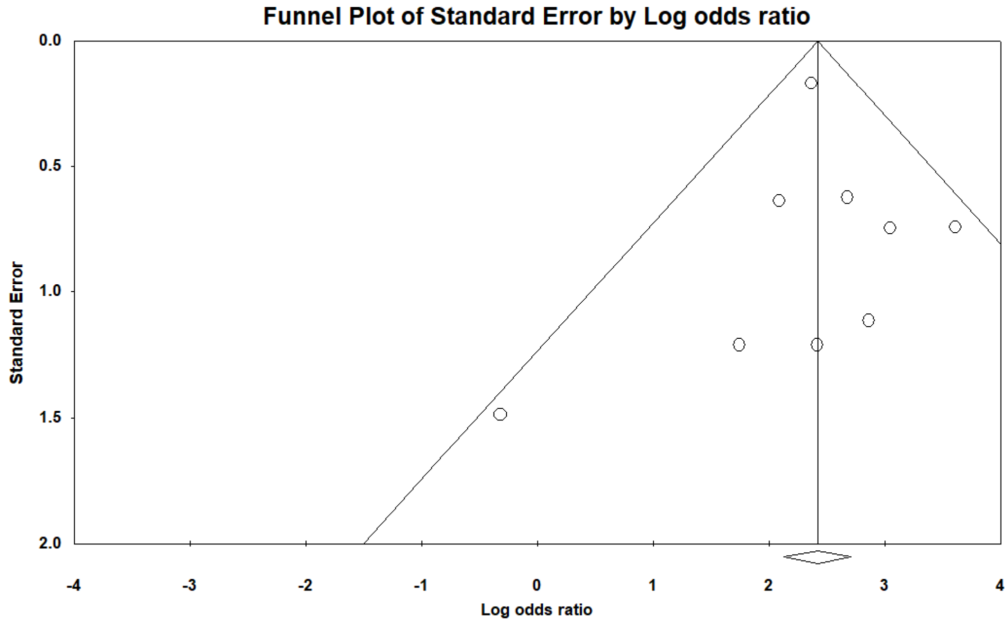

Fig S20. Meta-analysis A. Forest plot, B. Funnel plot for the Chills as a side effect of different COVID 19 vaccine in RCT 2/3.

Meta Analysis

Group by Vaccine type	Study name	Statistics for each study					Events / Total	
		Odds ratio	Lower limit	Upper limit	Z-Value	p-Value	Vaccine	Placebo
Adeno vector	Zhu et al.	0.486	0.153	1.538	-1.228	0.220	6 / 253	6 / 126
Adeno vector	Zhu1 et al.	0.640	0.176	2.324	-0.678	0.498	4 / 129	6 / 126
Adeno vector		0.549	0.232	1.297	-1.367	0.171	10 / 382	12 / 252
RNA	Mulligan2 et al.	17.500	1.968	155.592	2.567	0.010	10 / 12	2 / 9
RNA		17.500	1.968	155.592	2.567	0.010	10 / 12	2 / 9
Overall		0.873	0.392	1.942	-0.333	0.739	20 / 394	14 / 261

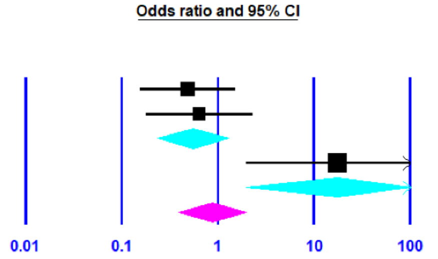

Groups		Effect size and 95% interval			Test of null (2-Tail)		Heterogeneity			
Group	Number Studies	Point estimate	Lower limit	Upper limit	Z-value	P-value	Q-value	df (Q)	P-value	I-squared
Mixed effects analysis										
Adeno vector	2	0.549	0.232	1.297	-1.367	0.171	0.098	1	0.755	0.000
RNA	1	17.500	1.968	155.592	2.567	0.010	0.000	0	1.000	0.000

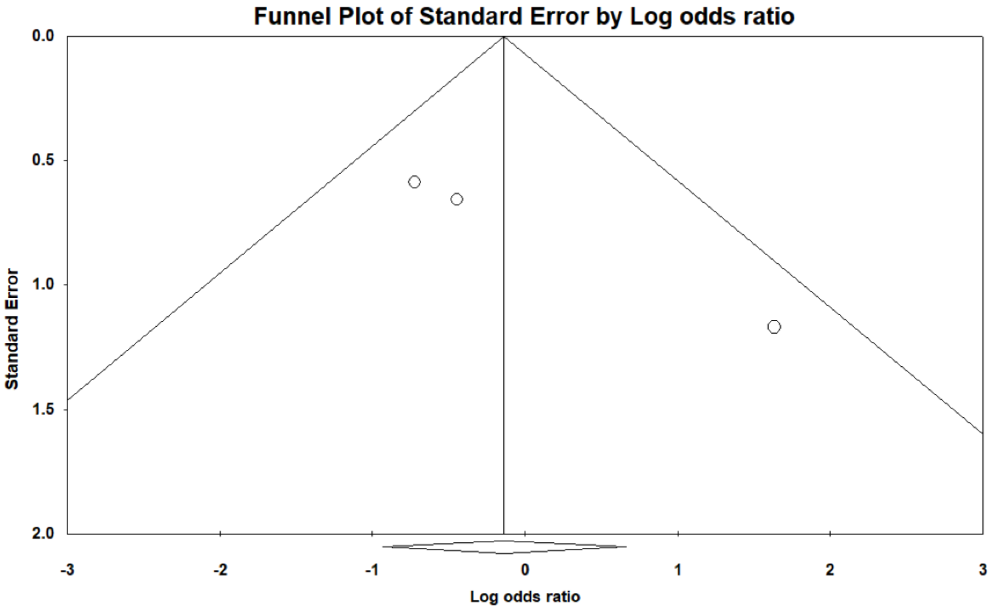

Fig S21. Meta-analysis A. Forest plot, B. Funnel plot for the Pruritus as a side effect of different COVID 19 vaccine in RCT 2/3.

Meta Analysis

Group by Adjuvant	Study name	Odds ratio	Lower limit	Upper limit	Z-Value	p-Value	Vaccine	Placebo
		Statistics for each study					Events / Total	
Aluminum hydroxide	Xia.3 et al.	0.491	0.043	5.616	-0.572	0.567	2 / 112	1 / 28
Aluminum hydroxide	Xia.4 et al.	0.075	0.007	0.752	-2.202	0.028	1 / 112	3 / 28
Aluminum hydroxide	Xia.5 et al.	0.243	0.015	4.014	-0.988	0.323	1 / 112	1 / 28
Aluminum hydroxide	Zhang. et al.	0.322	0.052	1.981	-1.222	0.222	2 / 120	3 / 60
Aluminum hydroxide	Zhang.2 et al.	0.322	0.052	1.981	-1.222	0.222	2 / 120	3 / 60
Aluminum hydroxide	Zhang et al.	0.455	0.075	2.756	-0.857	0.391	2 / 24	4 / 24
Aluminum hydroxide	Zhang2 et al.	1.000	0.219	4.564	0.000	1.000	4 / 24	4 / 24
Aluminum hydroxide		0.392	0.187	0.820	-2.486	0.013	14 / 624	19 / 252
Blank	Chu et al.	3.273	1.745	6.137	3.696	0.000	45 / 100	20 / 100
Blank	Chu1 et al.	5.785	3.106	10.775	5.531	0.000	62 / 100	22 / 100
Blank	Chu2 et al.	7.429	3.919	14.081	6.146	0.000	65 / 100	20 / 100
Blank	Chu3 et al.	6.584	3.518	12.322	5.895	0.000	65 / 100	22 / 100
Blank	Walsh5 et al.	6.000	1.018	35.374	1.979	0.048	8 / 12	3 / 12
Blank	Walsh6 et al.	1.167	0.151	9.006	0.148	0.882	3 / 12	2 / 9
Blank	Walsh7 et al.	15.000	2.024	111.174	2.650	0.008	10 / 12	3 / 12
Blank	Walsh8 et al.	4.900	0.700	34.300	1.601	0.109	7 / 12	2 / 9
Blank	Walsh9 et al.	15.000	2.024	111.174	2.650	0.008	10 / 12	3 / 12
Blank	Walsh10 et al.	7.000	0.969	50.567	1.929	0.054	8 / 12	2 / 9
Blank	Walsh11 et al.	0.400	0.067	2.372	-1.009	0.313	4 / 12	5 / 9
Blank	Walsh12 et al.	0.318	0.024	4.202	-0.870	0.384	1 / 12	2 / 9
Blank	Walsh13 et al.	1.120	0.196	6.414	0.127	0.899	7 / 12	5 / 9
Blank	Walsh14 et al.	1.750	0.242	12.642	0.555	0.579	4 / 12	2 / 9
Blank	Walsh15 et al.	2.400	0.376	15.319	0.926	0.355	9 / 12	5 / 9
Blank	Walsh16 et al.	1.167	0.151	9.006	0.148	0.882	3 / 12	2 / 9
Blank	Mulligan et al.	7.000	0.969	50.567	1.929	0.054	8 / 12	2 / 9
Blank	Mulligan1 et al.	17.500	1.968	155.592	2.567	0.010	10 / 12	2 / 9
Blank	Mulligan2 et al.	17.500	1.968	155.592	2.567	0.010	10 / 12	2 / 9
Blank		4.266	2.834	6.421	6.953	0.000	339 / 580	126 / 544
Matrix-M1	Keech10 et al.	5.714	1.357	24.064	2.376	0.017	12 / 26	3 / 23
Matrix-M1	Keech11 et al.	6.154	1.451	26.105	2.465	0.014	12 / 25	3 / 23
Matrix-M1	Keech12 et al.	1.212	0.241	6.093	0.233	0.815	4 / 26	3 / 23
Matrix-M1		3.705	1.369	10.027	2.578	0.010	28 / 77	9 / 69
No adjuvant	Zhu et al.	3.605	2.120	6.131	4.734	0.000	106 / 253	21 / 126
No adjuvant	Zhu1 et al.	2.588	1.430	4.685	3.142	0.002	44 / 129	21 / 126
No adjuvant	Kremsner et al.	2.065	0.655	6.511	1.237	0.216	13 / 47	5 / 32
No adjuvant	Kremsner1 et al.	5.870	1.935	17.804	3.126	0.002	25 / 48	5 / 32
No adjuvant	Kremsner2 et al.	7.560	2.483	23.020	3.561	0.000	28 / 48	5 / 32
No adjuvant	Kremsner3 et al.	16.691	5.172	53.867	4.709	0.000	34 / 45	5 / 32
No adjuvant		4.433	2.622	7.497	5.556	0.000	250 / 570	62 / 380
Overall		3.001	2.260	3.985	7.599	0.000	631 / 1851	216 / 1245

Odds ratio and 95% CI

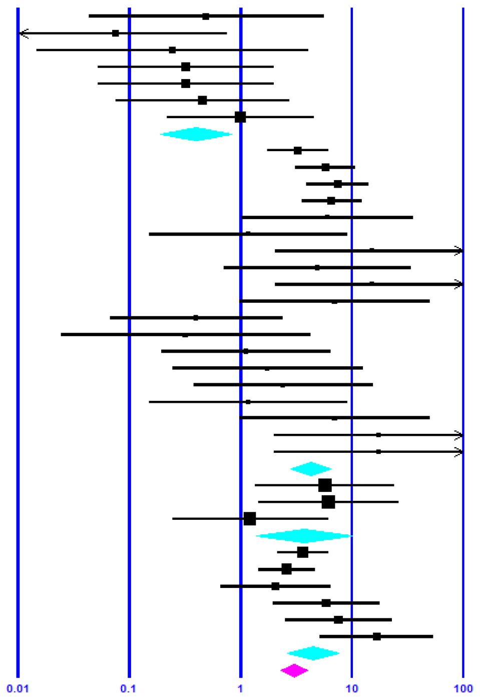

Groups		Effect size and 95% interval			Test of null (2-Tail)		Heterogeneity			
Group	Number Studies	Point estimate	Lower limit	Upper limit	Z-value	P-value	Q-value	df (Q)	P-value	I-squared
Mixed effects analysis										
Aluminum	7	0.392	0.187	0.820	-2.486	0.013	3.698	6	0.718	0.000
Blank	19	4.266	2.834	6.421	6.953	0.000	29.680	18	0.041	39.354
Matrix-M1	3	3.705	1.369	10.027	2.578	0.010	2.660	2	0.264	24.811
No adjuvant	6	4.433	2.622	7.497	5.556	0.000	10.890	5	0.054	54.088

Fig S22. Meta-analysis A. Forest plot, B. Funnel plot for the Fatigue as a side effect based on the adjuvant type in phase 2/3 RCT.

Meta Analysis

Group by adjuvant	Study name	Odds ratio	Lower limit	Upper limit	Z-Value	p-Value	Vaccine	Placebo
Aluminum hydroxide	Xia.3 et al.	0.491	0.043	5.616	-0.572	0.567	2 / 112	1 / 28
Aluminum hydroxide	Zhang. et al.	0.496	0.030	8.067	-0.493	0.622	1 / 120	1 / 60
Aluminum hydroxide	Zhang et al.	1.000	0.059	16.970	0.000	1.000	1 / 24	1 / 24
Aluminum hydroxide		0.608	0.130	2.835	-0.634	0.526	4 / 256	3 / 112
Blank	Mulligan2 et al.	1.750	0.242	12.642	0.555	0.579	4 / 12	2 / 9
Blank		1.750	0.242	12.642	0.555	0.579	4 / 12	2 / 9
No adjuvant	Zhu et al.	2.476	0.824	7.441	1.616	0.106	19 / 253	4 / 126
No adjuvant	Zhu1 et al.	2.563	0.782	8.397	1.554	0.120	10 / 129	4 / 126
No adjuvant	Kremsner1 et al.	0.235	0.043	1.295	-1.664	0.096	2 / 48	5 / 32
No adjuvant	Kremsner2 et al.	0.628	0.166	2.374	-0.686	0.493	5 / 48	5 / 32
No adjuvant	Kremsner3 et al.	0.386	0.085	1.747	-1.236	0.217	3 / 45	5 / 32
No adjuvant	Kremsner4 et al.	0.648	0.140	2.996	-0.555	0.579	3 / 28	5 / 32
No adjuvant		0.892	0.403	1.973	-0.283	0.777	42 / 551	28 / 380
Overall		0.896	0.461	1.742	-0.324	0.746	50 / 819	33 / 501

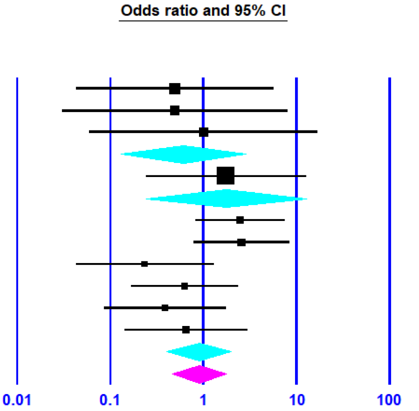

Group	Number Studies	Point estimate	Lower limit	Upper limit	Z-value	P-value	Q-value	df (Q)	P-value	I-squared
**Mixed effects analysis**										
Aluminum	3	0.608	0.130	2.835	-0.634	0.526	0.169	2	0.919	0.000
Blank	1	1.750	0.242	12.642	0.555	0.579	0.000	0	1.000	0.000
No adjuvant	6	0.892	0.403	1.973	-0.283	0.777	10.096	5	0.073	50.478

(Column groups: Groups; Effect size and 95% interval; Test of null (2-Tail); Heterogeneity)

Fig S23. Meta-analysis A. Forest plot, B. Funnel plot for the Diarrhea as a side effect based on the adjuvant type in phase 2/3 RCT

Meta Analysis

Group by Adjuvant	Study name	Odds ratio	Lower limit	Upper limit	Z-Value	p-Value	Vaccine	Placebo
		Statistics for each study					site pain / Total	
Aluminum hydroxide	Yang et al.	0.828	0.247	2.773	-0.307	0.759	5 / 150	6 / 150
Aluminum hydroxide	Yang1 et al.	1.352	0.458	3.996	0.546	0.585	8 / 150	6 / 150
Aluminum hydroxide	Yang2 et al.	3.955	1.428	10.950	2.646	0.008	18 / 150	5 / 150
Aluminum hydroxide	Yang3 et al.	3.955	1.428	10.950	2.646	0.008	18 / 150	5 / 150
Aluminum hydroxide	Xia. et al.	0.203	0.032	1.285	-1.693	0.090	2 / 84	3 / 28
Aluminum hydroxide	Xia.1 et al.	4.500	0.558	36.287	1.412	0.158	12 / 84	1 / 28
Aluminum hydroxide	Zhang. et al.	4.462	0.991	20.089	1.948	0.051	16 / 120	2 / 60
Aluminum hydroxide	Zhang.1 et al.	0.744	0.121	4.574	-0.320	0.749	3 / 120	2 / 60
Aluminum hydroxide	Zhang.2 et al.	3.830	0.841	17.439	1.736	0.082	14 / 120	2 / 60
Aluminum hydroxide	Zhang.3 et al.	1.796	0.362	8.925	0.716	0.474	7 / 120	2 / 60
Aluminum hydroxide	Yang. et al.	1.000	0.150	6.671	0.000	1.000	4 / 20	2 / 10
Aluminum hydroxide	Yang.1 et al.	4.889	0.822	29.060	1.745	0.081	11 / 20	2 / 10
Aluminum hydroxide	Xia et al.	2.895	0.503	16.674	1.190	0.234	5 / 24	2 / 24
Aluminum hydroxide	Xia1 et al.	0.478	0.040	5.658	-0.585	0.558	1 / 24	2 / 24
Aluminum hydroxide	Xia2 et al.	3.667	0.658	20.421	1.483	0.138	6 / 24	2 / 24
Aluminum hydroxide	Xia3 et al.	2.739	0.294	25.537	0.885	0.376	9 / 32	1 / 8
Aluminum hydroxide	Xia4 et al.	0.226	0.013	4.065	-1.009	0.313	1 / 32	1 / 8
Aluminum hydroxide	Xia5 et al.	1.960	0.205	18.721	0.584	0.559	7 / 32	1 / 8
Aluminum hydroxide	Zhang et al.	26.714	4.909	145.377	3.801	0.000	17 / 24	2 / 24
Aluminum hydroxide	Zhang1 et al.	1.333	0.264	6.739	0.348	0.728	4 / 24	3 / 23
Aluminum hydroxide	Zhang2 et al.	26.714	4.909	145.377	3.801	0.000	17 / 24	2 / 24
Aluminum hydroxide	Zhang3 et al.	2.745	0.613	12.292	1.320	0.187	7 / 24	3 / 23
Aluminum hydroxide		2.407	1.512	3.832	3.703	0.000	192 / 1552	57 / 1106
Blank	Chu et al.	40.444	17.424	93.881	8.611	0.000	80 / 100	9 / 100
Blank	Chu1 et al.	96.000	31.519	292.397	8.032	0.000	80 / 100	4 / 100
Blank	Chu2 et al.	192.111	62.034	594.939	9.117	0.000	95 / 100	9 / 100
Blank	Chu3 et al.	276.000	80.365	947.875	8.928	0.000	92 / 100	4 / 100
Blank	Walsh5 et al.	25.000	2.921	213.986	2.938	0.003	10 / 12	2 / 12
Blank	Walsh7 et al.	55.000	4.300	703.432	3.082	0.002	11 / 12	2 / 12
Blank	Walsh9 et al.	105.000	4.521	2438.704	2.900	0.004	12 / 12	2 / 12
Blank	Walsh11 et al.	4.900	0.700	34.300	1.601	0.109	7 / 12	2 / 9
Blank	Walsh12 et al.	4.000	0.363	44.113	1.132	0.258	4 / 12	1 / 9
Blank	Walsh13 et al.	17.500	1.968	155.592	2.567	0.010	10 / 12	2 / 9
Blank	Walsh14 et al.	11.200	1.042	120.363	1.994	0.046	7 / 12	1 / 9
Blank	Walsh15 et al.	17.500	1.968	155.592	2.567	0.010	10 / 12	2 / 9
Blank	Walsh16 et al.	16.000	1.451	176.451	2.264	0.024	8 / 12	1 / 9
Blank	Mulligan et al.	17.500	1.968	155.592	2.567	0.010	10 / 12	2 / 9
Blank	Mulligan1 et al.	75.000	3.155	1782.778	2.671	0.008	12 / 12	2 / 9
Blank	Mulligan2 et al.	75.000	3.155	1782.778	2.671	0.008	12 / 12	2 / 9
Blank		40.137	20.831	77.339	11.034	0.000	460 / 544	47 / 517
No adjuvant	Zhu et al.	14.036	7.205	27.344	7.764	0.000	145 / 253	11 / 126
No adjuvant	Zhu1 et al.	13.206	6.496	26.847	7.129	0.000	72 / 129	11 / 126
No adjuvant	Kremsner et al.	32.348	4.074	256.819	3.289	0.001	24 / 47	1 / 32
No adjuvant	Kremsner1 et al.	75.286	9.346	606.446	4.060	0.000	34 / 48	1 / 32
No adjuvant	Kremsner2 et al.	51.667	6.485	411.622	3.726	0.000	30 / 48	1 / 32
No adjuvant	Kremsner3 et al.	95.818	11.685	785.747	4.250	0.000	34 / 45	1 / 32
No adjuvant	Kremsner4 et al.	113.667	12.767	1011.960	4.243	0.000	22 / 28	1 / 32
No adjuvant		25.618	13.311	49.304	9.710	0.000	361 / 598	27 / 412
Overall		8.823	6.354	12.250	13.003	0.000	1013 / 2694	131 / 2035

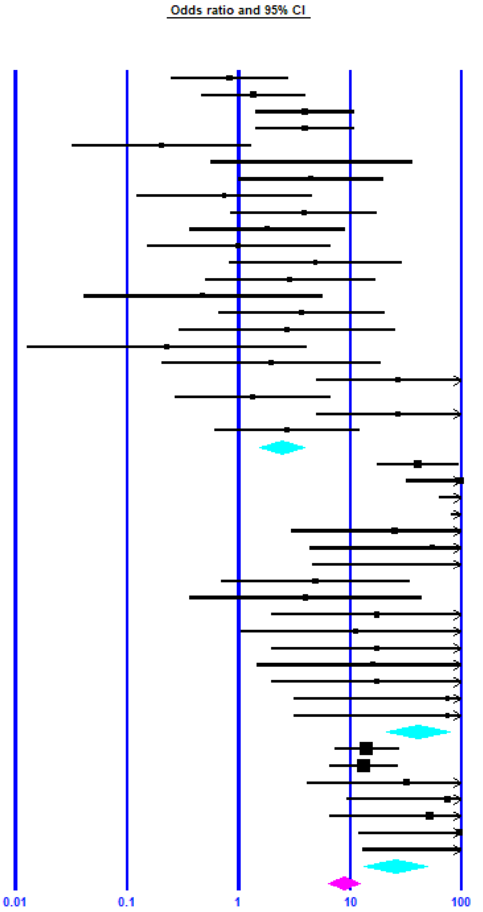

Groups		Effect size and 95% interval			Test of null (2-Tail)		Heterogeneity			
Group	Number Studies	Point estimate	Lower limit	Upper limit	Z-value	P-value	Q-value	df (Q)	P-value	I-squared
Mixed effects analysis										
Aluminum	22	2.407	1.512	3.832	3.703	0.000	37.872	21	0.013	44.551
Blank	16	40.137	20.831	77.339	11.034	0.000	29.855	15	0.012	49.757
No adjuvant	7	25.618	13.311	49.304	9.710	0.000	9.464	6	0.149	36.601

Fig S24. Meta-analysis A. Forest plot, B. Funnel plot for the Injection site pain as a side effect based on the adjuvant type in phase 2/3 RCT

Meta Analysis

Group by Adjuvant	Study name	Odds ratio	Lower limit	Upper limit	Z-Value	p-Value	Itch / Total: Vaccine	Itch / Total: Placebo
Aluminum hydroxide	Yang et al.	20.208	1.165	350.431	2.065	0.039	9 / 150	0 / 150
Aluminum hydroxide	Yang1 et al.	31.974	1.889	541.113	2.401	0.016	14 / 150	0 / 150
Aluminum hydroxide	Yang2 et al.	73.082	4.420	1208.322	2.998	0.003	29 / 150	0 / 150
Aluminum hydroxide	Yang3 et al.	64.068	3.865	1061.928	2.904	0.004	26 / 150	0 / 150
Aluminum hydroxide	Xia.1 et al.	0.325	0.020	5.380	-0.785	0.433	1 / 84	1 / 28
Aluminum hydroxide	Yang. et al.	5.727	0.279	117.648	1.132	0.258	4 / 20	0 / 10
Aluminum hydroxide	Yang.1 et al.	11.667	0.596	228.349	1.619	0.105	7 / 20	0 / 10
Aluminum hydroxide		13.203	3.234	53.909	3.595	0.000	90 / 724	1 / 648
Blank	Folegatti1 et al.	3.103	1.966	4.896	4.866	0.000	77 / 543	27 / 534
Blank		3.103	1.966	4.896	4.866	0.000	77 / 543	27 / 534
Overall		3.561	2.308	5.495	5.738	0.000	167 / 1267	28 / 1182

Odds ratio and 95% CI

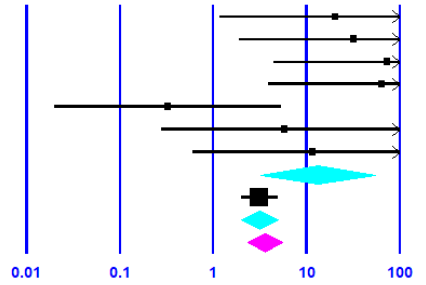

Groups		Effect size and 95% interval			Test of null (2-Tail)		Heterogeneity			
Group	Number Studies	Point estimate	Lower limit	Upper limit	Z-value	P-value	Q-value	df (Q)	P-value	I-squared
Mixed effects analysis										
Aluminum	7	13.203	3.234	53.909	3.595	0.000	10.099	6	0.121	40.589
Blank	1	3.103	1.966	4.896	4.866	0.000	0.000	0	1.000	0.000

Fig S25. Meta-analysis A. Forest plot, B. Funnel plot for the Itch as a side effect based on the adjuvant type in phase 2/3 RCT

Meta Analysis

Study name		Odds ratio	Lower limit	Upper limit	Z-Value	p-Value	Vaccine	Placebo
Aluminum hydroxide	Zhang.2 et al.	1.000	0.089	11.254	0.000	1.000	2 / 120	1 / 60
Aluminum hydroxide	Zhang2 et al.	2.091	0.177	24.734	0.585	0.558	2 / 24	1 / 24
Aluminum hydroxide		1.435	0.255	8.088	0.410	0.682	4 / 144	2 / 84
AS03	Richmond1 et al.	17.000	1.471	196.426	2.269	0.023	4 / 8	1 / 18
AS03	Richmond6 et al.	5.667	0.432	74.379	1.321	0.187	2 / 8	1 / 18
AS03	Richmond11 et al.	28.333	2.389	336.008	2.650	0.008	5 / 8	1 / 18
AS03		14.331	3.391	60.566	3.620	0.000	11 / 24	3 / 54
Blank	Chu et al.	11.819	4.732	29.519	5.288	0.000	43 / 100	6 / 100
Blank	Chu1 et al.	4.889	2.371	10.082	4.298	0.000	40 / 100	12 / 100
Blank	Chu2 et al.	22.545	9.016	56.372	6.663	0.000	59 / 100	6 / 100
Blank	Chu3 et al.	6.769	3.297	13.900	5.209	0.000	48 / 100	12 / 100
Blank	Mulligan2 et al.	4.900	0.700	34.300	1.601	0.109	7 / 12	2 / 9
Blank		8.746	4.914	15.564	7.374	0.000	197 / 412	38 / 409
CpG/Alum	Richmond3 et al.	2.429	0.133	44.501	0.598	0.550	1 / 8	1 / 18
CpG/Alum		2.429	0.133	44.501	0.598	0.550	1 / 8	1 / 18
Matrix-M1	Keech10 et al.	1.212	0.241	6.093	0.233	0.815	4 / 26	3 / 23
Matrix-M1	Keech11 et al.	7.222	1.703	30.637	2.682	0.007	13 / 25	3 / 23
Matrix-M1	Keech12 et al.	0.267	0.026	2.764	-1.108	0.268	1 / 26	3 / 23
Matrix-M1		1.576	0.261	9.499	0.496	0.620	18 / 77	9 / 69
No adjuvant	Zhu et al.	7.472	2.262	24.686	3.298	0.001	39 / 253	3 / 126
No adjuvant	Zhu1 et al.	8.896	2.598	30.462	3.480	0.001	23 / 129	3 / 126
No adjuvant	Kremsner et al.	5.425	0.634	46.440	1.544	0.123	7 / 47	1 / 32
No adjuvant	Kremsner1 et al.	14.091	1.756	113.102	2.490	0.013	15 / 48	1 / 32
No adjuvant	Kremsner2 et al.	28.520	3.598	226.079	3.172	0.002	23 / 48	1 / 32
No adjuvant	Kremsner3 et al.	24.800	3.110	197.782	3.031	0.002	20 / 45	1 / 32
No adjuvant	Kremsner4 et al.	65.444	7.673	558.165	3.823	0.000	19 / 28	1 / 32
No adjuvant	Keech9 et al.	0.580	0.088	3.825	-0.566	0.571	2 / 25	3 / 23
No adjuvant		9.663	3.977	23.478	5.007	0.000	148 / 623	14 / 435
Overall		7.400	4.835	11.325	9.218	0.000	379 / 1288	67 / 1069

Statistics for each study; Events / Total

Odds ratio and 95% CI

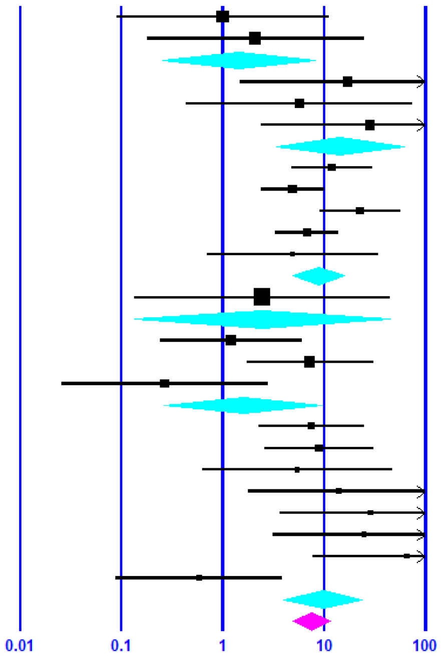

Groups		Effect size and 95% interval			Test of null (2-Tail)		Heterogeneity			
Group	Number Studies	Point estimate	Lower limit	Upper limit	Z-value	P-value	Q-value	df (Q)	P-value	I-squared
**Mixed effects analysis**										
Aluminum	2	1.435	0.255	8.088	0.410	0.682	0.175	1	0.676	0.000
AS03	3	14.331	3.391	60.566	3.620	0.000	0.809	2	0.667	0.000
Blank	5	8.746	4.914	15.564	7.374	0.000	7.781	4	0.100	48.595
CpG/Alum	1	2.429	0.133	44.501	0.598	0.550	0.000	0	1.000	0.000
Matrix-M1	3	1.576	0.261	9.499	0.496	0.620	6.244	2	0.044	67.969
No adjuvant	8	9.663	3.977	23.478	5.007	0.000	13.998	7	0.051	49.994

Fig S26. Meta-analysis A. Forest plot, B. Funnel plot for the Myalgia as a side effect based on the adjuvant type in phase 2/3 RCT

Meta Analysis

Study name		Odds ratio	Lower limit	Upper limit	Z-Value	p-Value	Vaccine (Swelling / Total)	Placebo (Swelling / Total)
Aluminum hydroxide	Yang et al.	3.083	0.612	15.528	1.365	0.172	6 / 150	2 / 150
Aluminum hydroxide	Yang1 et al.	4.723	1.003	22.242	1.964	0.050	9 / 150	2 / 150
Aluminum hydroxide	Yang2 et al.	12.047	2.771	52.366	3.319	0.001	21 / 150	2 / 150
Aluminum hydroxide	Yang3 et al.	11.385	2.611	49.639	3.237	0.001	20 / 150	2 / 150
Aluminum hydroxide	Xia.1 et al.	0.325	0.020	5.380	-0.785	0.433	1 / 84	1 / 28
Aluminum hydroxide	Zhang.3 et al.	0.496	0.030	8.067	-0.493	0.622	1 / 120	1 / 60
Aluminum hydroxide	Yang.1 et al.	1.615	0.060	43.247	0.286	0.775	1 / 20	0 / 10
Aluminum hydroxide		3.835	1.525	9.646	2.856	0.004	59 / 824	10 / 698
Blank	Chu et al.	5.211	0.598	45.426	1.494	0.135	5 / 100	1 / 100
Blank	Chu2 et al.	11.000	1.381	87.641	2.265	0.024	10 / 100	1 / 100
Blank	Folegatti1 et al.	1.210	0.642	2.284	0.590	0.555	22 / 543	18 / 534
Blank		3.099	0.708	13.574	1.501	0.133	37 / 743	20 / 734
Overall		3.612	1.652	7.899	3.217	0.001	96 / 1567	30 / 1432

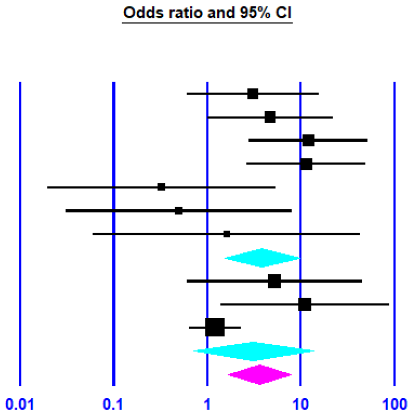

Group	Number Studies	Point estimate	Lower limit	Upper limit	Z-value	P-value	Q-value	df (Q)	P-value	I-squared
**Mixed effects analysis**										
Aluminum	7	3.835	1.525	9.646	2.856	0.004	9.604	6	0.142	37.527
Blank	3	3.099	0.708	13.574	1.501	0.133	5.199	2	0.074	61.532

(Column groups: Groups; Effect size and 95% interval; Test of null (2-Tail); Heterogeneity)

Fig S27. Meta-analysis A. Forest plot, B. Funnel plot for the Swelling as a side effect based on the adjuvant type in phase 2/3 RCT

Meta Analysis

	Study name	Statistics for each study				
		Odds ratio	Lower limit	Upper limit	Z-Value	p-Value
Aluminum hydroxide	Yang et al.	6.435	1.415	29.269	2.409	0.016
Aluminum hydroxide	Yang1 et al.	6.435	1.415	29.269	2.409	0.016
Aluminum hydroxide	Yang2 et al.	14.095	3.267	60.811	3.547	0.000
Aluminum hydroxide	Yang3 et al.	12.047	2.771	52.366	3.319	0.001
Aluminum hydroxide	Yang. et al.	2.250	0.217	23.324	0.680	0.497
Aluminum hydroxide	Yang.1 et al.	2.250	0.217	23.324	0.680	0.497
Aluminum hydroxide		7.298	3.701	14.393	5.736	0.000
No adjuvant	Zhu et al.	1.250	0.239	6.534	0.264	0.791
No adjuvant	Zhu1 et al.	0.484	0.043	5.410	-0.589	0.556
No adjuvant		0.923	0.236	3.612	-0.115	0.909
Overall		4.841	2.636	8.891	5.084	0.000

Odds ratio and 95% CI

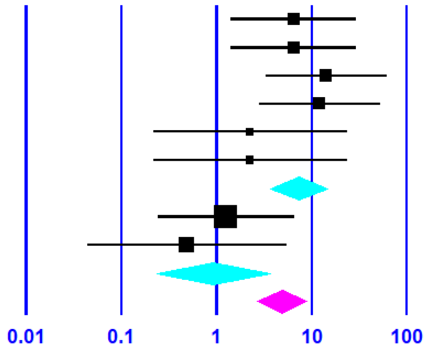

Groups		Effect size and 95% interval			Test of null (2-Tail)		Heterogeneity			
Group	Number Studies	Point estimate	Lower limit	Upper limit	Z-value	P-value	Q-value	df (Q)	P-value	I-squared
Mixed effects analysis										
Aluminum	6	7.298	3.701	14.393	5.736	0.000	3.224	5	0.666	0.000
No adjuvant	2	0.923	0.236	3.612	-0.115	0.909	0.403	1	0.525	0.000

Fig S28. Meta-analysis A. Forest plot, B. Funnel plot for the Redness as a side effect based on the adjuvant type in phase 2/3 RCT

Meta Analysis

Group by Adjuvant	Study name	Odds ratio	Lower limit	Upper limit	Z-Value	p-Value	Vaccine	Placebo
		Statistics for each study					Events / Total	
Aluminum hydroxide	Xia.1 et al.	0.325	0.020	5.380	-0.785	0.433	1 / 84	1 / 28
Aluminum hydroxide		0.325	0.020	5.380	-0.785	0.433	1 / 84	1 / 28
Blank	Chu et al.	11.494	2.600	50.819	3.220	0.001	19 / 100	2 / 100
Blank	Chu2 et al.	19.056	4.397	82.583	3.939	0.000	28 / 100	2 / 100
Blank	Mulligan2 et al.	17.500	1.968	155.592	2.567	0.010	10 / 12	2 / 9
Blank		15.310	5.969	39.271	5.677	0.000	57 / 212	6 / 209
No adjuvant	Zhu et al.	2.008	0.222	18.155	0.621	0.535	4 / 253	1 / 126
No adjuvant	Zhu1 et al.	0.977	0.060	15.784	-0.017	0.987	1 / 129	1 / 126
No adjuvant	Kremsner et al.	0.674	0.041	11.182	-0.275	0.783	1 / 47	1 / 32
No adjuvant	Kremsner1 et al.	4.429	0.507	38.681	1.346	0.178	6 / 48	1 / 32
No adjuvant	Kremsner2 et al.	4.429	0.507	38.681	1.346	0.178	6 / 48	1 / 32
No adjuvant	Kremsner3 et al.	6.703	0.794	56.568	1.748	0.080	8 / 45	1 / 32
No adjuvant	Kremsner4 et al.	10.333	1.183	90.256	2.112	0.035	7 / 28	1 / 32
No adjuvant		3.461	1.450	8.263	2.797	0.005	33 / 598	7 / 412
Overall		5.905	3.166	11.012	5.585	0.000	91 / 894	14 / 649

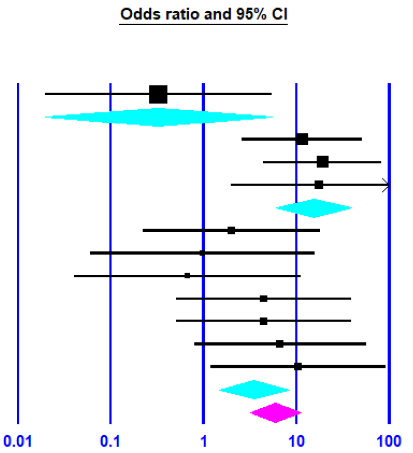

Group	Number Studies	Point estimate	Lower limit	Upper limit	Z-value	P-value	Q-value	df (Q)	P-value	I-squared
Groups		Effect size and 95% interval			Test of null (2-Tail)		Heterogeneity			
**Mixed effects analysis**										
Aluminum	1	0.325	0.020	5.380	-0.785	0.433	0.000	0	1.000	0.000
Blank	3	15.310	5.969	39.271	5.677	0.000	0.243	2	0.886	0.000
No adjuvant	7	3.461	1.450	8.263	2.797	0.005	3.779	6	0.707	0.000

Fig S29. Meta-analysis A. Forest plot, B. Funnel plot for the Vomiting as a side effect based on the adjuvant type in phase 2/3 RCT

Meta Analysis

		Statistics for each study					Events / Total	
		Odds ratio	Lower limit	Upper limit	Z-Value	p-Value	Vaccine	Placebo
Aluminum hydroxide	Yang et al.	1.352	0.458	3.996	0.546	0.585	8 / 150	6 / 150
Aluminum hydroxide	Yang1 et al.	1.899	0.684	5.276	1.231	0.218	11 / 150	6 / 150
Aluminum hydroxide	Yang2 et al.	1.000	0.434	2.303	0.000	1.000	12 / 150	12 / 150
Aluminum hydroxide	Yang3 et al.	0.910	0.388	2.132	-0.217	0.828	11 / 150	12 / 150
Aluminum hydroxide	Xia.1 et al.	0.659	0.057	7.552	-0.336	0.737	2 / 84	1 / 28
Aluminum hydroxide	Xia.2 et al.	0.243	0.015	4.014	-0.988	0.323	1 / 112	1 / 28
Aluminum hydroxide	Xia.4 et al.	0.743	0.074	7.427	-0.253	0.800	3 / 112	1 / 28
Aluminum hydroxide	Xia.5 et al.	0.152	0.024	0.955	-2.009	0.045	2 / 112	3 / 28
Aluminum hydroxide	Xia3 et al.	0.097	0.008	1.245	-1.792	0.073	1 / 32	2 / 8
Aluminum hydroxide		0.859	0.515	1.433	-0.581	0.561	51 / 1052	44 / 720
Blank	Chu et al.	11.000	1.381	87.641	2.265	0.024	10 / 100	1 / 100
Blank	Chu2 et al.	21.732	2.840	166.268	2.966	0.003	18 / 100	1 / 100
Blank		15.565	3.640	66.553	3.703	0.000	28 / 200	2 / 200
No adjuvant	Zhu et al.	4.556	2.377	8.731	4.569	0.000	82 / 253	12 / 126
No adjuvant	Zhu1 et al.	1.847	0.867	3.936	1.590	0.112	21 / 129	12 / 126
No adjuvant		2.964	1.225	7.172	2.410	0.016	103 / 382	24 / 252
Overall		1.460	0.956	2.229	1.752	0.080	182 / 1634	70 / 1172

Odds ratio and 95% CI

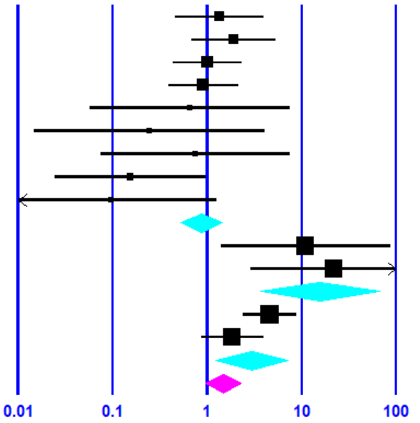

Groups		Effect size and 95% interval			Test of null (2-Tail)		Heterogeneity			
Group	Number Studies	Point estimate	Lower limit	Upper limit	Z-value	P-value	Q-value	df (Q)	P-value	I-squared
Mixed effects analysis										
Aluminum	9	0.859	0.515	1.433	-0.581	0.561	10.100	8	0.258	20.789
Blank	2	15.565	3.640	66.553	3.703	0.000	0.211	1	0.646	0.000
No adjuvant	2	2.964	1.225	7.172	2.410	0.016	3.144	1	0.076	68.197

Fig S30. Meta-analysis A. Forest plot, B. Funnel plot for the Fever as a side effect based on the adjuvant type in phase 2/3 RCT
